# From genomics to horticultural innovation: botanical traits, bioactive compounds, and sustainable utilization of kiwifruit (*Actinidia* spp.)

**DOI:** 10.3389/fpls.2026.1903424

**Published:** 2026-08-17

**Authors:** Xiao-xue Tian, Xin Liu, Tian-Yu Wang, Pei-Pei Wu, Susan L. Morris‐Natschke, Guo-Xu Ma, Xu-Dong Xu, Zi-Jian Zhao, Yuan-Xiang Li, Hai-Feng Wu, Xiang-Sheng Zhao, Qiong-Yu Zou

**Affiliations:** 1 Key Laboratory of Research and Utilization of Ethnomedicinal Plant Resources of Hunan Province, Key Laboratory of Hunan Higher Education for Western Hunan Medicinal Plant and Ethnobotany, Hunan Provincial Higher Education Key Laboratory of Intensive Processing Utilization in Wuling Mountain Area, Department of Chemistry and Chemical Engineering, Huaihua University, Huaihua, China; 2 Science and Technology Research Center of Chinese Customs, Beijing, China; 3 Natural Products Research Laboratories, University of North Carolina at Chapel Hill (UNC) Eshelman School of Pharmacy, University of North Carolina, Chapel Hill, NC, United States; 4 Beijing Key Laboratory of New Drug Discovery Based on Classic Chinese Medicine Prescription, Key Laboratory of Bioactive Substances and Resources Utilization of Chinese Herbal Medicine, Ministry of Education, Institute of Medicinal Plant Development, Chinese Academy of Medical Sciences and Peking Union Medical College, Beijing, China; 5 Hainan Provincial Key Laboratory of Resources Conservation and Development of Southern Medicine and Hainan Branch of the Institute of Medicinal Plant Development, Chinese Academy of Medical Sciences and Peking Union Medical College, Haikou, China

**Keywords:** *Actinidia chinensis*, bioactivity, botanical characteristics, genomics, horticultural innovation, kiwifruit, phytochemicals, sustainable utilization

## Abstract

Kiwifruit (Actinidia spp.), a medicine-food homologous crop with high botanical diversity, is rich in polyphenols, pectin, ascorbic acid, and phytosterols. These bioactive compounds confer diverse pharmacological activities, including antioxidation, anti-inflammation, metabolic regulation, cardiovascular protection, and antitumor effects. This review summarizes the botanical traits, resource distribution, and phytochemical profiles of *Actinidia* spp., and outlines key advances in propagation, breeding, and postharvest preservation. Recent genomic, transcriptomic, and metabolomic data are integrated to dissect gene families and regulatory networks underlying fruit quality, stress tolerance, and bioactive compound biosynthesis in *A. chinensis*. Applications in functional foods and ecological agriculture are discussed, and future directions for sustainable utilization are proposed. This work provides a foundation for germplasm innovation, molecular breeding, and high-value kiwifruit utilization.

## 1 Introduction

### 1.1 Significance and research advancements

Kiwifruit (*Actinidia* spp.) is a globally important subtropical horticultural crop with prominent economic, nutritional and medicinal value, which has attracted wide research interest in agriculture, food science and biomedicine. Though kiwifruit has a short domestication history, its planting scale expands rapidly owing to outstanding nutritional advantages, especially ultra-high vitamin C content, as well as abundant polyphenols, polysaccharides, pectin, phytosterols and other bioactive substances. It covers multiple commercially valuable species and cultivars such as *A. chinensis*, *A. delicios*a and *A. arguta*, and has evolved from ordinary fresh fruit into acknowledged functional food. A large number of *in vitro*, animal and clinical trials have verified its antioxidant, anti-inflammatory, immune-regulating, metabolism-improving and cardiovascular-protective functions, making kiwifruit a valuable raw material for functional food and pharmaceutical exploitation.

A series of systematic reviews and cultivar comparative studies have deepened the cognition of kiwifruit bioactives, nutritional variation and metabolic characteristics. Comprehensive summaries covering classification, extraction methods and processing influences of kiwifruit bioactive components have been published (Li et al., 2024a). Comparative experiments on phytochemical composition and antioxidant capacity across commercial kiwifruit varieties have been carried out (Uy et al., 2026). Research focusing on fruit physicochemical and nutritional distinctions among different cultivars is also available (Yuan et al., 2023). Simulated gastrointestinal digestion experiments have clarified the actual bioavailability of kiwifruit nutrients (Ma et al., 2019). Moreover, metabolomics research has identified cultivar-specific accumulation rules of triterpenoids in hardy kiwifruit (*A. arguta*) (Duan et al., 2026).

Existing studies have elaborated kiwifruit’s nutritional and pharmacological traits, but prior reviews rarely cover global production and trade, nor link phytochemical data to breeding practice. Different from past reviews that separately discussed phytochemistry, pharmacology or single omics, this paper fills these gaps via an integrated framework focused on kiwifruit horticultural innovation and sustainable development. Three core modules are combined: multi-omics regulation of bioactive compounds, global industrial economics, and practical genomic selection & marker-assisted breeding. This cross-disciplinary perspective breaks the lab-only bias of previous summaries and delivers comprehensive support for germplasm innovation, deep processing and sustainable industry, demonstrating the distinct novelty of the present review.

### 1.2 Breakthroughs in genomic research

Advancements in high-throughput sequencing and long-read technologies have fundamentally transformed kiwifruit research, providing a high-quality molecular foundation for dissecting key traits related to nutrient biosynthesis, stress resistance, and metabolic regulation. For instance, two haplotype-resolved, gap-free genome assemblies of *A. latifolia* and *A. chinensis* have been completed, revealing the genetic basis of critical traits such as vitamin C accumulation, sucrose metabolism, and polyphenol biosynthesis—core components underlying kiwifruit’s health-promoting effects (Han et al., 2023). These high-quality genomes enable precise identification of functional genes, such as *AcSWEET9b* (a key regulator of sucrose content) and key enzymes in the phenylpropanoid pathway (e.g., PAL, CHS), which are closely associated with the synthesis of bioactive phenolic compounds (Han et al., 2023; Kim et al., 2021).

Similarly, the telomere-to-telomere gap-free reference genome of the red-fleshed cultivar *A. chinensis* ‘Hongyang’ (Hongyang v4.0) has been successfully assembled. This high-quality assembly not only refines chromosome structure but also annotates thousands of additional protein-coding genes related to immune responses and secondary metabolite biosynthesis, laying a solid foundation for dissecting the genetic variations underlying key horticultural traits, including fruit ripening, stress resistance, and fruit quality (Yue et al., 2023).

### 1.3 Functional analysis of key gene families

Exploration of gene families associated with kiwifruit development, reproduction, and stress response has significantly deepened our understanding of its intrinsic regulatory mechanisms. A representative example is the systematic functional and evolutionary analysis of the *SOFF* and *SyGI* gene families, which are core determinants of sex differentiation in dioecious *Actinidia* species. Studies have revealed that whole-genome duplication events are a key driver of the expansion of these gene families. The proteins encoded by *SOFF* and *SyGI* not only mediate hormone signaling pathways and stress response cascades but also exert pleiotropic effects on multiple plant developmental processes. These findings not only clarify the molecular basis of kiwifruit sex determination but also provide critical target genes for genetic engineering strategies aimed at improving reproductive traits (e.g., sex control, flowering regulation) and optimizing yield potential (Zhu et al., 2025).

Beyond the molecular regulatory mechanism of floral sexual differentiation, the dual gene module of *SOFF* and *SyGI* bears profound practical significance for commercial kiwifruit breeding programs. First, species-specific molecular markers developed based on the sequence polymorphisms of *SOFF* and *SyGI* can realize early sex identification of seedling populations at the nursery stage. Breeders can cull superfluous male/female seedlings without waiting for flowering, greatly saving orchard management costs and shortening the breeding selection cycle. Second, targeted gene editing of *SyGI* and *SOFF* offers a viable technical strategy to generate monoecious kiwifruit germplasm, which resolves the issue of imbalanced male-female plant ratios in large commercial orchards and stabilizes annual fruit productivity. Third, sex-determining genotypic data can be integrated with fruit-quality QTL information to rationally assemble hybrid parental combinations, accelerating the breeding of high-yield, premium commercial cultivars and advancing standardized industrial kiwifruit breeding.

### 1.4 Global industrial and economic profile of kiwifruit

Although abundant literature has summarized the nutritional and medicinal advantages of Actinidia spp., limited reviews have comprehensively addressed its economic importance as a globally traded high-value horticultural commodity.

#### 1.4.1 Global production layout and core producing countries

Global kiwifruit planting covers Asia, Europe and Oceania. By 2024, the total cultivation area hit 287,000 ha with an annual output of 4.467 million tonnes, showing sustained growth over the past 20 years (FAO, 2025a; Zhong et al., 2023). Asia contributes 54.5% of global yield, followed by Europe (24.1%) and Oceania (14.4%); the top ten producing countries together occupy more than 98% of worldwide production (Andreev et al., 2025).

China is the world’s leading producer, possessing 199,000 ha of orchards and generating 52.4% of global output in 2024, equivalent to 68% of the total global planting area FAO, 2025a; Li et al., 2024a). Its cultivation is divided into five ecological zones growing green, red and yellow-fleshed varieties, yet the country occupies a tiny proportion of high-value export markets (Section 1.4.2). Italy, New Zealand, Chile, Greece and Iran act as major export-oriented producers: Italy operates mature industrial systems for *A. deliciosa* and newly developed *A. arguta*; New Zealand establishes a fully integrated industrial chain under the Zespri brand to obtain global price premiums; Chile delivers counter-seasonal fruit to European and North American markets. Collectively, Northern and Southern Hemisphere exporters build a year-round supply chain that complements China’s domestic-focused production system (Andreev et al., 2025).

##### 1.4.1.1 Core producing countries and regional characteristics

China ranks first globally, with 199,000 ha of orchards and an annual output of 3.8 million tonnes in 2024, accounting for 68% of global planting area and 52.4% of total yield (FAO, 2025a; Li et al., 2024a). Its production is scattered across five ecological zones with distinct cultivar layouts, yet the country gains little share in high‑end export markets (Section 1.4.2).

Italy, New Zealand, Chile, Greece and Iran are major export powers. Italy boasts mature industrial chains for both *A. deliciosa* and *A. arguta*; New Zealand relies on integrated Zespri brand operation to secure global price premiums; Chile supplies counter‑seasonal fruits to Northern markets (Andreev et al., 2025). Together, these Northern and Southern Hemisphere exporters form a year‑round supply system complementary to China’s domestic‑focused industry.

Globally, over 85% of orchards are planted with *A. chinensis* var. *chinensis* and *A. chinensis* var. *deliciosa* (Zhong et al., 2023). Cold‑hardy *A. arguta*, and nutrient‑rich *A. eriantha* are gradually commercialized, while wild *A. kolomikta* and *A. polygama* are mainly reserved as breeding germplasm in Russia and Northeast China.

#### 1.4.2 International market scale and export trade dynamics

Global fresh kiwifruit export value hit USD 4.4 billion in 2024, and the retail market is projected to reach USD 9.89 billion by 2026 (World’s Top Exports, 2025; Mordor Intelligence, 2026). A clear hemispheric staggered supply pattern supports year-round global supply: Northern Hemisphere countries supply summer markets, while New Zealand and Chile export off-season fruits from May to October (Zhong et al., 2021; Andreev et al., 2025).

Export value is highly concentrated in New Zealand (48%) and Italy (14%). Zespri’s ‘SunGold’ consistently gains global price premiums (United States Department of Agriculture Foreign Agricultural Service [USDA FAS], 2025; Zespri Group Limited, 2025). Despite occupying half of global output, China only exports 0.5% of its domestic fruit, and 91% of its imported kiwifruit comes from New Zealand (Li et al., 2024a). This unbalanced trade competitiveness will be discussed in Section 1.4.4.

#### 1.4.3 Global consumer demand characteristics

Per capita kiwifruit consumption varies drastically worldwide: European and Oceanian nations record 3–5 kg per capita annually, while China only reaches 0.8 kg despite its massive output (FAO, 2025b; Li et al., 2024a). Health and convenience drive mainstream consumption, as kiwifruit’s high vitamin C content and antioxidant and gastrointestinal benefits attract urban consumers across Europe and East Asia (Nishiyama et al., 2004; Rush et al., 2002; Richardson et al., 2018). Organic kiwifruit occupies 12–18% of the EU fresh fruit market (CBI, 2023).

Consumption preferences differ across regions: European buyers favor low-acid yellow-fleshed and organic kiwifruit; Japan and Korea prefer traditional ‘Hayward’ while accepting edible-skin A. arguta and interspecific hybrids (Costa and Testolin, 2022; Kataoka et al., 2022; Zhong et al., 2021). Red-fleshed cultivars are the most popular premium choice in Chinese cities (Li et al., 2024a). Between 2010 and 2023, red and yellow-fleshed varieties expanded from below 10% to around 35% of China’s retail kiwifruit market (China Fruit Marketing Association, 2024).

#### 1.4.4 Existing commercial bottlenecks restricting industrial upgrading

China’s kiwifruit industry accounts for more than half of global output yet faces structural commercial barriers stemming from the market‑demand mismatch described in Section 1.4.3 (Li et al., 2024a).

First, scattered smallholder cultivation lacks unified harvest standards, and widespread premature picking breaks the soluble solids content (SSC)–acid balance, resulting in inconsistent flavor that reduces consumer loyalty and retail premium potential (Li et al., 2024a).

Second, underdeveloped postharvest infrastructure widens the quality gap with overseas competitors. Insufficient pre-cooling, constant-temperature storage and automatic sorting lead to far higher postharvest losses than New Zealand (Li et al., 2024a). The NIR dry matter detection technology adopted by Zespri for standardized ripening has not been widely deployed domestically (McGlone et al., 2002).

Third, fragmented regional geographic brands lack unified grading and cross-border marketing systems. Most Chinese kiwifruit exports are low-value bulk goods with limited access to European premium retail channels (Zhong et al., 2021).

Finally, cultivar homogenization and insufficient proprietary germplasm slow industrial iteration. Domestic production relies heavily on introduced varieties, while self-bred high-quality red, yellow-fleshed and interspecific hybrids lack large-scale standardized promotion (Andreev et al., 2025). Collectively, these bottlenecks hinder China’s transformation from a major producer to a high-value competitive kiwifruit industry.

##### 1.4.4.1 Summary and outlook

In conclusion, China’s kiwifruit industry is confronted with multiple bottlenecks including decentralized cultivation, backward post‑harvest facilities, fragmented brand building and insufficient proprietary cultivars. Genomics, molecular biology and phytochemistry form an integrated research system supporting the comprehensive exploitation of *Actinidia* resources. Unraveling kiwifruit genetic networks clarifies the biosynthesis regulation of vitamin C, polyphenols and other valuable metabolites, facilitating directional molecular breeding for better fruit quality and stress resistance.

## 2 Botanical characteristics and resource distribution of kiwifruit

Advances in population genomics, cytogenetics and ecological niche modeling have deepened our understanding of botanical traits and germplasm spatial differentiation within Actinidia. Polyploidy, dioecy and shallow root architecture are well-established core evolutionary features of the genus, while uncertainties remain regarding climate warming’s impact on wild population migration and the contribution of habitat fragmentation to intraspecific genetic divergence. Integrating morphological phenotypes, chloroplast molecular markers and large-scale cytometric datasets, this chapter synthesizes interspecific morphological divergence, geographic distribution patterns and ploidy-associated population variation across *A. chinensis*, *A. deliciosa*, *A. arguta* and *A. eriantha*. We characterize interspecific adaptive differences, summarize existing limitations in wild germplasm investigations, and lay a foundational germplasm framework for subsequent sections on genomic breeding and stress tolerance improvement.

### 2.1 Morphological characteristics of the plant

*Actinidia chinensis*, commonly known as kiwifruit, is a perennial woody climbing vine that exhibits a shallow root system, which contributes to its adaptability across diverse environmental conditions. This root architecture allows the plant to efficiently exploit surface soil nutrients and water, facilitating survival and growth in variable habitats. The species displays considerable morphological diversity, especially in leaf shape and size, which is indicative of its high environmental plasticity. According to a population genetic study involving 55 kiwifruit samples, including *A. chinensis* var. *chinensis* and var. *deliciosa*, morphological observations combined with chloroplast-specific SNP markers revealed significant genetic variation that correlates with phenotypic diversity (Ding et al., 2024). Such diversity in leaf morphology likely reflects adaptive responses to different microclimates and ecological niches within its native range in China, the original speciation and distribution center for the genus *Actinidia*.

The floral morphology of *A. chinensis* is characterized by its dioecious nature, meaning that male and female flowers are borne on separate plants. This sexual dimorphism is critical for reproductive success and fruit formation. The floral organs include well-differentiated male and female structures, with male flowers producing viable pollen and female flowers equipped with receptive stigmas. The pollination mechanism in kiwifruit is primarily entomophilous, relying on insect vectors to transfer pollen from male to female flowers. The structural integrity and arrangement of floral organs influence pollination efficiency and subsequent fruit set. For instance, a study on the effect of plant growth regulator 2,4-D demonstrated that exogenous application could induce parthenocarpic fruit development in female flowers of *A. chinensis* var. ‘Donghong’ without pollination, though pollen viability was compromised (Wu et al., 2025a). This suggests that while natural pollination is essential for seed development and fruit quality, hormonal regulation can modulate fruit formation processes.

At the cellular level, reproductive development in *Actinidia* spp. (kiwifruit) involves meiosis within the anthers of male flowers. For representative species like *Actinidia chinensis*, a novel imaging method has been developed to visualize male meiotic cells in intact anthers—preserving spatial organization and enabling detailed analysis of meiotic progression (Rossig et al., 2021). This technique enhances understanding of pollen development and fertility, which are vital for breeding programs aiming to improve fruit yield and quality across the genus. Moreover, the dioecious reproductive system (in key cultivated species) combined with frequent natural hybridization events within *Actinidia* spp. contributes to the high horticultural diversity observed in kiwifruit, further underlining the importance of floral morphology and pollination biology in shaping fruit characteristics (Ding et al., 2024).

**Figure 1:**
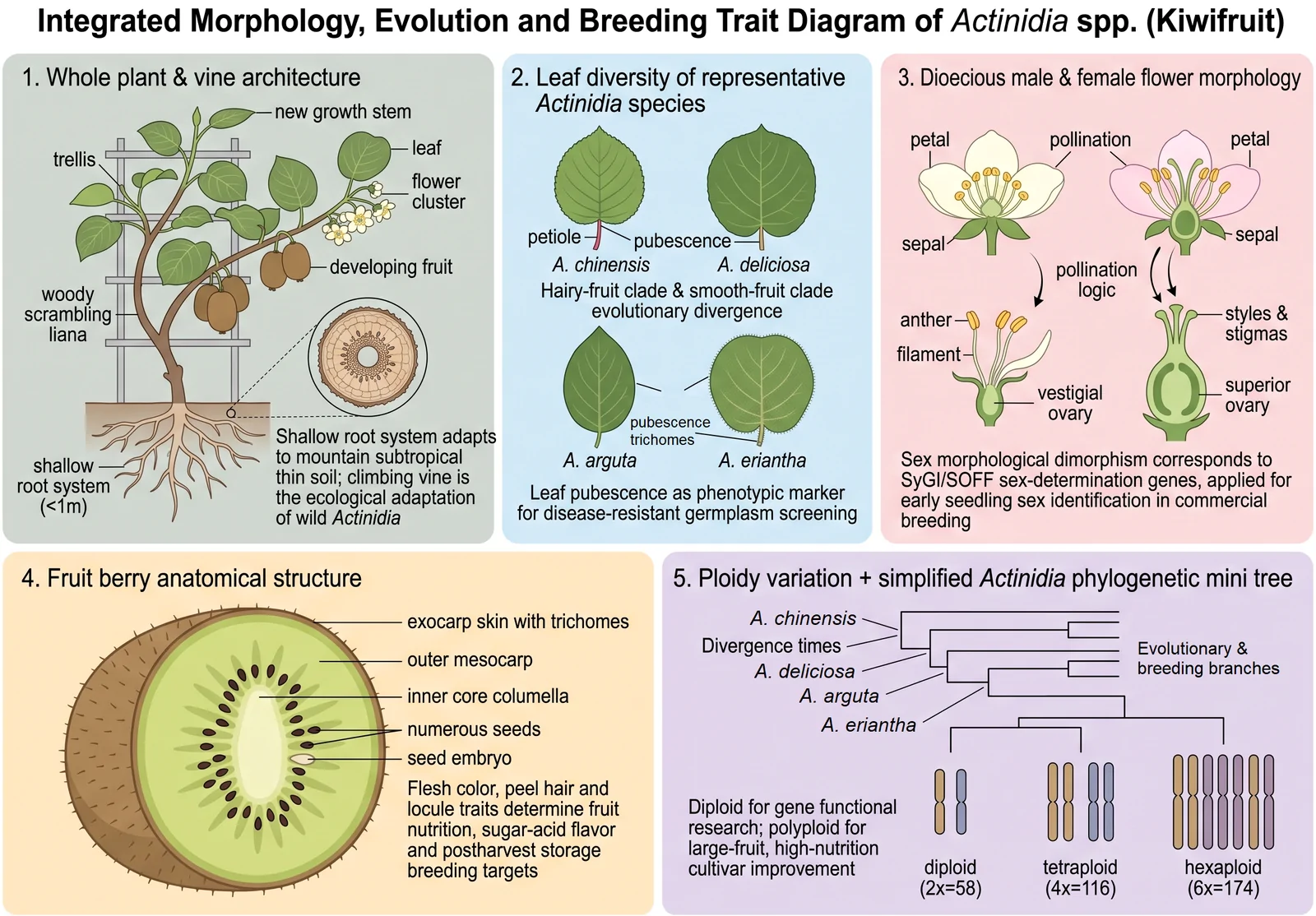
Integrated diagram of morphological, evolutionary, and breeding traits of *Actinidia*. This figure summarizes key adaptive and agronomic characteristics of kiwifruit, including the shallow-rooted liana growth form adapted to mountain habitats, leaf morphological diversity across representative species, and fruit structural traits determining nutritional and storage quality. It illustrates sexual dimorphism linked to sex-determining genes for early seedling sex discrimination, and integrates ploidy variation and phylogenetic divergence. Diploid and polyploid germplasms provide distinct genetic resources for gene functional research and cultivar improvement, respectively.

In summary, as illustrated in **Figure 1**, the perennial vine growth habit, shallow root system, and diverse leaf morphology are shared traits of *Actinidia* spp. (kiwifruit), equipping the genus with robust environmental adaptability across different ecological niches. The dioecious floral structure (in dioecious species) and insect-mediated pollination mechanism are fundamental determinants of successful fruit formation for most cultivated kiwifruit. Advances in understanding floral organ development, pollen viability, and reproductive cell biology of representative species (e.g., *A. chinensis*, *A. deliciosa*, *A. arguta*) provide valuable insights for optimizing kiwifruit breeding and cultivation strategies, supporting the improvement of reproductive traits and yield across the genus.

### 2.2 Geographical distribution and ecological adaptation

The genus *Actinidia* (kiwifruit) is primarily native to subtropical China, with commercial cultivation later established in overseas regions such as New Zealand, where mild temperature and abundant rainfall satisfy its growth demands. This taxon is highly sensitive to climate fluctuation, and suitable habitats for multiple wild kiwifruit species have gradually shifted northward over recent decades driven by global warming; altered temperature and precipitation regimes continuously reshape the ecological niches of wild *Actinidia* populations (Wang et al., 2021d).

**Figure 2:**
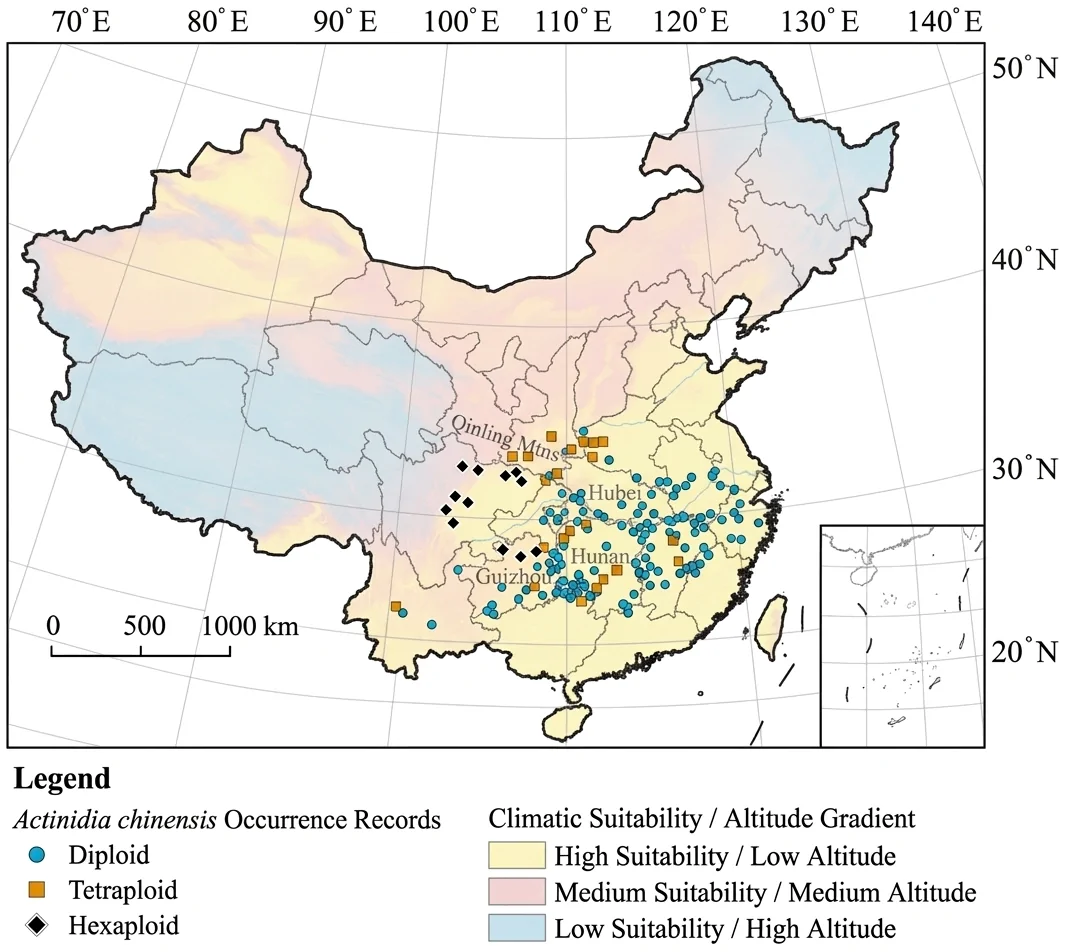
Geographical distribution and ploidy stratification of wild *Actinidia chinensis* populations in China. Diploid, tetraploid, and hexaploid populations are denoted by blue dots, orange squares, and black diamonds, respectively. The background gradient integrates altitude and climatic suitability, with yellow, pale pink, and light blue indicating high, medium, and low suitability at corresponding elevations. Key distribution zones of stratified cytotypes are highlighted, and the inset map shows the overall geographic location of the study area.

*Actinidia chinensis* exhibits highly heterogeneous geographical distribution accompanied by distinct ploidy differentiation across natural populations, as visualized in **Figure 2**. Three cytotypes form clear altitude-latitude stratification with separated distribution ranges:

1. Diploid populations (blue dots) are concentrated in low-altitude hilly zones of Hunan, Hubei and eastern Guizhou, occupying regions classified as high climatic suitability (yellow background gradient);
2. Tetraploid individuals (orange squares) scatter in transitional mid-altitude mountain areas between central and southwest China, corresponding to medium climatic suitability zones;
3. Hexaploid populations (black diamonds) are mainly restricted to the Qinling Mountains and western Guizhou Plateau at higher elevations, located within medium-to-low suitability high-altitude regions with cooler, drier conditions.

This spatial segregation demonstrates that polyploidy provides strong adaptive advantages for *A. chinensis* under harsh high-altitude, high-latitude environments featuring lower temperature and limited precipitation. Polyploid populations of *A. chinensis* possess stronger environmental tolerance than diploid counterparts. Ecological niche modeling further confirms that multi-ploidy kiwifruit populations maintain higher survival rates under cold and arid stress, revealing a ploidy-dependent niche differentiation pattern (Liu et al., 2021). Pliocene and Plio-Pleistocene paleoclimatic oscillations also profoundly molded the extant distribution and genetic architecture of *Actinidia*: repeated range contraction and expansion triggered population fragmentation and genetic divergence, which explains the high intraspecific genetic diversity of *A. chinensis* var. *chinensis* and *A. chinensis* var. *deliciosa* (Wang et al., 2021d).

Polyploidization expands genetic polymorphism and broadens the ecological amplitude of*Actinidia*, enabling the genus to colonize diverse habitats and withstand diverseabiotic stresses. Such evolutionary adaptation is particularly prominent in *A.chinensis*, supporting its wide natural distribution across subtropical China (Wang et al., 2021d; Wang et al., 2022e). Beyond climate and ploidy factors, soil properties shaped by subtropical fruit plantations also regulate kiwifruit growth. Field surveys in hilly subtropical zones indicate that *A. chinensis* orchards significantly improve soil aggregate stability, with benefits second only to Camellia oleifera forests, offering theoretical support for sustainable ecological cultivation of wild and domesticated kiwifruit (Tong et al., 2022). Topographic features such as slope position modify soilwater distribution and further influence kiwifruit yield and physiological performance under orchard conditions (Zhu et al., 2026).

In summary, the geographical distribution of *Actinidia* arises from complex interactions among ploidy-mediated adaptation, paleoclimatic evolution and edaphic conditions. The genus displays dynamic adaptive responses to ongoing climate change and retains robust evolutionary potential to accommodate future shifts in subtropical ecological environments.

### 2.3 Genetic diversity and population variation

The genetic diversity and population variation of *Actinidia* spp. (kiwifruit) are characterized by a complex interplay of polyploidy, habitat fragmentation, and gene flow—collectively contributing to the rich genetic resources available for breeding and ecological adaptation across the genus. Polyploidy is a prominent evolutionary feature of *Actinidia* spp., with diploid, tetraploid, hexaploid, and even octoploid cytotypes documented in multiple species. For representative species *Actinidia chinensis*, flow cytometry analysis of over 3,000 individuals from 128 populations has further confirmed the wide distribution of these cytotypes across its natural range. This ploidy variation is associated with significant genetic differentiation within key species (e.g., *A. chinensis* var. *chinensis* and *A. chinensis* var. *deliciosa*), which occupy distinct climatic niches and exhibit divergent evolutionary histories shaped by Pliocene and Plio-Pleistocene climatic fluctuations. Overall, the polyploidization process has likely enhanced adaptive capacity to environmental changes for *Actinidia* spp., influencing demographic history and population structure of diverse species within the genus (Wang et al., 2021d).

Habitat fragmentation impacts genetic diversity of *Actinidia* spp. (kiwifruit) in a scale-dependent manner. For representative species *Actinidia chinensis*, broad-scale sampling across mountain and island populations in China demonstrated isolation-by-distance effects, with genetic differentiation increasing with geographical distance. Notably, an oceanic island population of *A. chinensis* exhibited signs of a recent bottleneck but maintained high gene flow with a connected mountain population via the Yangtze River, indicating that habitat connectivity can mitigate fragmentation effects. At finer scales (e.g., lake islands), no negative genetic consequences were observed in *A. chinensis* populations, attributed to effective seed dispersal by water that facilitates gene flow. For this species, population size correlated positively with island area—supporting island biogeography theory—though genetic diversity did not show such a correlation, underscoring the complexity of genetic variation dynamics under fragmentation in *Actinidia* spp (Tong et al., 2022).

Molecular markers have been pivotal in deciphering the genetic architecture of *Actinidia* spp. Chloroplast-specific SNP and nSCoT analyses across 55 germplasm accessions revealed average genetic distances of 0.26 and 0.57, respectively. Haplotype analysis identified 15 distinct chlorotypes (Hd = 0.78), with the dominant H1 shared between *A. chinensis* var. *Chinensis* and var. *deliciosa*, indicating persistent inter-varietal gene flow. Conversely, *A. arguta* and *A. polygama* harbored unique haplotypes, reflecting divergent evolutionary paths. Bayesian clustering, UPGMA, and PCA consistently delineated species-specific clusters, underscoring the efficacy of these markers for germplasm characterization and core collection construction (Ding et al., 2024).

High-density SNP arrays have revolutionized genetic dissection in *Actinidia* spp., particularly for polyploid species. A custom 135K SNP array, derived from 40 diverse genotypes, facilitated the construction of an integrated linkage map and QTL analysis for key traits, including sex determination. Genotyping revealed tetrasomic inheritance with partial preferential pairing in tetraploid *A. arguta*, elucidating its complex genomic architecture. This robust tool enables fine-scale germplasm discrimination and accelerates marker-assisted breeding across kiwifruit species (Wang et al., 2023b).

Pan-genomic analyses have elucidated the role of structural variations (SVs) in driving phenotypic divergence across *Actinidia* spp. A super pan-genome constructed from 15 high-quality assemblies of eight species identified over 61,000 gene families, including core and dispensable genes governing hormone response and cell wall biogenesis. Population-scale SV profiling uncovered evolutionary hotspots linked to environmental adaptation and disease resistance. Notably, the identification of resistance gene analogs (RGAs) provides crucial targets for breeding canker-resistant cultivars, directly addressing a major threat to global kiwifruit production (Yu et al., 2025).

Collectively, polyploidy, habitat fragmentation and gene flow jointly shape the genetic architecture of *Actinidia*, with molecular markers supporting germplasm assessment. Integrated cytogenetic and genomic datasets have clarified the genus’ evolutionary routes and adaptive mechanisms to inform targeted breeding.

Major research bottlenecks still limit systematic germplasm exploitation. Existing population genetic investigations overwhelmingly focus on the *A. chinensis* complex, while rare wild species such as *A. arguta* and *A. eriantha* lack large-scale resequencing data, hindering clarification of genus-wide evolutionary patterns. Conflicting results persist regarding how isolated population size alters genetic diversity, as current studies cannot fully disentangle the independent effects of geographic isolation, pollination modes and human disturbance. In addition, pan-genome and SNP analyses largely prioritize cultivated materials, and few studies connect structural variants to agronomic QTLs to mine valuable functional loci. Furthermore, long-term field monitoring of climate-driven wild habitat shifts remains scarce.

To address these limitations, future research should conduct population resequencing on underrepresented wild kiwifruit taxa, design multi-factor controlled experiments to resolve fragmentation-related controversies and integrate pan-genomic structural variant data with field phenotyping to identify elite alleles for molecular breeding.

## 3 Chemical composition analysis of kiwifruit

Recent UPLC-MS and bioactivity screening advances have identified core bioactive metabolites in Actinidia, including triterpenoids, flavonoids, polysaccharides and vitamins. Discrepancies still exist in interspecific metabolic profiles and the *in vivo* bioavailability of pure monomer compounds. This chapter categorizes major phytochemical groups, analyzes characteristic secondary metabolites of *A. chinensis* together with their structure–activity relationships, and compares green extraction technologies by five industrial evaluation criteria to support follow-up industrial development research.

### 3.1 Major phytochemicals

The phytochemical profile of *Actinidia* spp. (kiwifruit) is notably rich and diverse, encompassing several classes of bioactive compounds that contribute to its pharmacological and nutritional properties. Among the major phytochemicals, triterpenoids, polysaccharides, anthocyanins, and flavonoid phenolic antioxidants stand out for their structural characteristics and biological activities—with these components conserved across key cultivated and wild species of the genus.

Triterpenoids in kiwifruit (*Actinidia* spp.), particularly pentacyclic triterpenes such as ursane-type compounds, are significant constituents with notable bioactivities. For example, 2α,3α,24-trihydroxyurs-12-en-28-oic acid and asiatic acid have been identified in radix *Actinidia* extracts using UPLC-QTOF-MS, highlighting the complexity of triterpenoid structures present in this genus (Chen et al., 2021b). These triterpenoids, including saponin derivatives, hydrophilic acids, and compounds structurally related to artemisinin-like molecules, exhibit a range of biological activities such as anti-inflammatory, antimicrobial, and anticancer effects. The presence of these compounds underpins the traditional medicinal uses of *Actinidia* species in treating tumors and inflammatory diseases (Waswa et al., 2024). For representative species *A. chinensis*, corosolic acid—a triterpenoid isolated from its tissues—has demonstrated potential anti-cancer activity by modulating molecular pathways involved in tumor invasiveness, such as promoting ubiquitin-mediated degradation of AXL and inhibiting the GAS6/AXL/JAK2 signaling axis in glioblastoma cells (Kou et al., 2021). This highlights the mechanistic basis of triterpenoids’ pharmacological effects and their therapeutic potential across *Actinidia* spp.

Polysaccharides in *Actinidia* spp., including dietary fibers and complex carbohydrates, contribute to its nutritional value and functional properties. These polysaccharides—conserved across cultivated and wild *Actinidia* species—have been associated with antioxidative, anti-inflammatory, and immunomodulatory effects. While specific structural characterization of *Actinidia* spp. polysaccharides remains limited, their presence is implicated in the fruit’s ability to promote gut health and modulate metabolic parameters (Wang et al., 2021b). Additionally, polysaccharides extracted from the leaves of representative species *A. chinensis* have been optimized using ultrasound-assisted extraction, yielding proanthocyanidin-rich fractions with significant radical scavenging and cellular antioxidant activities, indicating their potential in food science and drug discovery (Lv et al., 2021).

Anthocyanins and flavonoid phenolic antioxidants constitute another major group of bioactivecompounds in kiwifruit. Flavonoids, including catechins, epicatechins, and quercetin derivatives,have been extensively profiled across various *Actinidia* cultivars and species. For instance, the ‘Sweet Gold’ cultivar (a variety of *A. chinensis*) exhibits high contents of phenolic acids such as chlorogenic acid and flavonols like epigallocatechin, contributing to its superior antioxidant capacity (Jeong et al., 2025). Similarly, red-fleshed kiwifruit cultivars (encompassing *A. chinensis* and related species) contain abundant polyphenols such as chlorogenic acid, neochlorogenic acid, gallic acid, protocatechuic acid, procyanidins, and quercetin glycosides, which correlate with enhanced antioxidant, anti-inflammatory, and α-glucosidase inhibitory activities (Yang et al., 2024). Fermentation of *A. chinensis* kiwifruit juice by lactic acid bacteria further enhances the phenolic content and antioxidant capacity, producing new bioactive phenolics such as protocatechuic acid and catechin, which are positively correlated with improved antioxidant activities (Wang et al., 2022d). These flavonoid phenolics—conserved and varied across *Actinidia* spp.—exert their effects through free radical scavenging, modulation of inflammatory pathways, and potential neuroprotective and anticancer activities (Choma and Olszowy-Tomczyk, 2025).

The antioxidant phytochemicals in kiwifruit (*Actinidia* spp.) are not only limited to flavonoids but also include phenolic acids such as caffeic acid, ferulic acid, syringic acid, and rutin—compounds unambiguously identified in water extracts of radix *Actinidia* (Chen et al., 2021b). These phenolic acids, widely distributed across *Actinidia* species and tissues, contribute to the genus’ overall antioxidant potential, supporting its use in managing oxidative stress-related conditions. Moreover, quinic acid—a metabolite derived from caffeic acid glucosides in representative species *A. chinensis*—has been identified as a key bioactive component capable of crossing the blood-brain barrier and exerting antidepressant effects in preclinical models (Marzo et al., 2025).

**Figure 3:**
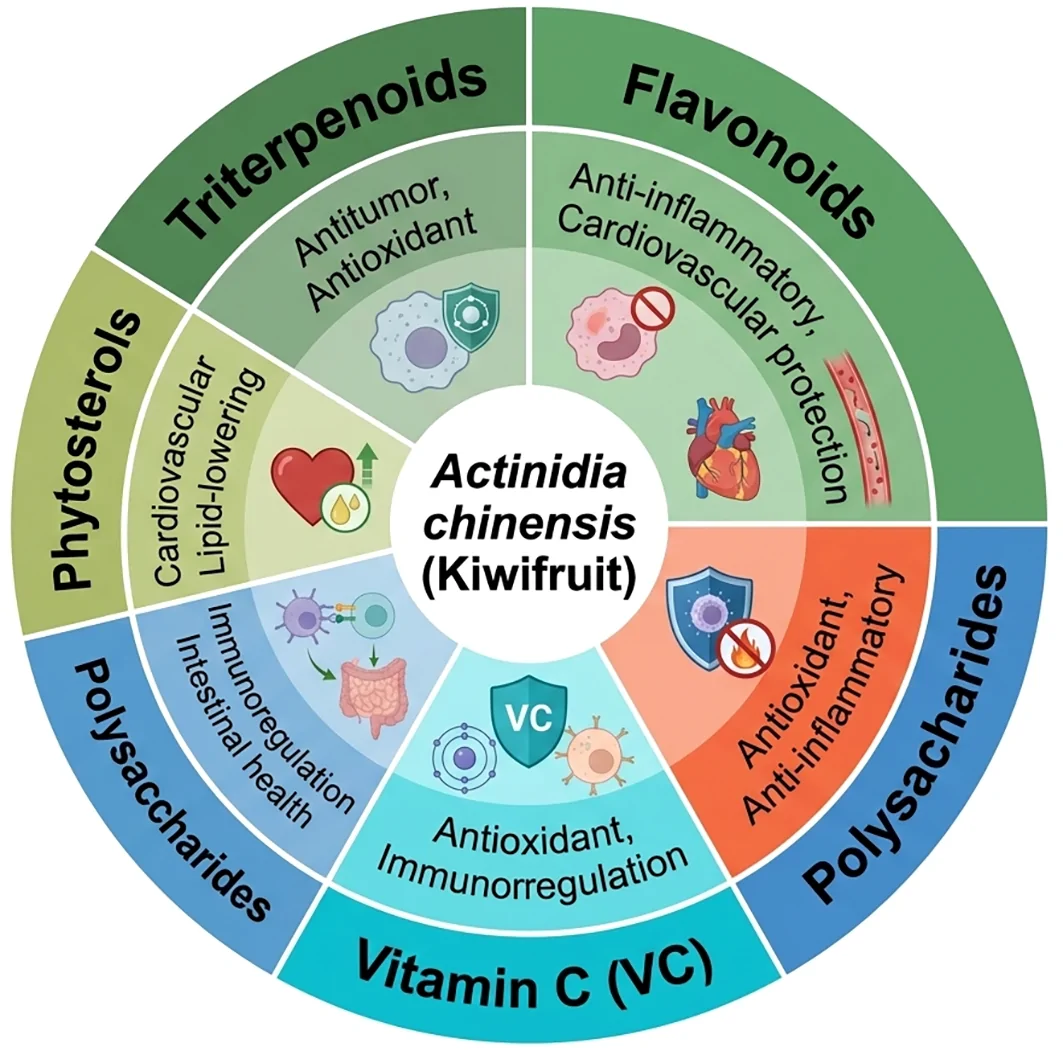
Schematic overview of core bioactive components and corresponding pharmacological activities of *Actinidia chinensis*. This diagram summarizes six predominant functional metabolites in kiwifruit, including triterpenoids, flavonoids, polyphenols, polysaccharides, vitamin C, and phytosterols. Each component contributes distinct health-promoting properties covering antioxidant, anti-inflammatory, antitumor, immunomodulatory, cardioprotective and lipid-regulating effects, collectively underlying the nutritional and functional value of kiwifruit.

In summary, the phytochemical repertoire of *Actinidia* spp. comprises structurally diverse triterpenoids (anti-inflammatory/anticancer), immunomodulatory polysaccharides, and potent flavonoid antioxidants (e.g., catechins, anthocyanins). As illustrated in **Figure 3**, these core components form an integrated functional network underpinning both the nutritional value and pharmacological potential of kiwifruit. While conserved across species, quantitative variations among cultivars drive distinct bioactivities, bridging traditional uses with modern therapeutic applications. Future investigations into structural elucidation, bioavailability, and mechanistic validation are imperative to fully exploit these phytochemicals for health promotion and disease prevention.

The significant inter-cultivar differences in the accumulation of triterpenoids, flavonoids, vitamin C and other bioactive substances provide critical phenotypic indicators for targeted kiwifruit breeding. The subsequent Chapter 6 systematically elaborates the genomic regulatory mechanisms controlling the biosynthesis of these phytochemical metabolites, as well as the application of high-density SNP markers, QTL mapping, genomic selection and marker-assisted breeding in directional improvement of fruit nutritional quality and stress resistance, establishing a complete research chain linking phytochemical phenotypes to molecular genetic improvement.

### 3.2 Unique chemical constituents and their therapeutic potential

Kiwifruit (*Actinidia* spp.) contains a wealth of structurally unique and biologically active secondary metabolites, among which triterpenoids represent the most prominent and extensively studied chemical class. A series of triterpenoids and novel compounds have been isolated and identified from various tissues of *A. chinensis*, with clear CYP450 inhibition, cytotoxicity, antiviral activity, anti-NAFLD effects, and α-glucosidase inhibition. For instance, one new triterpenoid, (2α,3β)- 2,3,23-trihydroxyurs-13(18)-en-28-oic acid (1), together with 12 known compounds (2−13), was obtained from *A. chinensis* roots. Among these isolates, compounds 9−13 exhibited notable cytochrome P450 inhibitory activity, with compound 9 reducing CYP3A4 activity to less than 10% of the control level (Xu et al., 2016). Wei et al. isolated 16 triterpenoids (14−29) from *A. chinensi*s roots, including the new compound 2α,3α,23,24-tetrahydroxyursa-12,20(30)-dien-28-oic acid (14), which showed moderate antitumor activity against HepG2, A-549, MCF-7, SK-OV-3, and HeLa cells, with IC_50_ values ranging from 18.86 to 62.41 µM; its structure was unambiguously confirmed using 1D/2D-NMR and HR-ESI-MS (Wei et al., 2018). Using bioassay-guided isolation, Zhang et al. identified seven compounds (30−36) from the root bark of *A. chinensis*, among which 5-methoxy-coumarin-7-O-β-D-glucoside and spathodic acid-28-O-β-D-glucopyranoside were the main antiviral constituents, with the former showing the highest activity against plant viruses (Zhang et al., 2018b). Li et al. isolated a new ursane-type triterpenoid named actichinone (37), together with 18 known triterpenoids (38−55), from *A. chinensis* roots; these compounds effectively alleviated NAFLD in mice by regulating the AMPK/NF-*κB* signaling pathway (Li et al., 2025a). Nie et al. reported that ethyl acetate and n-butanol fractions of *A. chinensis* root ethanol extracts inhibited α-glucosidase in a concentration-dependent manner, with stronger effects than acarbose. Further UPLC-Triple-TOF/MS analysis identified 21 compounds (56−76) in the ethyl acetate fraction (11 new to *A. chinensis* roots) and 14 compounds (59−61, 77−87) in the n-butanol fraction, including one new natural product (84) and seven compounds isolated from this species for the first time (Nie et al., 2019).

Further phytochemical studies on *A. chinensis* roots have expanded the compound library. Xu et al. identified three new triterpenoids (88−89, 108) and other known compounds (90−115) from roots; compound 88 and its known analogs exhibited significant cytotoxicity against LOVO and HepG2 cells (Xu et al., 2010). He et al. solated eight compounds (116−123) from *A. chinensis* roots, including ursolic acid and daucosterol, as well as three newly identified compounds: 7,4’-dihydroxyflavone, (Z)-9,10,11-trihydroxy-12-octadecenoic acid, and syringaldehyde (He et al., 2016). In another study, He et al. isolated ten compounds (124−133) from *A. chinensis* roots, among which coumalic acid, p-hydroxybenzoic acid, and indole-3-carboxylic acid were obtained from this plant for the first time (He et al., 2015a). From aqueous root extracts, He et al. isolated ten compounds (134−143), including eight new to the genus *Actinidia* and one new to *A. chinensis* (He et al., 2015b). He et al. further purified eight compounds (144−151) from root water extracts, six of which were newly reported in this species (He et al., 2014). Chen et al. isolated 11 compounds (152−162) from *A. chinensis* roots, including the new natural product γ-quinide (152) and three compounds (153, 159, 161) newly reported for this species, which greatly enriched its phytochemical diversity (Chen et al., 2011). Zhou et al. identified eight phenolic compounds (163−170) including tachioside and vanillic acid, as well as six other constituents (171−176), all of which were first reported from *A. chinensis* roots (Zhou et al., 2010). Zhang et al. isolated 20 compounds (177−196) from twigs and leaves, including one novel triterpenoid and 14 compounds first reported in *A. chinensis*, some of which can serve as chemotaxonomic biomarkers (Zhang et al., 2022b). Ma et al. identified four triterpenoids (197−200) including 3β-acetyloxy-ursolic acid from *A. chinensis* fruits for the first time, along with four known compounds (201−204) (Ma et al., 2016). Zhang et al. isolated 12 phenolic compounds (205−216) including protocatechuic acid, ferulic acid, and methyl caffeate from seeds, six of which were newly reported. The active fraction contained 42.46% total phenolic acids, seven times higher than the crude extract, confirming high enrichment efficiency (Zhang et al., 2019b). Bai et al. obtained 12 compounds (217−228) from *A. chinensis* leaves using column chromatography, several of which showed strong antioxidant and free radical-scavenging activities (Bai et al., 2022). Detailed structural information, compound numbering, source plant parts, and biological activities are comprehensively summarized in **Supplementary Table 1**. Corresponding chemical structures of representative bioactive compounds are provided in **Supplementary Figure 1**. After removing duplicates, a total of 173 unique phytochemicals were identified from roots, leaves, fruits, seeds, and twigs of *A. chinensis*. Among these constituents, triterpenoids represent the dominant class (94 compounds, 54.3%), followed by flavonoids (27 compounds, 15.6%) and phenolic acids (21 compounds, 12.1%). Pharmacological activity analysis demonstrated that 21 compounds exerted prominent cytotoxic/antitumor effects against multiple human cancer cell lines, whereas 7 triterpenoid derivatives showed potent lipid-lowering activities. Additionally, abundant flavonoid and phenolic components exhibited outstanding antioxidant and free radical-scavenging capacities.

In addition to triterpenoids, polysaccharides, phenolics, and other small-molecule components also contribute significantly to the bioactivity of kiwifruit. Ding et al. optimized microwave-assisted extraction of polysaccharides from *A. chinensis* roots and obtained a homogeneous galactan (12.713 kDa, 86.53% purity), whose liposomes showed protective effects against acute liver injury in animal models (Ding et al., 2025). Han et al. found that microwave-assisted extraction (MAE) of kiwifruit polysaccharides provided products with lower molecular weight, higher galacturonic acid content, and stronger antioxidant activity than ultrasonic-assisted extraction (UAE) (Han et al., 2019). Zhang et al. purified two water-soluble polysaccharides (ACPS1 and ACPS2) from *A. chinensis* roots using DEAE-52 cellulose and Sephacryl S-300 chromatography. Both polysaccharides showed strong DPPH radical-scavenging activity, protected HEK 293 cells from H_2_O_2_-induced oxidative damage and promoted nitric oxide production and macrophage phagocytosis in a dose-dependent manner (Zhang et al., 2015).

Phenolic compounds represent another major antioxidant group in kiwifruit. Lv et al. optimized ultrasound-assisted extraction of proanthocyanidins from kiwifruit leaves, which showed potent free radical-scavenging activity (Lv et al., 2021). Nie et al. found that chlorogenic acid was the dominant phenolic compound in ‘Donghong’ kiwifruit (*A. chinensis*), peaking at maturity and showing strong pancreatic lipase and α-glucosidase inhibitory activities (Nie et al., 2020).

Actinidin, a characteristic cysteine protease in kiwifruit, also shows important application potential. Kazem and Habeeb optimized actinidin extraction from *A. chinensis* using 6% NaCl and 2% boric acid, achieving a specific activity of 226.6 U/mg after purification. Modern high-resolution metabolomic approaches have greatly advanced phytochemical profiling of *A. chinensis* (Kazem and Habeeb, 2020). UPLC-QTOF-MS analysis of root water extracts identified 295 compounds, including 46 pentacyclic triterpenes, 72 flavonoids, and 53 phenolic acids, among which more than 180 were new to kiwifruit (Chen et al., 2021b). UHPLC-QTOF-MS/MS analysis of roots detected 58 compounds, including 16 triterpenes and 12 flavonoids, with 9 new to the root (e.g., procyanidin B1) (Chen et al., 2020). Collectively, these studies reveal the rich phytochemical diversity of *Actinidia chinensis*, especially its ability to biosynthesize specialized metabolites including triterpenoids and polyphenols, which act as key components of the plant’s biotic and abiotic stress defense systems. Notably, the differential enrichment of vitamin C and various phenolic compounds across kiwifruit tissues endows this crop great potential as a source of functional food ingredients.

The numerous monomer compounds reviewed above can be grouped into four major phytochemical classes, each governed by unique biosynthetic routes and interrelated metabolic regulatory networks. Differences in molecular skeletons, glycosylation and hydroxylation substitutions lead to distinct antioxidant, anti-tumor and lipid-regulating bioactivities, establishing well-defined structure–activity relationships (SAR). Moreover, representative commercial cultivars from New Zealand, Italy, Chile and continental Europe exhibit tissue- and genotype-dependent metabolite accumulation profiles, as reported (Dattola et al., 2024; Richardson et al., 2018). To provide a systematic, balanced global comparison of phytochemical characteristics across major kiwifruit-producing regions, we have added a categorized summary table (**Supplementary Table 2**) to the **supplementary material**. All original tables in the main text remain intact with unchanged numbering to avoid disrupting the manuscript layout.

Nevertheless, most current *in vitro* cell and chemical activity assessments overestimate the practical human health value of kiwifruit phytochemicals, due to prominent gaps between laboratory testing and real human metabolic responses (Richardson et al., 2018). Vitamin C displays favorable gastrointestinal stability and high oral bioavailability after fruit consumption, while most polyphenols undergo severe gastric degradation and limited intestinal absorption; high-molecular-weight dietary fibre cannot enter blood circulation, and its beneficial effects rely entirely on colonic microbial fermentation rather than direct systemic action. Though actinidin retains partial proteolytic activity under gastrointestinal pH ranges, its efficacy is weakened when mixed with daily complex food matrices (Dattola et al., 2024). Existing human intervention trials confirm that long-term kiwi intake relieves constipation and improves serum lipid levels, yet the high compound concentrations applied in *in vitro* assays cannot be replicated through ordinary dietary intake, creating a notable translational barrier for developing oral functional products. Further standardized human pharmacology and clinical research are required to validate the actual *in vivo* efficacy of these phytochemicals.

### 3.3 Extraction and purification techniques of chemical constituents

The extraction and purification of bioactive constituents from *Actinidia* spp. (kiwifruit), particularly A. chinensis, have been substantially advanced by modern green separation technologies. This section systematically compares mainstream extraction and purification approaches across five industrial-oriented dimensions: extraction efficiency, environmental sustainability, cost-effectiveness, technical scalability and industrial applicability, rather than separately describing each technique.

Microwave-assisted extraction (MAE) and ultrasound-assisted extraction (UAE) are the two most widely adopted green techniques. Both deliver short extraction cycles and low organic solvent consumption, yet clear performance gaps exist between them. For polysaccharide recovery, MAE generates fractions with lower molecular weight, higher galacturonic acid levels and stronger antioxidant activity relative to UAE, presenting superior extraction efficiency (Han et al., 2019). Optimized parameters including solvent ratio, temperature and extraction power are critical to preserving the yield and bioactivity of polyphenols, polysaccharides and triterpenoids under microwave treatment (Wang et al., 2022a). In contrast, UAE operates under mild thermal conditions and better protects thermally unstable substances such as seed proteins and proanthocyanidins. Bisht optimized UAE conditions (pH 11.5, 55°C, 4:100 solid-liquid ratio, 100 min) for kiwifruit seed protein, lifting extraction yield by 12% compared with traditional extraction methods; the obtained protein contains abundant glutamic acid, aspartic acid and essential amino acids with excellent emulsifying and gelling properties, suitable as plant-based food additives (Bisht, 2022). Lv et al. further applied UAE to extract leaf proanthocyanidins, and the extracts exerted strong free radical scavenging capacity (Lv et al., 2021). Despite their environmental merits, UAE suffers from longer processing time and lower batch throughput, limiting its large-scale industrial competitiveness against continuous MAE equipment.

Phenolic acids and flavonoids are core antioxidant and anti-inflammatory metabolites of *A. chinensis*, but their polarity and thermal lability demand targeted separation schemes. Electromembrane extraction and thin film microextraction achieve high selectivity and sensitivity for trace phenolic enrichment while cutting solvent usage and pollution, showing outstanding environmental sustainability (Asadi et al., 2021; Ahmadi et al., 2025). Nie et al. confirmed chlorogenic acid as the dominant phenolic constituent in ‘Donghong’ kiwifruit, which accumulates fully at ripeness and strongly inhibits pancreatic lipase and α-glucosidase (Nie et al., 2020). However, microextraction devices are complex to operate and carry low loading capacity, leading to high unit costs and poor scalability; such methods are more fit for laboratory quantitative detection rather than mass industrial production.

Column chromatographic purification represents an irreplaceable tool to obtain high-purity single components, though it falls short in cost and scalability. Ding et al. used MAE to isolate root galactan with purity of 86.53% and molecular weight of 12.713 kDa; liposomal modification of this polysaccharide yielded remarkable anti-oxidative cytoprotective activity, indicating promising functional food applications (Ding et al., 2025). Zhang et al. further separated two homogeneous water-soluble polysaccharides (ACPS1 and ACPS2) via DEAE-52 cellulose and Sephacryl S-300 chromatography. Both polysaccharides eliminate reactive oxygen species, relieve oxidative stress and regulate immune activity in a dose-dependent manner (Zhang et al., 2015). The lengthy elution procedures and expensive chromatographic media raise overall processing costs, making column purification unsuitable for bulk industrial preparation.

For the characteristic cysteine protease actinidin, salt-assisted liquid extraction is the mainstream route. Kazem and Habeeb adopted 6% NaCl combined with 2% boric acid to extract actinidin, reaching a specific activity of 226.6 U/mg post purification (Kazem and Habeeb, 2020). This method is low-cost and easy to operate at lab scale, yet the high salt residue complicates downstream purification and restricts food-grade industrial use.

Novel magnetic nanomaterial-based separation offers unique advantages for trace target enrichment. Agricultural waste-derived Al-Fe_3_O_4_ magnetic nanocomposites realize rapid, selective capture of trace functional metabolites and harmful residues from dried kiwifruit matrices with ultra-low detection limits, supporting raw material quality control and safety testing (Ahmed et al., 2025). Nevertheless, unresolved biosafety risks, nanoparticle residual hazards and difficult material recycling greatly hinder its industrial scalability.

High-resolution metabolomics platforms including UPLC-QTOF-MS and UHPLC-QTOF-MS/MS act as auxiliary analytical tools to quantify extraction performance across different techniques. UPLC-QTOF-MS profiling of root water extracts identified 295 metabolites (46 pentacyclic triterpenes, 72 flavonoids, 53 phenolic acids), over 180 of which were newly reported in kiwifruit (Chen et al., 2021b). UHPLC-QTOF-MS/MS root analysis detected 58 compounds, including 9 tissue-specific newly discovered metabolites such as procyanidin B1 (Chen et al., 2020). These analytical techniques enable quantitative comparison of component recovery among extraction methods, but they cannot serve as preparative separation means for industrial production.

Collectively, green extraction and purification techniques differ substantially in extraction efficiency, operational cost, environmental sustainability and industrial applicability. Microwave-assisted extraction suits high-throughput crude separation with reduced solvent usage, while ultrasound-assisted extraction is optimal for thermosensitive polyphenols and proteins. Microextraction and magnetic enrichment methods are highly sensitive but currently limited to laboratory trace analysis, and column chromatography remains the primary approach for small-scale preparation of high-purity monomers. These technologies provide diversified technical support for efficient separation and high-value utilization of kiwifruit bioactive components.

Current extraction studies still face evident limitations. Most green and nanomaterial-assisted separation approaches remain confined to laboratory exploration, lacking systematic pilot-scale verification and quantitative cross-technique comparison tailored to kiwifruit-specific triterpenoids, polyphenols and polysaccharides. In addition, the biosafety and residual risks of magnetic nanomaterials require further evaluation before large-scale industrial promotion. Future optimization should focus on component-specific parameter tuning, scale-up validation and biosafety assessment, to establish cost-effective and standardized industrial purification systems for kiwifruit functional metabolites.

## 4 Pharmacological mechanisms of kiwifruit

Genetic variation, cultivation conditions and tissue-specific metabolite accumulation shape the differential enrichment of bioactive compounds in kiwifruit, endowing this fruit with multiple pharmacological properties. Existing preclinical evidence demonstrates that kiwifruit-derived metabolites exert antioxidant, anti-inflammatory, metabolic-regulating, cardioprotective and intestinal-modulating activities via regulating core cellular signaling pathways.

**Figure 4:**
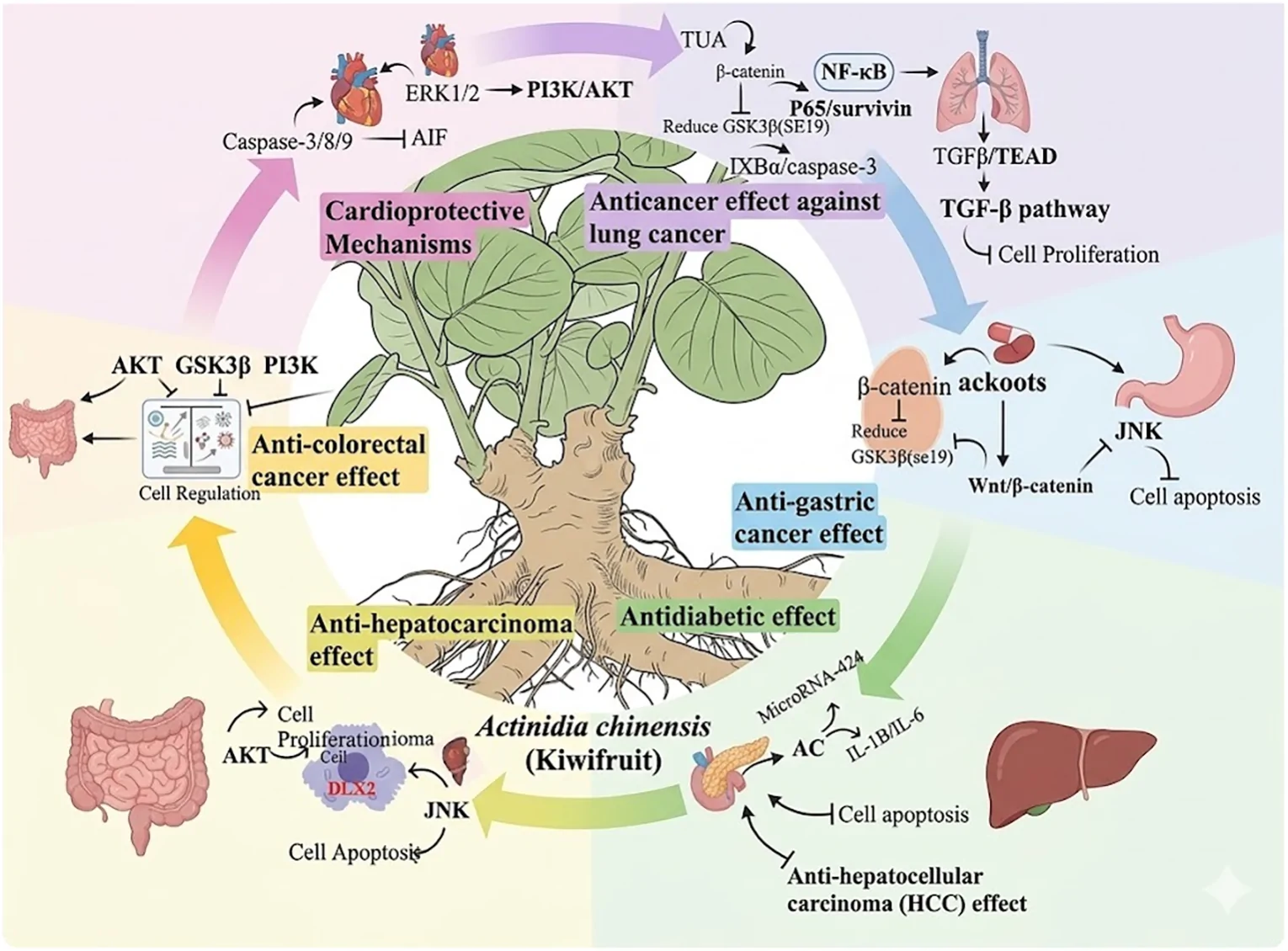
Schematic overview of multi-target pharmacological mechanisms and core signaling pathways of *Actinidia chinensis*. This diagram summarizes preclinical bioactivities of kiwifruit metabolites, including anti-proliferative activity against various tumor cell lines, cardiovascular protection and hypoglycemic effects. These biological functions are mediated by core signaling cascades including PI3K/AKT, NF-κB and Wnt/β-catenin to regulate cell proliferation, apoptosis, inflammatory response and metabolic homeostasis. Notably, these laboratory-based observations lack reliable human clinical trials to confirm therapeutic efficacy in humans.

Notably, most bioactive evaluations are derived from *in vitro* assays and animal models, while human clinical validations remain limited. To date, only intestinal regulatory effects have been consistently verified in human intervention trials. **Figure 4** provides an integrated schematic of multi-target pharmacological actions and core signaling cascades triggered by A. chinensis bioactive metabolites. It summarizes preclinical bioactivities including anti-proliferative effects on multiple tumor cell lines, cardiovascular protection and glucose-lowering capacity, which rely on PI3K/AKT/ERK1/2, Wnt/β-catenin, NF-κB and other pathways to modulate cell apoptosis, inflammatory response and metabolic homeostasis. It is worth emphasizing that these observed *in vitro* and animal effects cannot yet be translated into formal clinical therapeutic applications due to insufficient human trial data.

This chapter systematically summarizes the pharmacological characteristics and molecular mechanisms of kiwifruit functional components, clarifies the current evidence basis of different bioactivities, and provides theoretical support for future directional functional breeding, by-product high-value utilization and development of kiwifruit-derived functional foods.

### 4.1 Antitumor activity and molecular mechanisms

The anti-proliferative effects of *A. chinensis* root extracts are primarily reported in *in vitro* cell assays and preclinical animal models, with no consistent clinical evidence for human tumor intervention. Current *in vitro* studies have demonstrated that acRoots extracts exert concentration-dependent cytotoxicity against hepatocellular carcinoma, gastric cancer, lung cancer, and colorectal cancer cell lines, indicating preliminary tumor-suppressive potential under high-dose laboratory conditions.

*In vitro* cellular experiments have revealed multiple regulatory pathways: acRoots inhibits colorectal cancer cell proliferation, migration, and invasion while inducing apoptosis and cell cycle arrest via suppression of the Notch pathway (Hu et al., 2021). Similar regulatory effects on cyclin D1, survivin, Bcl-2, Bax, and epithelial-mesenchymal transition-related proteins have been observed in *in vitro* breast cancer cell lines, an effect mediated via blocking the AKT/GSK-3β signaling cascade (Gan et al., 2021). In lung cancer cell assays, the cytotoxic response of acRoots correlates with p53 expression status and heat shock protein HSPA6 abundance; network pharmacology further predicted core tumor-related targets such as TP53, AKT1, and TNF (Wang et al., 2020b; Li et al., 2024b). Furthermore, *in vivo* animal experiments verified that acRoots can inhibit tumor angiogenesis in colorectal xenograft models in a dose-dependent manner (Meng et al., 2026).

At the cellular level, the underlying regulatory mechanisms center on the modulation of NF-κB and PI3K/AKT signaling cascades. Root extracts restrain excessive NF-κB activation, downregulate pro-inflammatory mediators, and alter the expression of apoptosis-related proteins in both cell and animal test systems (Saad et al., 2025). Suppression of the PI3K/AKT pathway further limits abnormal tumor cell proliferation and migration under oxidative stress (Gan et al., 2021; Li et al., 2024a). Recent preclinical research also reported that these extracts induce ferroptosis by reshaping intracellular oxidative balance and iron metabolism gene expression, providing a preliminary theoretical basis for developing natural food additives with anti-tumor potential (Meng et al., 2026).

Collectively, *A. chinensis* root extracts serve as multi-pathway regulators of cellular stress responses in preclinical models, yet their therapeutic efficacy against human cancers lacks clinical verification and demands large-scale standardized human intervention trials to confirm translational potential.

Notably, published antitumor assessments of kiwifruit extracts yield highly inconsistent anti-proliferative potency, a discrepancy clearly reflected by the disparate IC_50_ values of triterpenoid constituents compiled in **Supplementary Table 1**. Such divergent readouts arise from three major experimental confounding factors: the use of distinct tumor cell lines with divergent p53 status and endogenous oxidative stress levels, vastly different treatment concentration ranges that far surpass achievable physiological doses via routine fruit consumption, and non-uniform cell incubation periods for cytotoxicity detection. A typical example is that extracts exerting robust suppression on HepG2 hepatocellular carcinoma cells barely inhibit slowly proliferating MCF-7 breast cancer cells under equivalent dosing regimens, which severely limits the universality of antitumor conclusions drawn from separate laboratory investigations.

### 4.2 Antioxidant and anti-inflammatory effects

The antioxidant and anti-inflammatory properties of kiwifruit are mainly validated through *in vitro* chemical tests and animal oxidative/inflammatory models. Polyphenolic components including chlorogenic acid, gallic acid, catechin, and epicatechin enable direct ROS scavenging and upregulation of SOD, CAT, and GSH-PX antioxidant enzymes, effectively alleviating oxidative stress and lipid peroxidation in experimental systems (Aleid et al., 2021; Wu et al., 2023).

In animal models of oxidative stress and gastric mucosal damage, extracts of *A. chinensis* elevate intracellular GSH levels and reduce MDA accumulation, confirming protective antioxidant effects in preclinical settings (Aleid et al., 2021). Notably, immature, thinned kiwifruit by-products contain higher polyphenol concentrations and exhibit stronger *in vitro* antioxidant activity than mature fruits, indicating promising resource utilization value (Deng et al., 2024). For anti-inflammatory function, kiwifruit polyphenols inhibit the NF-κB signaling pathway *in vitro*, reducing the secretion of pro-inflammatory cytokines TNF-α, IL-6, and IL-1β; polyphenol-rich fractions suppress macrophage activation and nitric oxide (NO) generation, while kiwifruit extracts alleviate systemic inflammatory responses in animal models (Wu et al., 2023; Paśko et al., 2024; Peng et al., 2022).

In summary, the antioxidant and anti-inflammatory effects of kiwifruit rely on its polyphenol components and the regulation of oxidative and inflammatory signaling pathways, contributing to gastroprotective and hepatoprotective benefits. The valorization of kiwifruit by-products provides a sustainable strategy for developing natural antioxidant and anti-inflammatory ingredients (Waswa et al., 2024; Wu et al., 2023).

### 4.3 Metabolic regulation and cardiovascular protection

Evidence for metabolic-regulating and cardiovascular-modulating properties of kiwifruit mainly originates from *in vitro* biochemical analysis and high-fat diet rodent models, with limited supporting data from human trials. Phenolic compounds enriched in *A. chinensis* effectively relieve oxidative stress in hyperglycemic and dyslipidemic animal models, improve insulin sensitivity and glucose uptake, and modulate lipid profiles by lowering serum TG, TC, LDL-C and increasing HDL-C levels (Kim et al., 2021; Zhou et al., 2024).

Kiwifruit phenolics can bind to human serum proteins to stabilize conformation and regulate lipid transport (Kim et al., 2021). Fresh kiwifruit extracts inhibit ACE activity, thus helping regulate blood pressure through the renin-angiotensin-aldosterone system (Gu et al., 2024). Meanwhile, kiwifruit peel extract activates the AMPK pathway, promotes fatty acid β-oxidation, and inhibits lipogenesis, thereby alleviating hepatic lipid accumulation (Zhou et al., 2024).

*In vitro* binding assays confirm that kiwifruit phenolics stabilize human serum protein conformation and modulate lipid transport processes. Fresh fruit extracts suppress ACE activity at the molecular level, implying potential mild blood pressure-regulating capacity (Kim et al., 2021; Gu et al., 2024). Kiwifruit peel extracts activate the AMPK pathway, accelerate fatty acid β-oxidation, and inhibit *de novo* lipogenesis, mitigating hepatic lipid deposition in high-fat fed animals (Zhou et al., 2024). Additional studies on *Actinidia* arguta ‘Geneva’ demonstrated that its extracts downregulate cholesterol biosynthesis genes and reduce hepatic lipid accumulation in mice (Gorji et al., 2024). The cysteine protease actinidin from green kiwifruit displays fibrin-degrading activity *in vitro*, suggesting preliminary thrombosis-preventing potential (Pinontoan et al., 2024). Moreover, kiwifruit phenolics inhibit LDL oxidation, protect vascular endothelial cells from oxidative damage in cell models, and theoretically reduce cardiovascular disease risk markers under laboratory conditions (Kim et al., 2021).

Collectively, preclinical data reveal promising metabolic and cardiovascular protective potential of kiwifruit bioactives under experimental conditions, yet these pharmacological outcomes rely heavily on artificially high doses and controlled animal environments. Reliable human clinical validation is still absent, and their actual efficacy in human populations remains to be systematically clarified.

### 4.4 Intestinal health promotion effects

Distinct from the pharmacological activities described above, the intestinal regulatory effects of kiwifruit are supported by both preclinical experimental data and credible human clinical intervention trials. Multiple clinical studies confirm that daily consumption of whole fresh kiwifruit markedly increases defecation frequency, relieves constipation and abdominal discomfort, and reduces circulating TNF-α concentrations in patients with constipation-predominant irritable bowel syndrome (IBS-C), providing robust evidence for its intestinal-beneficial effects in human subjects (Eady et al., 2020).

*In vitro* simulated fermentation and rodent feeding experiments further elucidate the underlying mechanisms: kiwifruit powder boosts butyrate production and enriches butyrate-producing commensal bacteria, which maintain colonic epithelial integrity and intestinal barrier function (Goya-Jorge et al., 2023). Kiwifruit intake also enhances gut microbial diversity and reduces the Firmicutes/Bacteroidetes ratio in rats (Alim et al., 2020). It increases beneficial bacteria such as *Lactobacillus* and *Barnesiella*, while inhibiting pathogenic genera including *Enterococcus*, *Escherichia*, and *Staphylococcus*. Polysaccharides extracted from kiwifruit pomace act as prebiotic substrates to stimulate short-chain fatty acid (SCFA) synthesis, sustaining intestinal barrier and systemic immune homeostasis (Chen et al., 2023a). Dietary fiber and bound phenolics from kiwifruit peel further optimize intestinal microbial composition and enrich beneficial flora (Hou et al., 2025). In addition, microbial fermentation metabolites of kiwifruit activate the AhR signaling pathway in intestinal epithelial cells, reinforcing mucosal barrier function and exerting mild anti-inflammatory effects *in vitro* (Goya-Jorge et al., 2023).

Collectively, kiwifruit promotes intestinal peristalsis, regulates gut microbiota and reinforces intestinal barrier function, making it a promising functional food for gut health. Its intestinal benefits vary sharply by genotype and tissue, providing candidate metabolic markers for germplasm screening. However, rodent preclinical studies report conflicting results regarding the microbiota-modulating effects of kiwifruit polysaccharides. Disparities in polysaccharide molecular weight, oral dosage and baseline animal gut flora cause inconsistent trends in the Firmicutes/Bacteroidetes ratio and short-chain fatty acid yields among independent studies. The absence of unified testing standards impedes precise screening of high-prebiotic germplasm via metabolic biomarkers.

### 4.5 Comprehensive limitations and future perspectives

Current pharmacological studies on kiwifruit bioactives are limited by obvious experimental heterogeneity, conflicting phenotypic results, and prominent translational bottlenecks. First, the lack of unified protocols for cultivar selection, tissue sampling, extraction procedures, detection systems and intervention dosages reduces inter-study repeatability and leads to inconsistent functional conclusions. Second, a critical translational gap exists between preclinical observations and practical human applications. Bioactivities validated *in vitro* and in animal models often cannot be reproduced *in vivo*, mainly due to low oral bioavailability, gastrointestinal degradation of active compounds, and food matrix interference. Third, most mechanistic studies focus on individual signaling pathways, while the synergistic interactions among multiple bioactive components and their integrated regulatory networks remain unclear. Fourth, high-quality clinical evidence is severely insufficient. Reliable human validation is currently restricted to the mild intestinal-modulating effects of kiwifruit consumption, whereas its antitumor, anti-inflammatory and metabolic regulatory potentials lack rigorous clinical support.

To mitigate these limitations, future research should establish standardized pharmacological evaluation criteria and conduct multi-dimensional comparative experiments to resolve existing experimental discrepancies. Expanded, well-controlled human trials are also required to verify the translational efficacy of kiwifruit bioactives. These efforts will support the precise functional characterization and industrial transformation of kiwifruit-derived functional ingredients. In addition, existing findings demonstrate the promising value of kiwifruit processing by-products for high-value utilization, facilitating the sustainable and diversified development of the kiwifruit industry.

## 5 Agricultural applications and cultivation techniques of kiwifruit

In recent years, integrated ecological cultivation, genomic-assisted disease resistance breeding and green postharvest preservation have reshaped the full production system of Actinidia. Consensus has been formed that coordinated field management, targeted Psa prevention and low-loss storage technologies jointly guarantee stable yield and commercial fruit quality, while prominent bottlenecks remain in the popularization of resistant varieties and standardized ecological planting models worldwide.

**Figure 5:**
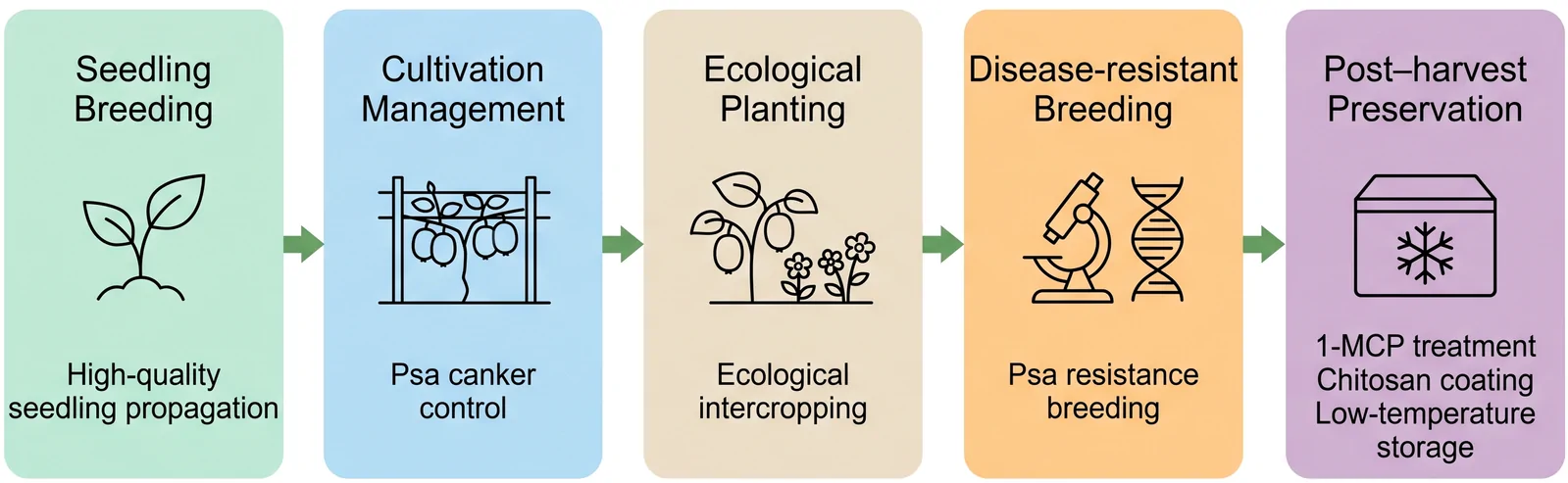
Integrated technical framework for kiwifruit cultivation, pest management and postharvest storage. This flowchart outlines the complete industrial production chain of kiwifruit, and highlights core sustainable horticultural technologies to stabilize fruit yield and maintain postharvest quality throughout the whole production cycle.

**Figure 5** outlines the complete technical framework covering seedling breeding, field management, pest control and postharvest preservation, with key sustainable technologies including bacterial canker intervention, ecological intercropping, ethylene inhibition by 1-MCP, chitosan coating and low-temperature storage highlighted. This chapter integrates progress in propagation optimization, ecological regulation, resistance breeding, fruit quality control and postharvest maintenance, and discusses practical industrial constraints to provide technical references for global high-quality kiwifruit production.

### 5.1 Optimization of propagation techniques

Somatic embryogenesis and *in vitro* propagation are pivotal for the efficient clonal multiplication of kiwifruit (*Actinidia* spp.), including the major cultivated species *A. chinensis* and *A. deliciosa* (Dutta, 2024). These biotechnological approaches address the increasing demand for high-quality, uniform planting materials and underpin modern genetic improvement programs. Recent advances have enabled reliable somatic embryo induction from diverse explants and successful conversion into hardened plantlets under optimized *in vitro* conditions. Propyl gallate (PG) has been shown to modulate callus formation by regulating gene expression related to phytohormone signaling and glutathione metabolism, providing a viable strategy to improve tissue culture efficiency (Li et al., 2024c). These biotechnological tools overcome major limitations of conventional propagation, such as low rooting rates and slow multiplication, which restrict large-scale commercial production. Optimized basal media and plant growth regulator combinations enhance somatic embryo induction and maturation, delivering high regeneration frequencies and phenotypically uniform plantlets. Micropropagation systems using nodal segments and shoot tips enable rapid clonal multiplication while preserving genetic fidelity. Shoot tip culture combined with thermotherapy or cryotherapy effectively eliminates viruses from *A. macrosperma*, reducing losses caused by viral diseases (Zhang et al., 2024). These tissue culture platforms shorten breeding cycles and produce pathogen-free seedlings, which are essential for sustainable kiwifruit production (Zhang et al., 2022a).

In cutting propagation, indole-3-butyric acid (IBA) promotes adventitious root formation, but its use is limited in organic production, driving the development of eco-friendly biostimulants. Seaweed extracts enhance rooting in *A. deliciosa* cuttings by improving morphological traits, upregulating root-related genes (*GH3-3, LBD16, LBD29, LRP1*), and increasing chlorophyll and carbohydrate accumulation. Similarly, seaweed extracts and humic acid improve rooting, root morphology, biomass and pigment content in ‘Monty’, ‘Hayward’ and ‘Allison’ cuttings, with key root-associated genes upregulated (Dutta, 2024), providing a sustainable organic-compliant approach.

Grafting is also widely used in kiwifruit production, and DNA-based markers have been developed to predict graft compatibility and improve graft survival (Ashraf et al., 2025). Furthermore, another culture and gametophytic regeneration systems have been established to produce haploid and doubled haploid plants, which are highly valuable for breeding programs due to complete homozygosity and genetic stability. Despite the complex polyploid nature of *Actinidia*, these techniques enable efficient haploid induction and support germplasm innovation and genetic enhancement (Li et al., 2025b). For conventional seedling production, a patented organic nursery system has been developed for *A. chinensis*, in which mature seeds are sown in sterilized seedbeds amended with composted livestock manure and covered with a sand-sawdust mixture at 10−15 cm within-row and 18−22 cm between-row spacing, yielding high-quality, stress-tolerant seedlings suitable for standardized orchard establishment (Wang, 2015).

To further improve propagation efficiency and breeding precision, molecular tools have been implemented in kiwifruit cultivation systems. A SCAR marker MSY01 (365 bp) has been developed for sex identification in *A. chinensis*, *A. deliciosa*, *A. eriantha*, and *A. macrosperma*, allowing early screening of male and female seedlings and enhancing the efficiency of parental selection and orchard planning (Peng et al., 2023). Meanwhile, REML/BLUP analysis has been used to evaluate narrow-sense heritability (h²) for nine traits across 35 families of *A. chinensis*, with breeding values facilitating the identification of elite parents for fruit weight, flesh firmness, and soluble solids content and supporting marker-assisted breeding strategies (Pinto et al., 2018). Pollination management also constitutes an important component of effective propagation and yield assurance. Artificial pollination has been found to achieve 72−85% fruit set in ‘AU Golden Sunshine’ and ‘AU Gulf Coast Gold’, exceeding the efficiency of insect and open pollination (Abbate et al., 2021). Tetraploid cultivars require fewer pollen grains than hexaploid genotypes, and evening pollination can further improve pollination effectiveness (Broussard et al., 2021). Combining tissue culture, grafting, molecular screening and standardized pollination forms an integrated propagation system for sustainable kiwifruit improvement.

Beyond traditional propagation, optimized Agrobacterium-mediated transformation systems facilitate kiwifruit gene function research. High-efficiency stable transformation via A. tumefaciens GV3101 is available for A. chinensis, whereas A. rhizogenes K599-mediated hairy root transformation and a tissue-culture-free transient transformation protocol support rapid gene validation in the recalcitrant A. valvata. Dual transformation platforms thus enrich technical options for kiwifruit molecular breeding (Xia et al., 2026).

### 5.2 Cultivation management and ecological regulation

Cultivation management and ecological regulation are core to sustaining high yield and superior quality of kiwifruit (*Actinidia* spp.), as they coordinate plant growth, environmental adaptation, and resource allocation. The following sections elaborate on key management strategies and their regulatory effects on kiwifruit growth and quality.

#### 5.2.1 Canopy, pruning, and yield regulation

##### 5.2.1.1 Shoot, branch, and cane management

Shoot and branch traits exert significant impacts on kiwifruit quality. For instance, TE shoots in ‘Gold3’ kiwifruit produced fruits with higher soluble solids content and sweetness, whereas CNT shoots exhibited stronger antioxidant capacity (Dattola et al., 2024). Short shoots in ‘Zes008’ were found to reduce fruit dry matter (DM) and red pigment accumulation, while a fruiting branch diameter of 15−18 mm was identified as optimal for comprehensive fruit quality (Jin et al., 2016). In terms of cane management, maintaining small cane diameters (12.7 mm) in ‘Zesy002’ enhanced yields by 22.7% under high crop loads, while larger canes improved yield stability (Thorp et al., 2021); early terminal flowers in ‘Zesy002’ also yielded heavier fruits with higher DM and sugar: acid ratios (Richardson et al., 2019).

##### 5.2.1.2 Summer pruning optimization

Targeted summer pruning strategies are effective in refining kiwifruit yield and quality.Pre-flowering shoot squeezing-twisting combined with post-fruit set removal of distal vegetative growth increased fruit weight by 20%, ascorbic acid (AsA) content by 19%, and DM by 6.6% in ‘Jinyan’ kiwifruit (Liao et al., 2020), highlighting the importance of timely pruning in regulating resource allocation (Liao et al., 2019b).

#### 5.2.2 Pollination, planting density, and yield stability

##### 5.2.2.1 Female: male ratio and planting density

The optimal female-to-male ratio (≤10:1) and planting density (≤500 plants/ha) minimize inter-plant resource competition, thereby improving yield stability (Meroi Arcerito et al., 2024). Excessive pollen application, however, was shown to reduce average fruit weight, indicating the need for balanced pollination management.

##### 5.2.2.2 Effective pollination period and sex differentiation

The effective pollination period (EPP) for *A. chinensis* ‘AU Golden Sunshine’ and *A. deliciosa* ‘AU Fitzgerald’ ranges from 4 to 7 days after anthesis (DPA), with temperature and alternate bearing being key influencing factors (Brantley et al., 2019). Furthermore, seven developmental stages of sex differentiation have been defined in *A. chinensis*, with stage 3 (3.5−4.5 mm flower length) being critical for carpel arrest in male plants (Caporali et al., 2019), providing a theoretical basis for targeted sex-specific cultivation practices.

#### 5.2.3 Growth regulators and carbohydrate management

##### 5.2.3.1 Application of growth regulators

Growth regulators play a pivotal role in regulating kiwifruit fruit development. The application of CPPU promotes fruit growth through cell expansion and division, upregulates gibberellin and cytokinin signaling pathways, and increases fruit weight and sugar accumulation without altering DM or vitamin C content (Wu et al., 2020; Qiu et al., 2020). Combined application of 14-OH BR (0.15 mg/L) and CPPU (5 mg/L) for ‘Donghong’ kiwifruit achieved yield and quality comparable to 10 mg/L CPPU alone while extending postharvest life (Wang et al., 2022b), and sequential application of CPPU and NAA exerted synergistic effects on fruit weight in *A. deliciosa* (Childerhouse, 2009).

##### 5.2.3.2 Carbohydrate supply regulation

Carbohydrate supply significantly modulates kiwifruit quality. An increased leaf-to-fruit ratio (6:1) and phloem girdling enhanced ‘Zesy002’ fruit weight by 38%, along with starch and sugar accumulation and cytokinin content, accelerating fruit ripening. In contrast, reduced carbohydrate supply in ‘Gold3’ increased citric and quinic acid contents by 18−22% and decreased sucrose by 15%, leading to elevated perceived acidity (Le Lievre et al., 2021) [105].

#### 5.2.4 Environmental and soil factors

##### 5.2.4.1 Altitude and climatic effects

Altitude and climate exert distinct impacts on kiwifruit growth and quality. Higher-altitude, cooler regions in Iran enhanced AsA, phenolics, and anthocyanins in red-fleshed kiwifruit (Asadi et al., 2024a), while lower altitude improved ‘Hongyang’ photosynthetic efficiency, characterized by higher light saturation point (LSP), light compensation point (LCP), maximum net photosynthetic rate (Pmax), and fruit chlorophyll/carotenoid content (Gong et al., 2025). Plastic house cultivation advanced ‘Haegeum’ maturity by 15 days by increasing daily temperature ranges (Cho et al., 2016).

##### 5.3.4.2 Soil nutrients and fertilization

Soil nutrients are key determinants of kiwifruit quality. Soil alkaline hydrolyzable-N and available Fe were positively correlated with ‘Hayward’ fruit weight and soluble solids, while soil pH and available B negatively affected fruit shape (Guo et al., 2020). Soil organic matter was the primary factor influencing ‘Donghong’ fruit quality, with Olsen-K and available S as secondary factors (Chen et al., 2021b). Iron chelate application to high-pH soils improved ‘Hort16A’ chlorophyll content, fruit de-greening, and productivity by 23−56% (Thorp et al., 2021), and slow-release Fe-EDDHSA/CaCO3 mitigated Fe deficiency *in vitro* by promoting Fe release through root-induced pH reduction (Baldi et al., 2019). A balanced N-P-K fertilization regime (560 g N, 250 g P_2_O_5_, 250 g K_2_O per plant) enhanced ‘Hongyang’ photosynthesis and yield (27.7 t/ha) (Li et al., 2015).

##### 5.2.4.3 Specialized fertilizer application

Specialized fertilizers further improve kiwifruit quality and soil conditions. Microbial-fermented bio-organic fertilizer increased soil organic matter and fruit quality (Yan, 2022), and foliar Se application at 10−25 days post-fruiting and pre-maturity enhanced flesh Se content (Shao et al., 2016), providing a basis for nutrient-specific fertilization strategies.

#### 5.2.5 Cultivar characteristics and comparisons

##### 5.2.5.1 Yellow-fleshed vs. green-fleshed cultivars

Cultivar type significantly influences kiwifruit quality. Yellow-fleshed *A. chinensis* cultivars (‘Gold3’, ‘Jintao’) outperformed green-fleshed *A. deliciosa* ‘Hayward’ in fruit weight, soluble solids content (SSC), firmness, and antioxidant capacity (Dattola et al., 2024). The new yellow-fleshed cultivar ‘Heijin’ exhibits excellent agronomic traits, with a fruit weight of 104.83 g, SSC of 20.4%, vitamin C content of 1160 mg·kg^-1^, and a yield of 27,700 kg·hm^-2^ (Liu et al., 2024).

##### 5.2.5.2 Pollen donor effects and cultivation modes

Pollen donor selection impacts fruit quality: pollination with *A. eriantha* ‘WF1’ optimized ‘Ganmi 6’ fruit quality, achieving 100% fruit set and higher sugar and AsA contents (Liao et al., 2022), while pollen from ‘M91’ and ‘M33’ increased ‘Zesy002’ fruit weight via higher seed number, with larger fruits showing higher sugar and lower acid contents (Seal et al., 2016). Ecological green and organic cultivation practices further enhance fruit quality: green practices increased ‘H-1’ soluble solids by 15.9%, sugar by 26.3%, and AsA by 29.25%, while organic cultivation improved essential amino acid content and unique aromas (Zheng et al., 2019). In the Central Himalayan Region, five female cultivars (Abbott, Allison, Bruno, Hayward, Monty) and the male cultivar ‘Tomuri’ showed significant differences in fruit weight (53.10−69.17 g), AsA content (41.3−83.6 mg/100 g FW), and cultivar-specific antioxidant parameters (total phenols, flavonoids, DPPH activity) (Trivedi et al., 2015).

#### 5.2.6 Molecular and metabolic regulation

##### 5.2.6.1 Flavonoid and carotenoid biosynthesis

Molecular and metabolic mechanisms underpin the formation of kiwifruit fruit quality. Flavonoid accumulation in *A. chinensis* involves 301 flavonoids, 84 biosynthesis-related genes (e.g., LAR, DFR), and 2362 transcription factors (e.g., bHLH74, MYB1R1) (Mao et al., 2024). ‘Hongyang’ had higher flavonoid contents than ‘Jintao’, which was associated with the expression of *AcF3H*, *AcDFR*, *AcUFGT*, and the transcription factors MYB10 and bHLH5 (Yu et al., 2020); *AcBCH1/2* regulated the accumulation of β-cryptoxanthin, β-carotene, and zeaxanthin in yellow-fleshed cultivars (Xia et al., 2022). Transcriptomic and metabolic analyses identified 2,565 differentially expressed genes (DEGs) from 52 transcription factor families that explain quality differences between ‘Jindun No.1’ and ‘Hongyang’ (Guo et al., 2024), and the inner red flesh of ‘Hongyang’ contained 211 metabolites with higher abundance (including 69 flavonoids and 53 phenolic acids), with 23 key genes (e.g., *AcCHS*, *AcUFGT*) validated (Mao et al., 2024). Additionally, fruit shading by bagging significantly altered fruit quality traits of *Actinidia eriantha*, accompanied by extensive differentially expressed genes (DEGs) and metabolic changes mainly enriched in ascorbic acid metabolism pathways (Liu et al., 2022a).

##### 5.2.6.2 Aroma and flavor formation

Aroma and sensory traits are cultivar-specific. Natural variation in the *AAT1* promoter in ‘Donghong’ enhanced ester accumulation, driving aroma differences (Cao et al., 2024), and 2-Pentylfuran effectively distinguished green- and yellow-fleshed cultivars, with ‘Dorì’ having the highest ester content, which correlated positively with aroma and sweetness (Cozzolino et al., 2020). Integrative metabolome-transcriptome analysis identified 139 flavonoids in *A. chinensis* flesh, with 76% accumulating significantly at 20 DPA (correlated with downregulated *PAL*); β-cryptoxanthin peaked at 175 days, driven by differential cyclase gene expression in carotenoid pathways, and WGCNA revealed two transcription factors regulating flavonoid and carotenoid biosynthesis and transport, shedding light on the molecular mechanisms of yellow-flesh color formation (Xiong et al., 2023).

#### 5.2.7 Other cultivation-related traits

Other cultivation-related traits also affect kiwifruit production and quality. SSR analysis of 24 tetraploid *Actinidia* populations revealed high genetic diversity, distinct eastern and western clusters, and predicted habitat loss (Wang et al., 2024a). Cane vigour exerted species-specific effects: low-vigour canes reduced fruit size in *A. arguta* but had minimal impacts on *A. chinensis* quality (Medic et al., 2021). Pericarp structure differed between species: *A. chinensis* Planchon softened earlier with slower starch degradation, while *A. chinensis* var. *deliciosa* had thicker cuticles and stone cells (Yang et al., 2015). Nutrient accumulation and partitioning studies showed that yellow-fleshed *A. chinensis* accumulated N, P, and K linearly until harvest, with 1-year-old shoots storing 32 kg/ha N and 23 kg/ha Ca (Quartieri et al., 2022). Pre-flower girdling disrupted ‘Hayward’ xylem pressure recovery and carbohydrate transport, altering resource partitioning and thus plant growth and fruit development (Haisman, 2018).

### 5.3 Disease resistance breeding strategies

Disease resistance breeding is an essential sustainable approach to mitigate the devastating Psa bacterial canker threat to global kiwifruit production. Dissecting resistance genetics, mining wild germplasm resources and utilizing modern genomic tools lay its theoretical foundation. Advances in QTL mapping, pan-genome analysis and functional genomics have uncovered pivotal resistance genes and mechanisms, which greatly accelerate durable resistant cultivar development through MAS and biotechnologies, and underpin the sustainable development of kiwifruit industry (De Mori et al., 2025).

#### 5.3.1 Genetic mapping and QTL analysis

Quantitative trait locus (QTL) mapping is fundamental for dissecting the genetic architecture of complex traits like disease resistance, providing the foundation for marker-assisted selection (MAS).

A high-resolution interspecific genetic map between *A. chinensis* var. *Chinensis* and *A. arguta* was constructed using ddRAD-seq. After artificial inoculation, a major resistance QTL was detected on chromosome 28, and two minor resistance QTLs on chromosomes 4 and 17, while a susceptibility QTL was identified on chromosome 9 in *A. chinensis* var. *Chinensis* (Tahir et al., 2020).

In a tetraploid *A. chinensis* population, identity-by-descent (IBD) mapping revealed multiple loci associated with Psa resistance, including receptor-like kinases (*RLKs*) involved in defense (Flay et al., 2024). Tetraploid induction via colchicine has also been shown to enhance the expression of stress resistance-related differentially expressed genes (*DEGs*), suggesting a ploidy-based strategy for resistance breeding (Li et al., 2019).

A modified bulk segregant analysis (*BSA*) applied in a diploid *A. chinensis* population identified 11 QTLs related to Psa resistance, offering an efficient method for marker development (Flay et al., 2024).

Beyond Psa, a 5.73 Mb locus on chromosome 10 confers resistance to the scale insect *Hemiberlesia lataniae* (Flay et al., 2023), demonstrating the application of mapping to other pests.

#### 5.3.2 Screening and characterization of resistance resources

##### 5.3.2.1 Wild germplasm and cultivar evaluation

Wild *Actinidia* species serve as vital reservoirs of resistance genes. Screening of 104 wild genotypes (30 species) revealed significant variation in Psa tolerance/resistance (Wang et al., 2020a). Cultivar screenings have identified promising materials; for instance, 38.71% of 62 tested cultivars showed Psa resistance, correlated with high peroxidase (*POD*) and catalase (*CAT*) activities (Wang et al., 2023a). Specific examples include *A. arguta* ‘Hortgem Tahi’, which shows high Psa resistance but susceptibility to sclerotinia, and *A. chinensis* ‘Zesy002’, which demonstrates broad-spectrum resistance (Reglinski et al., 2023). Comparative studies indicate *A. arguta* exhibits higher Psa tolerance than *A. chinensis* through early pathogen recognition (e.g., *Pto3* expression), rapid antioxidant activation, and suppressed ethylene/JA pathways (Nunes da Silva et al., 2020).

##### 5.3.2.2 Resistance mechanisms and biomarkers

Transcriptomic studies comparing resistant and susceptible cultivars provide insights into defense mechanisms. Analysis of resistant ‘Jinyan’ vs. susceptible ‘Hongyang’ after *B. dothidea* infection identified 13,898 *DEG*s, with 2,373 defense genes enriched in calcium signaling and cell wall modification pathways (Wang et al., 2020d). Specific resistance genes identified include *PR1* and *FLS2*, while transcription factors *AcREM14* and *AcC3H1* upregulate salicylic acid (SA) signaling (Kuang et al., 2024; Zhao et al., 2024b). Metabolically, flavonoid biosynthesis genes *Ac4CL1, Ac4CL3*, and *AcHCT1* differentially regulate Psa immunity, with *Ac4CL1/AcHCT1* promoting defense and *Ac4CL3* inhibiting it (Ma et al., 2025a). Physiological biomarkers are also valuable; leaf spongy tissue thickness negatively correlates with Psa resistance in hybrids (He et al., 2024).

##### 5.3.2.3 Innate immunity in wild species

Wild species exhibit robust innate immunity. *Actinidia melanandra* recognizes specific Psa3 effectors (e.g., *HopAW1a*) to trigger effector-triggered immunity (*ETI*), while its PAMP-triggered immunity (*PTI*) relies on recognition of the type III secretion system (T3SS), making it a source of durable resistance genes (Hemara et al., 2025). Research on pathogen effectors is crucial; the loss of certain Psa3 effectors (e.g., *AvrRpm1a*) enhances virulence in *A. arguta*, whereas the wild-type effector triggers a hypersensitive response, highlighting the role of specific effector recognition in resistance (Hemara et al., 2022).

#### 5.3.3 Genomic resources, functional genomics, and editing

##### 5.3.3.1 Pan-genome and resistance gene analogs

The construction of a super pan-genome from high-quality genome assemblies of multiple *Actinidia* species reveals extensive structural variations and resistance gene analogs (*RGAs*), providing a comprehensive resource for identifying novel resistance loci and supporting precise breeding. For instance, Yu et al. established a kiwifruit super pan-genome using 15 high-quality assemblies from eight species, systematically identifying *RGAs* and generating a pan-RGA dataset to clarify the diversity of disease resistance genes in the genus (Yu et al., 2025). Wu et al. further leveraged a graph-based pan-genome across wild and cultivated species to uncover extensive structural variations, identifying a wild-specific *NLR25–1* allele. This allele enhances defense against *Pseudomonas syringae* via a transposable element-induced promoter variation, illustrating how pan-genome-enabled mining of wild relatives facilitates the discovery of valuable resistance genes for kiwifruit improvement (Wu et al., 2026).

##### 5.3.3.2 Functional characterization of defense genes

Systematic characterization of gene families identifies key immune regulators. For example, the *LRR-RLK* gene family in ‘Hongyang’ includes 394 non-redundant genes, with transcriptomic profiling identifying 48 core *LRR-RLK* genes induced by Psa, especially in subgroup XII, indicating essential roles in defense signaling. *CRISPR-Cas9* was used to validate the function of *AcJAZ2L2*, a repressor in the jasmonate signaling pathway that regulates stomatal immunity and confers resistance to bacterial canker. Overexpression reduced symptoms, while knockout increased susceptibility, confirming the potential of genome editing (Wang et al., 2025).

#### 5.3.4 Integrated strategies and molecular regulation of resistance

##### 5.3.4.1 Grafting and rootstock utilization

Grafting susceptible scions onto resistant rootstocks provides a rapid management strategy. Grafting ‘Hongyang’ onto *A. guilinensis* ‘Gui-1’ rootstock improves Psa resistance (Wang et al., 2021a).

##### 5.3.4.2 Induced resistance and biocontrol

Chemical elicitors and biocontrol agents can prime or enhance plant defense. Actigard™ induces pathogenesis-related genes (*PR1/PR2*) to limit Psa (Stroud et al., 2022). Saccharin, UV-C irradiation, fosetyl-aluminum, and BABA (β-aminobutyric acid) have all been shown to induce resistance responses against Psa (Reglinski et al., 2024; Lucioli et al., 2024; Brunetti et al., 2020). Biocontrol agents like *Aureobasidium pullulans* YBCA5 (inhibits Psa via stomatal colonization) and *Pseudomonas fluorescens* isolates from floral microbiomes also reduce disease incidence (Rheinländer et al., 2021; Donati et al., 2018).

##### 5.3.4.3 Environmental and hormonal modulation

Environmental factors and hormones finely tune defense. Red LED light enhances constitutive resistance to Psa3 in ‘Hayward’ via higher phenolic content, while blue light reduces symptoms (Correia et al., 2022; Reglinski et al., 2022). Hormone treatments like methyl jasmonate (MeJA) suppress *B. dothideavia* phenylpropanoid enzymes, and IAA enhances resistance to *Botrytis cinerea* (Li et al., 2022, 2023). Nitrate-fed plants show reduced Psa symptoms compared to ammonium-treated plants, indicating a role for nitrogen source (Da Silva et al., 2022). MicroRNAs, such as *miR858*, are key regulators of secondary metabolism, targeting MYB transcription factors to modulate defense-related flavonoid accumulation (Wang et al., 2024b).

##### 5.3.4.4 Understanding pathogen diversity

Effective breeding requires awareness of pathogen evolution. The emergence of novel Psa lineages (e.g., the Psa5 group in Japan) and high heterogeneity within populations (e.g., in Portugal) underscore the need for durable, broad-spectrum resistance (Sawada et al., 2014; Figueira et al., 2020). Studies on low-pathogenic strains reveal a 596 bp interruption in the *HrpR* gene (Zhou et al., 2022).

Taken together, multi-disciplinary strategies including genomic breeding, introgression of wild resistance, molecular marker-assisted selection and integrated pest management are essential for durable and sustainable kiwifruit canker resistance in the future.

### 5.4 Fruit quality formation and quality molecular breeding

#### 5.4.1 Dynamic changes of fruit development and postharvest quality

Fruit development and quality formation of kiwifruit are coordinately regulated by internal physiological metabolism and external environmental conditions. The fruit growth kinetics of *Actinidia chinensis* ‘Hort16A’ exhibits two rapid expansion stages at 30−44 days post anthesis (DPA) and 58−72 DPA. During fruit development, soluble sugar content gradually accumulates, while titratable acid content fluctuates dynamically (Zhang et al., 2017). For *A. chinensis* ‘Allison’, ascorbic acid (AsA) accumulates to the highest level at the early growth stage and decreases at maturity, whereas carotenoids and antioxidant substances continuously accumulate at the late developmental stage, accompanied by increases in fruit size and sugar content (Attri et al., 2016). In red-fleshed kiwifruit, carbohydrate supply significantly regulates flesh anthocyanin accumulation and dry matter (DM) formation. Vine vigor, which is determined by rootstock type and shoot growth status, further affects fruit coloration, and small-diameter bearing canes contribute to enhanced red color intensity of flesh (Nardozza et al., 2017). *In Actinidia eriantha* ‘Ganlv 1’, fruit sweetness is mainly controlled by the starch metabolism balance, in which starch synthesis-related *AGPS* and starch degradation-related *BAM* genes synergistically modulate sugar accumulation characteristics (Li et al., 2024d).

Harvest time and storage performance determine the final commercial quality of kiwifruit. Dry matter is the core evaluation index for ‘Jintao’ kiwifruit and is significantly correlated with soluble solids content (SSC) and sugar–acid ratio (Chen et al., 2019). The harvest period directly determines storage performance: ‘AU Golden Dragon’ harvested in late August with 7.8−8.0% SSC shows the longest storage life, while ‘AU Golden Sunshine’ achieves better postharvest quality when harvested in mid-September (Xie, 2017). Under low-temperature storage conditions, ‘Hort16A’ exhibits superior quality parameters, with SSC of 16.2%, sugar–acid ratio of 18.5, and dry matter content of 19.3%, which are higher than those of ‘AU Golden Sunshine’. In addition, small-sized fruits of all cultivars maintain firmness above 0.5 kgf for 14 weeks during cold storage, whereas large fruits lose flesh firmness 3−4 weeks earlier (Stroman, 2011). Principal component analysis (PCA) was applied to evaluate the fruit quality of ‘Jinyan’ kiwifruit from 53 orchards in Sichuan. The first six principal components explained 88.57% of the total quality variation, and orchards DJY1, GY4, and GY1 showed the best comprehensive quality performance, verifying that PCA is an effective tool for quality evaluation and cultivation optimization (Chen et al., 2021a).

#### 5.4.2 Molecular regulatory network of flavor and nutritional quality

Multi-omics integration helps dissect flavor regulatory networks in kiwifruit. By co-analyzing transcriptomic and metabolomic data from *Actinidia chinensis* ‘Hongyang’, Wang et al. identified key structural genes and transcription factors governing soluble sugars, organic acids, and volatile accumulation, and proposed a gene-metabolite regulatory framework to guide taste- and nutrition-oriented breeding (Wang et al., 2022b).

Sulfur metabolism is critical for the synthesis of ethylene-dependent volatile aromas in kiwifruit. Dynamic headspace sampling (DHS) is more sensitive than solid-phase microextraction (SPME) for detecting methylsulfanyl (MeS) volatiles. In ripening ‘Hort16A’ fruit, MeS volatile accumulation relies on ethylene regulation, and over 98%–100% of these characteristic aromas are lost during long-term cold storage. The expression of alcohol acyltransferase (AAT) correlates with the production of MeS-alkanoate esters, and exogenous ethylene can recover aroma synthesis after storage-induced degradation. Different kiwifruit genotypes share similar AAT substrate preferences, wherein precursor availability limits ester formation. AAT isoforms specific to benzoyl-CoA primarily determine the genotypic specificity of MeS volatile profiles (Günther, 2016).

*AcGLK1*, a GARP transcription factor, serves as a key regulator of chloroplast development and nutrient accumulation in kiwifruit. It promotes chloroplast division by activating *AcFtsZ1*. Heterologous overexpression of *AcGLK1* in tomato increases chlorophyll content by 62% and photosynthetic efficiency by 35%, indicating that *AcGLK1* is a valuable target gene for improving nutrient accumulation and stress tolerance in kiwifruit molecular breeding (Fang et al., 2025).

#### 5.4.3 Conventional breeding and germplasm innovation for high-quality kiwifruit

Conventional cross-breeding continues to generate tangible advances for high-quality, yellow-fleshed kiwifruit. Asadi et al. initiated a systematic breeding program in 2016 and obtained 735 hybrid progenies from controlled crosses. After three rounds of phenotypic selection, 30 superior hybrids with fruit weight >90 g were shortlisted, two of which displayed distinctly novel flesh-texture types and are undergoing further evaluation for yield and disease resistance (Asadi et al., 2024b).

In parallel, the mid-maturing tetraploid cultivar ‘GJ 3’ was released from the cross ‘Jinfeng’× ‘Ganxiong 1’. ‘GJ 3’ shows golden flesh and dry matter (DM) of 19.2–21.2%—approximately 45% higher than that of its female parent ‘Jinfeng’ (13.2%)—and matures roughly one month earlier, offering valuable germplasm for yellow-fleshed kiwifruit improvement (Liao et al., 2019a).

### 5.5 Postharvest preservation technologies

Kiwifruit postharvest quality preservation is critical for its commercial value and consumer satisfaction, as postharvest losses are caused by physiological senescence, microbial decay, and physical damage, affected by pre-harvest conditions, harvest maturity, and storage protocols. Beyond basic cold storage, modern preservation relies on integrated physical, chemical, and biological technologies to maintain fruit firmness, flavor, nutrition, and appearance. This section synthesizes the latest advancements in kiwifruit postharvest science, highlighting key technologies and their efficacy.

#### 5.5.1 Core chemical and physical preservation technologies

1-Methylcyclopropene (1-MCP) is a highly effective ethylene inhibitor widely used to prolong the shelf life of kiwifruit (*Actinidia chinensis*). By competitively binding to ethylene receptors, 1-MCP blocks ripening signals and suppresses downstream processes including fruit softening, chlorophyll degradation, and respiration. In kiwifruit, 1-MCP treatment inhibits the expression of *AcNAC2* and *AcSGR1/2*, thereby retarding degreening and maintaining peel color (Wu et al., 2025b). It reduces ethylene production and respiration, retains flesh firmness and vitamin C, and lowers decay. 1-MCP efficacy depends on harvest maturity, dosage and storage temperature; optimized protocols are critical for commercial use. For ‘Rainbow Red’ kiwifruit, mid-September harvest and 2-day precooling below 10°C before 1-MCP treatment maintained high firmness for 4 months, and post-storage ethylene induced ripening even in non-ethylene-producing fruits (Murakami et al., 2016). Additionally, 1 µL·L^-1^ 1-MCP delayed ripening in red-fleshed *A. chinensis* stored at 0−20°C, reducing ethylene production, respiration rate and weight loss, while preserving firmness; storage at 0°C further extended shelf life without causing chilling injury (Kwanhong et al., 2017).

Chitosan, a natural polysaccharide derived from chitin, is widely used as an edible coating for kiwifruit due to its biocompatibility, biodegradability, and antimicrobial properties. It forms a semi-permeable film to block gas exchange, reduce oxygen uptake and ethylene diffusion, thereby slowing respiration and ripening, maintaining firmness, reducing weight loss, and preserving sensory qualities. Its intrinsic antifungal activity also suppresses postharvest pathogens and decay. Studies show chitosan coatings effectively maintain chlorophyll and delay ascorbic acid degradation, preserving kiwifruit’s visual and nutritional qualities during storage (Hashemi and Jafarpour, 2021). Specifically, chitosan-salicylic acid (CS+SA) composite coating performed better than single treatments, reducing weight loss to 2.60%, preserving vitamin C content at 97.93 mg/kg, and delaying soluble solid content (SSC) degradation during 14-day room-temperature storage (Huang et al., 2017). Besides, the combination of 80 mg/L ClO_2_ and 20 g/L carboxymethyl cellulose (*CMC*) also showed superior performance in inhibiting respiration, reducing weight loss, and maintaining firmness and ascorbic acid (AsA) content compared to single treatments (Zhang et al., 2019a).

Cold storage (0−4 °C) is a fundamental postharvest strategy that suppresses enzymatic activities and slows ripening and senescence. Low temperature effectively delays chlorophyll breakdown and starch degradation, preserving fruit firmness and flavor. The combined use of chitosan coating and cold storage synergistically maintains quality, suppresses microbial spoilage, and retains phenolic and flavonoid compounds (Sicari et al., 2024). Cold storage also downregulates *AcBAM* genes related to starch degradation and the *AcNAC2* transcription factor, further delaying ripening (Wu et al., 2025b; Gong et al., 2025). Gaseous ozone treatment combined with cold storage can further optimize preservation effects: post gradual cooling, ozone treatment enhanced the activities of superoxide dismutase and catalase in ‘Soreli’ kiwifruit, preserved membrane integrity by inhibiting lipoxygenase activity and malondialdehyde accumulation, and improved the efficiency of the antioxidative system and storability during cold storage (Goffi et al., 2020). Similarly, 300 ppb gaseous ozone treatment combined with 2°C storage delayed microbial growth, improved microbial quality index, and enhanced soluble solids content in ‘Soreli’ kiwifruit during 60-day storage, while no quality-preserving benefits were observed when stored at 4°C under the same ozone concentration (Goffi et al., 2019). For red-fleshed kiwifruit cultivars, storage at 0°C vs. 2°C for 4 months showed that 2°C accelerated water loss; firmness declined faster at 0°C initially but equilibrated over time, with no significant differences in SSC, acidity, or dry matter (DM) content (Guerreiro et al., 2022). Cold storage for 30 days also optimized the accumulation of water-soluble vitamins in seven kiwifruit cultivars: vitamin C (AsA) increased over time, while B vitamins showed cultivar-specific trends, such as ‘Qihong’ accumulating B12, and ‘Huayou’ and ‘Hongyang’ having high levels of B3, B7, B9, and C at edibility (Zhu et al., 2016). Additionally, a 3°C, 18-day cold treatment could eradicate *Ceratitis capitata* in kiwifruit, with third-instar larvae being the most tolerant (Lethal Time for 99.99% Mortality, *LT99.99* = 17.3 days); larvae in ‘Zesy002’ (*A. chinensis*) were more susceptible than those in ‘Hayward’ (*A. deliciosa*) (Brown et al., 2024). However, cold storage also has potential risks: early-harvested ‘Dori’ kiwifruit (hue 107°) degreened optimally at 15°C (reaching commercial yellow, 99−100° in 14 days), while storage at 0°C induced 40−50% chilling injury, which could be mitigated at 5°C and eliminated at 15°C (Zoffolia et al., 2024).

#### 5.5.2 Auxiliary postharvest treatments

In addition to the core preservation technologies mentioned above, various other postharvest treatments and management measures have been developed to improve kiwifruit storage quality, which can be categorized into three functional clusters based on their working mechanisms.

Physical interventions including hot-water conditioning and controlled drying modulate fruit respiration and cellular membrane integrity to slow deterioration. A 10 min hot water immersion at 35°C restrains respiratory metabolism, limits starch degradation and membrane leakage, lowers MDA and soluble pectin concentration, and suppresses cellulase and *POD* activities to slow softening and retain vitamin C (Pang et al., 2015); compared with conventional air drying, *EHD* drying at 375 V·mm^-1^ cuts moisture content to one-third of the control within seven hours, and moderate-temperature drying at 40°C for 30 h better preserves total phenolics, flavonoids and ascorbic acid in ‘Sungold’ slices relative to 55°C high-temperature drying (Sicari et al., 2024).

Chemical elicitors represented by EFF compound elevate endogenous antioxidant capacity and restrain oxidative spoilage: formulated with hexanal, geraniol, α-tocopherol and ascorbic acid, 0.02% EFF suppresses oxidase activity and raises fruit phenolic content to 1.22 mg GAE/g FW and FRAP value to 0.96 µmol Fe(II)/g FW (Mthembu et al., 2024), whereas appropriate harvest maturity is also a preliminary chemical-related regulation, as ‘Soreli’ harvested at 8.5°Brix maintains consistently higher postharvest SSC than fruit picked at alternative ripening stages (Cipriani et al., 2018).

Irradiation serves as a mild non-thermal sterilization strategy to sustain original flavor and soluble solids, and dosage control is decisive for its efficiency: irradiation below 800 Gy effectively preserves sensory attributes of ‘Hayward’ and ‘Zesy002’, with 400−800 Gy being the optimal range to minimize flesh softening of ‘Zesy002’ (Sun et al., 2023).

#### 5.5.3 Biological control and pest management

Biological control technologies also play an important role in kiwifruit postharvest preservation: *Trichoderma harzianum* Th1 inhibited Cadophora luteo-olivacea by 70.6−90%, and Brassica nigra biofumigation reduced skin pitting disorders in stored kiwifruit (Di Francesco et al., 2021).

Beyond microbial biocontrol, physical damage during handling and arthropod pests during cold storage constitute two additional loss pathways—each requiring distinct but complementary management strategies. A finite element model (*FEM*) was developed to predict mechanical bruising with ≤18.3% error, confirming that fruit maturity and dropping impact are dominant contributors to postharvest physical injury (Du et al., 2019). Insect pest management during cold storage is another key module: adult White Peach Scale (WPS, *Pseudaulacaspis pentagona*) feeding on cold-stored ‘Hayward’ at 0−1°C requires 203 days (95% CI: 199−208 d) to reach 99% mortality; over 90% individuals survive conventional 60–130 d commercial cold storage, and viable eggs and newly hatched nymphs still emerge after 20°C incubation, indicating surviving pests retain reproductive competence after refrigeration (Stannard et al., 2019).

In addition, recent research newly identified Fusarium fujikuroi as a causal pathogen triggering kiwifruit postharvest rot (Pan et al., 2024), and trumpet atomizer application attained 94–100% control efficacy against brown marmorated stink bug (*Halyomorpha halys*) (Landi et al., 2024).

#### 5.5.4 Non-destructive quality detection and diagnosis

Non-destructive detection and diagnosis technologies provide effective tools for monitoring kiwifruit quality during storage. Optical Coherence Tomography (OCT) at 820 nm (5.5 µm resolution) could visualize subsurface structural changes during shrivel in *A. chinensis*; storage at 1−10°C induced shrivel in ‘Gold3’ and ‘Gold9’ cultivars, with OCT revealing compacted sub-peridermal layers in severely shriveled fruit compared to uniform architecture in healthy tissue (Longuet-Higgins et al., 2024). Visible/near-infrared (*Vis/NIR*) interactance spectroscopy correlated well with SSC in ‘Jintao’ kiwifruit (moderate correlation for firmness, no correlation for acidity, pH, or DM) (Vieira et al., 2018), while Vis/NIR transmittance could predict firmness, SSC, and DM in ‘Hayward’ with >85% accuracy for SSC (Guerreiro et al., 2018). A 433 MHz Surface Acoustic Wave Resonator (*SAWR*) + electronic nose (*EN*) system could discriminate storage time via PCA/SR (Stepwise Regression), with R = 0.99014 for SAWR frequency analysis; the hybrid model outperformed single modalities for weight loss and sensory quality prediction (Liu and Hui, 2015). The key features of these three non‑destructive detection techniques are summarized in **Table 1**.

**Table 1:** Comparison of three non-destructive detection technologies for postharvest kiwifruit quality.

Technology	Principle & key parameters	Cultivar	Target indicators	Performance	Advantages	Limitations	Reference
OCT	820 nm, 5.5 µm resolution; sub-surface imaging	‘Gold3’, ‘Gold9’	Periderm thickness, cell morphology, shrivel degree	Discriminates healthy (uniform) vs. shriveled (compacted) tissues	Real-time, in-vivo, high-res structural analysis	Limited to near-surface layers; no direct biochemical data (e.g., SSC)	Longuet-Higgins et al., 2024
Vis/NIR interactance	Diffuse reflectance spectroscopy	‘Jintao’	SSC, Firmness, TA, pH, DM	Strong SSC correlation; moderate for firmness; no correlation for acidity/pH/DM	Rapid, high-throughput screening	Poor prediction for acidity and DM due to shallow light penetration	Vieira et al., 2018
Vis/NIR transmittance	Transmission spectroscopy	‘Hayward’	SSC, Firmness, DM	SSC accuracy >85%; effective for firmness & DM	Superior internal nutrient profiling vs. interactance	No data on acidity; accuracy varies by cultivar	Guerreiro et al., 2018
SAWR + EN hybrid	433 MHz SAWR + MOS E-nose; PCA/SR modeling	*A. chinensis*	Storage time, Weight loss, Sensory quality	SAWR R^2^ =0.981; EN R^2^ =0.939; Hybrid R^2^ =0.996	Integrates physicochemical & aroma data; higher precision	Validated only for short-term (12 d) ambient storage	Liu and Hui, 2015

#### 5.5.5 Postharvest quality dynamics and pre-harvest regulation

Postharvest degreening kinetics and pericarp structure also affect kiwifruit storage quality. Temperature-dependent chlorophyll degradation in ‘Zesy003’, ‘Zesh004’, and ‘Hayward’ showed that ‘Zesy003’ (yellow-fleshed) degreened fastest at 1−15°C, with higher *PAO1* and *SGR2* expression correlating with color change; cold temperatures downregulated SGR2 (Gambi et al., 2018). ‘Kiss’ kiwifruit stored at 15°C accelerated chlorophyll loss (from 6.8 to 1.2 mg/g FW in 14 days) with carotenoid accumulation, which was linked to enhanced chlorophyllase and magnesium dechelatase activities; 30°C pretreatment followed by 15°C storage optimized degreening (Labra Cordero, 2022). Pericarp structure differences between kiwifruit species also influence storage performance: post-ripening, *A. chinensis* Planchon softened faster but had slower starch degradation compared to *A. chinensis* var. *deliciosa*; the latter had thicker cuticles, abundant trichomes, and cracks, while the former had lenticels, glabrous surfaces, thin cuticles, and exocarp stone cells with slowly degrading, lignified walls (Yang et al., 2015). Specific cultivars showed different quality dynamics during storage: ‘Hayward’ stored for 20−30 days showed optimal quality, with the highest acidity, balanced firmness, enriched fiber, sugars, and organic acids, as well as peak phenolics (15.2 mg GAE/g), flavonoids (8.7 mg RE/g), and actinidin (12.3 U/mg) (Nam, 2016); ‘Gold’ kiwifruit stored at 2°C delayed firmness loss (3.2-fold compared to room temperature) and SSC accumulation (16.8°Brix vs. 21.3°Brix at 20°C), with SSC/TA ratio increasing from 18 to 27 in 5 weeks (Yang and Lim, 2017); ‘Jinmei’ (yellow-fleshed) kiwifruit from Wuhan, Huayuan, and Dafang showed firmness decline to 1.0 kg/cm^2^ after 12-week storage at 1-2°C, with SSC and sugars stabilizing, and Dafang-sourced fruits having lower SSC but higher decay rate (Huang et al., 2020).

Pre-harvest hormone regulation also affects kiwifruit postharvest quality by modulating on-vine ripening. Ethephon (250 µL·L^-1^) upregulated cell wall degradation (*AcPG, AcEXP*), sugar metabolism (*Acβ-AM, AcSUSY*), and ethylene biosynthesis (*AcSEP4/RIN, AcTDR4/FUL*) genes in ‘Kohi’ kiwifruit, promoting ripening (edible 9 days after treatment, 4% drop rate), while ABA had no significant effect (Ampa et al., 2017). Ethephon application at 155 days after full bloom (*DAFB*) reduced firmness, increased SSC, and upregulated *AcACS1/AcACO1*, while ABA enhanced *AcNCED1* expression but did not promote ripening (Ampa et al., 2016). Additionally, kiwifruit peel has outstanding resource- exploiting potential: *A. chinensis* peel accumulates higher polyphenols (12.8 mg/g) and flavonoids (2.7 mg/g) than fruit flesh and exhibits stronger antioxidant and antimicrobial activities, supporting its valorization as a source of natural preservatives and functional ingredients (Alim et al., 2019).

Overall, the integrated application of 1-MCP, chitosan coating, and low-temperature storage provides a reliable postharvest preservation system for kiwifruit. These technologies, combined with optimized harvest timing, biological controls, non-destructive monitoring, and pre-harvest hormone regulation, maintain physicochemical, nutritional, and sensory quality, extend shelf life, and reduce postharvest loss, supporting efficient supply chains and sustainable industrial development.

### 5.6 Ecological agricultural regulation measures

Ecological agricultural regulation plays a key role in sustainable kiwifruit cultivation by improving soil health, resource utilization, and environmental adaptability. Optimizing soil microbial communities is a core measure, as soil microorganisms promote nutrient cycling, organic matter decomposition, and the suppression of soil-borne pathogens, supporting root growth and stress resistance of kiwifruit. Long-term overuse of chemical fertilizers can lead to accumulation of potentially toxic elements (PTEs, e.g., Cd, Hg, Cu, Zn) in orchard soil, disturbing microbial balance and threatening fruit production safety (Chen et al., 2023b). Therefore, reducing excessive chemical inputs and applying organic amendments effectively improve soil microbial diversity and ecosystem stability. Maintaining understory biodiversity also helps preserve beneficial microbes and enhance soil fertility, improving crop adaptability under shifting climatic conditions (Ma et al., 2025b; Rajan et al., 2024).

In terms of soil microbial regulation and fertilization management, targeted measures have been proven effective in kiwifruit cultivation. For soil fungal community remediation, root rot in *Actinidia chinensis* is closely correlated with reduced fungal α-diversity (Chao1 index) and elevated relative abundance of pathogenic *Fusarium* and *Gibberella* (Chen et al., 2024a); accordingly, inoculation with *Trichoderma harzianum* can significantly enrich beneficial rhizosphere microbes and restore rhizosphere microecological homeostasis, alleviating root rot driven by fungal community dysbiosis (Shen et al., 2026). Beneficial microbial inoculants relieve plant oxidative stress mainly via triggering induced systemic resistance (ISR) and niche competition against phytopathogens beyond simply boosting soil nutrient availability. The compound bacterial inoculant consisting of nitrogen-fixing *Bacillus amyloliquefaciens* XD-N-3, phosphate-solubilizing *B. pumilus* XD-P-1 and potassium- solubilizing *B. circulans* XD-K-2 remarkably raises soil available N, P and K as well as *A. chinensis* biomass; partial substitution of chemical fertilizer at 50% rate with this inoculant optimizes soil fertility and mitigates fruit oxidative injury (Shen et al., 2016). In addition, nine consecutive years of organic fertilization (10–40 Mg/ha/year) increases kiwifruit yield by enriching beneficial genera such as Pseudomonas and Burkholderia while suppressing pathogenic Fusarium populations (Liu et al., 2020), further highlighting the advantages of organic substitution for conventional synthetic fertilizers.

Intercropping is another important ecological practice for kiwifruit orchards. It improves light, nutrient, and water use efficiency, increases soil microbial diversity, and reduces reliance on synthetic fertilizers and pesticides. Intercropping systems enhance biodiversity, promote natural pest control and pollination, and buffer microclimate extremes, thereby improving kiwifruit yield and quality while alleviating environmental pressure (Chen et al., 2023b; Ma et al., 2025b). In addition, intercropping supports the recycling of agricultural by-products, reduces waste, and aligns with circular economy principles to lower the environmental footprint of production (Chamorro et al., 2022). Specific intercropping practices for kiwifruit include mixed planting with *Schisandra chinensis*: studies have shown that mixed planting of *A. arguta* with *Schisandra chinensis* can increase proline and phenolics content in *A. arguta*, with the optimal ratio being 2:1 (*A. arguta:S. chinensis*) to minimize the inhibitory effect of S. chinensis root exudates on *A. arguta* root growth (Venediktova et al., 2022); however, a 50:50 mixture may reduce chlorophyll a content by 6.7–18.7% and alter phosphate and nitrogen metabolism in kiwifruit (Venediktova et al., 2024), indicating that rational intercropping ratio is crucial for ecological regulation.

Synthetic application of optimized fertilization, rhizosphere microbial remediation, rational intercropping and understory biodiversity conservation constructs eco-resilient kiwifruit orchard cultivation systems. All phenotypic and physiological differences of kiwifruit under distinct ecological cultivation modes are systematically quantified in **Table 2** based on six independent field trials. One representative study verified that mismatched intercropping regimes disrupt leaf pigment synthesis and nutrient homeostasis, highlighting the necessity of matched intercropping proportions for stable ecological orchard management (Venediktova et al., 2024).

**Table 2:** Effects of different ecological regulation measures on soil physicochemical properties, microbial communities, and kiwifruit productivity.

Management practice	Treatment (ratio / code)	Soil physicochemical properties	Soil microbial response	Plant growth / Fruit quality	Reference
Conventional monoculture	Mineral NPK control (CF) (287.5–115–115 kg ha^-^¹ yr^-^¹, 9 yr)	Baseline OM/N/P/K; Risk of heavy metal accumulation​ under long-term mineral-only input	Dominance of specific taxa; higher pathogen risk if OM not replenished	Standard yield baseline; higher chemical dependency	Liu et al., 2020
Monoculture (pathogenic state)	Root rot infection (*Natural field condition*)	Baseline OM/N/P/K; soil environment conducive to Fusariumproliferation	↓ Fungal diversity (Chao1 ↓**) Phylum: ↓ Basidiomycota, unclassified fungi; ↑ Ascomycota Genus: ↑ Gibberella, Nectria, Fusarium; ↓ beneficial taxa	↓ Fruit quality​ (impaired nutrient uptake) ↓ Yield​ (marketable loss)	Chen et al., 2024a
Microbial inoculation (*Pot trial, seedlings*)	Bacillus + Rhizobium (T3) (*Specific strain ratio*)	↑ OM (+30.3%); ↑ OC (+30.4%) ↑ TN (+6.8%); ↑ TK (+13.9%) pH slight ↓	↑ Ascomycota; enriched saprotrophs (*Plectosphaerella, Chaetomium*) ↓ *Mortierella*; fungal network shift	↑ Seedling biomass & SPAD​ (proxy for vigor) *No fruit harvest recorded*	Shen et al., 2026
Microbial inoculation (*Pot trial, seedlings*)	N_2_-fixing + P- + K-solubilizing (CI) (*1/2 CF + Full CI*)	↑ Available N/P/K; total N/P/K unchanged vs CF; improved soil available nutrient pool	↑ Abundance of functional PGPR (*B. amyloliquefaciens, B. pumilus, B. circulans*)	↑ Total biomass & N/P/K accumulation ↓ Leaf MDA (membrane stability) ↓ PPO (oxidative stress)	Shen et al., 2016
Intercropping	A. arguta× S. chinensis (2:1)	↑ Soil OM; ↑ Available N/P/K; Stable pH	↑ Beneficial microbes; ↓ Pathogenic Fusarium	↑ Proline; ↑ Total phenolics; Normal Chl a	Venediktova et al., 2022
Intercropping	A. arguta× S. chinensis (1:1)	Altered nutrient flux; potential OM depletion	Imbalanced microbiome; potential pathogen increase	↓ Chl a (6.7–18.7%); Altered N/P metabolism	Venediktova et al., 2024

Proline, proline; Total phenolics, total phenolic content; Chl a, chlorophyll a; Normal, no statistically significant difference compared to the control.

Definitions: CF (Chemical Fertilizer control): Conventional sole mineral NPK application without organic input (Liu et al., 2020).

Pot vs field: Two PGPR inoculation trials (Shen et al., 2016, 2026) are seedling pot experiments with no mature fruit harvest; all other treatments are field-orchard trials with yield/fruit data.

Symbol rules:​↑ = significant increase vs CF control; ↓ = significant decrease vs CF control; Normal = no statistical difference vs control; **P<0.01 significant level.

These integrated agronomic practices effectively improve crop stress resistance, ensure fruit safety and lower agrochemical pollution risks to support sustainable kiwifruit industry development. Nevertheless, existing ecological cultivation research still has obvious shortcomings: most intercropping trials are limited to single companion species under local climate conditions without cross-regional long-term monitoring data; the allelopathic molecular mechanisms between intercropped plants remain poorly resolved, and quantitative models for optimizing intercropping ratios according to soil fertility gradients are scarce. Future research should carry out multi-location long-term field trials, clarify allelopathy regulatory pathways, and build standardized regional ecological management protocols for kiwifruit orchards.

## 6 Genomics, molecular biology and gene family analysis

**Figure 6:**
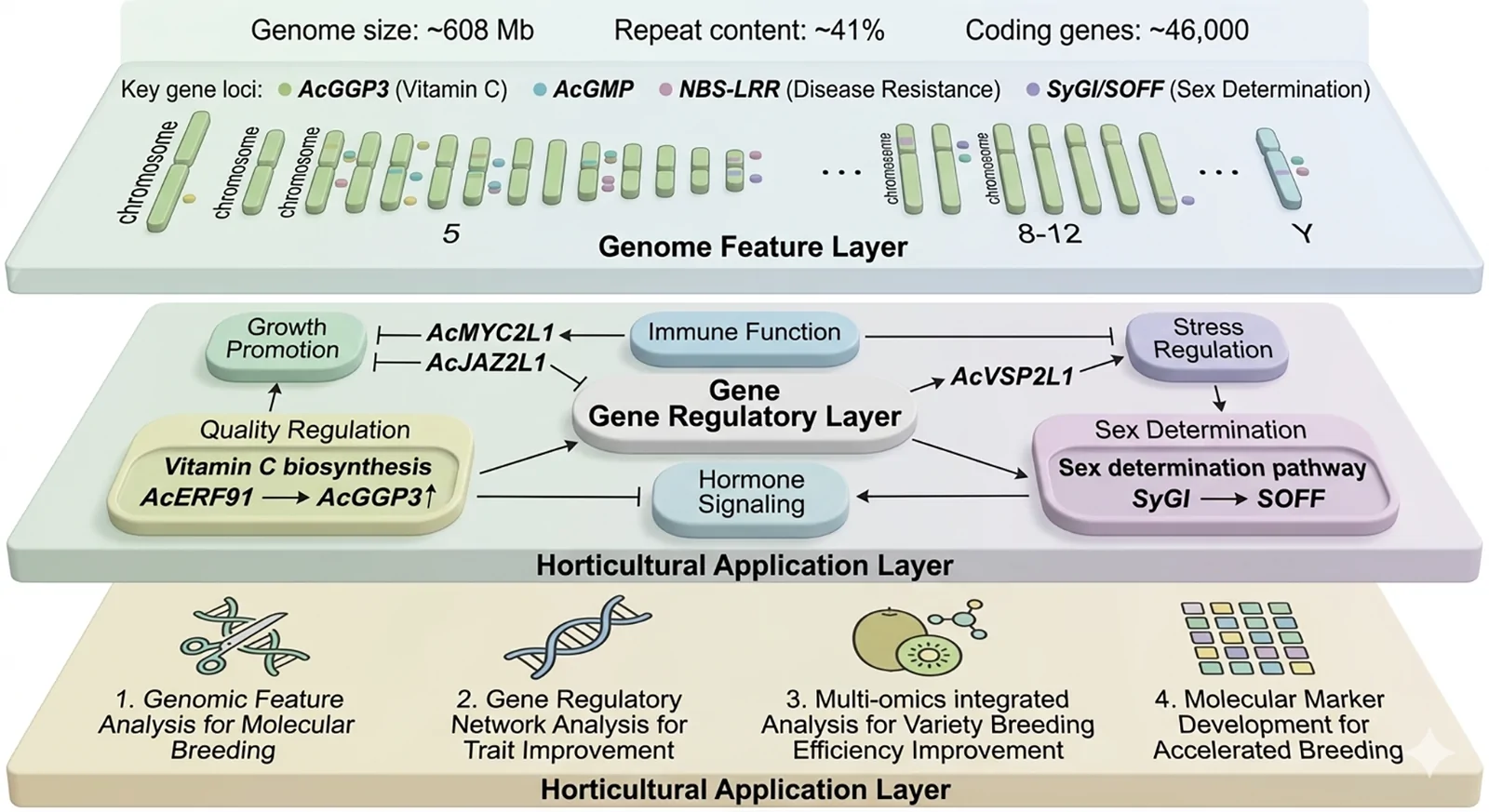
Schematic overview of the genomic features, key gene regulatory networks, and translational applications in *Actinidia chinensis*. This schematic stratifies kiwifruit genomic research into three hierarchical modules: assembled genome landscape with vital functional gene loci, multi-gene regulatory cascades governing nutritional quality, stress resistance and sexual differentiation, and diverse genomic analytical tools supporting targeted cultivar genetic improvement.

Telomere-to-Telomere (T2T) genome assemblies have deepened our understanding of genetic mechanisms governing major agronomic traits in *A. chinensis*. **Figure 6** presents a three-level research system integrating genome characterization, gene regulatory network analysis and breeding translation. This chapter summarizes advances in T2T genome assembly, stress-resistance functional genes and metabolic regulatory cascades, establishing a theoretical basis for precision molecular improvement of kiwifruit.

### 6.1 Kiwifruit genome construction and analysis

The completion of gap-free, haplotype-resolved genomes represents a milestone in kiwifruit genomic research. A high-quality chromosome-level assembly of *Actinidia chinensis* has been constructed, enabling investigations of whole-genome duplication and genome evolution (Wu et al., 2019). High-quality genomes of representative cultivars, including ‘Hongyang’ and ‘Guimi No. 2’, have greatly facilitated the dissection of key traits such as disease resistance and fruit quality. The ‘Guimi No. 2’ genome, assembled by PacBio HiFi and Hi-C technologies, has high completeness and enables gene annotation and comparative genomic analysis of *Pseudomonas syringae* pv. *actinidiae* (Psa) resistance across the *Actinidia genus* (Zhou et al., 2025).

The complete chloroplast genome of ‘Hongyang’ displays a typical quadripartite structure and supports phylogenetic and evolutionary studies (Liu et al., 2022b). Furthermore, a telomere-to-telomere gap-free assembly of *Actinidia eriantha* has been constructed with two resolved haplotypes and well-characterized centromeric regions, providing a high-quality foundation for genome mapping and functional genomics in kiwifruit (Yue et al., 2023). Meanwhile, the updated gap-free *A. chinensis* ‘Hongyang’ v4.0 assembly has further improved genome integrity and resolved complex genomic regions, supporting more accurate gene mining and evolutionary analysis (Wang et al., 2023c).

In polyploid kiwifruit, high-density genetic maps have been constructed using genotyping-by-sequencing (*GBS*), allowing QTL mapping for fruit weight, dry matter, and storage firmness in hexaploid cultivars (Popowski et al., 2021). A chromosome-scale assembly of diploid *A. chinensis* var. deliciosa has also provided insights into the evolutionary origin of allohexaploid varieties (Xia et al., 2023).

Gene family analyses have identified numerous transcription factor and defense-related families, including TIFY and *NBS-LRR*, which participate in stress responses and Psa resistance (Tao et al., 2022; Wang et al., 2020c). Plastid transformation systems have been established in *A. chinensis*, supporting stable transgene expression and molecular farming (Chen et al., 2025).

Pan-genomic analyses have revealed extensive structural variations and resistance gene analog (*RGA*) diversity, which underlie phenotypic divergence and adaptation (Yu et al., 2025). Haplotype-resolved assemblies have further uncovered regulatory mechanisms of vitamin C and sucrose metabolism (Wu et al., 2019). Multiple key agronomic genes have been characterized, such as sucrose accumulation-related *AcSWEET9b*, providing important targets for molecular breeding (Han et al., 2023).

Functional validation has linked gene function to fruit quality. The *β-carotene hydroxylase* (*AcBCH*) gene family regulates xanthophyll synthesis and flesh color, and has been verified via transgenic and *VIGS* experiments, offering valuable targets for quality improvement (Xia et al., 2022).

Genome data from T2T assemblies of ‘Donghong’ and ‘Kuoye’ facilitates the exploration of genetic mechanisms governing fruit nutritional quality. Expansion of the ERF098 gene family is closely associated with high ascorbic acid accumulation, while *AcSWEET9b* functions as a critical regulator of sucrose deposition in fruit. Further molecular characterization has confirmed that *AcUFGT6b* encodes a key xylosyltransferase responsible for anthocyanin modification, and two homologous *AcBCH* paralogs (*AcBCH1/2*) dominate β-carotene biosynthesis to determine yellow flesh formation. In ‘Hort16A’, coordinated regulation of chlorophyll degradation genes (*PAO2, SGR1*) and carotenoid metabolic genes (*LCYB2, CYP1, NCED1, CCD2*) jointly shapes the yellow-fleshed phenotype (Han et al., 2023; Liu et al., 2019; Xia et al., 2022; Zhang et al., 2018a).

Genomic resources also accelerate the excavation of functional genes underlying abiotic stress tolerance. Gamma-ray mutagenesis (^60^Co-γ) successfully produces heat-resistant ‘Hongyang’ mutants with lower reactive oxygen species accumulation. The cloned *AdCCS1* modulates Cu/Zn-SOD activity to strengthen antioxidant capacity, and systematic classification of 42 kiwifruit *AcDOF* members into five subfamilies clarifies that *AcDOF22* improves drought tolerance by transcriptionally activating *AcDREB2A* (Yuan et al., 2024; Chen et al., 2018; Zhao et al., 2024a).

Building on these genomic resources, genome-wide association studies (GWAS) have further enabled the dissection of complex agronomic and nutritional traits in kiwifruit. Using 946,337 high-quality SNP markers generated by simplified genome sequencing, a GWAS in 143 male *Actinidia eriantha* germplasms identified significant loci associated with floral traits (e.g., corolla diameter, pollen viability) and leaf quality traits (including ascorbic acid, chlorophyll, and flavonoid contents). Using the optimal MLM (QK) model, numerous candidate genes were anchored near significant SNPs, and trait-associated SNPs were further verified. This work provides valuable genetic resources and molecular markers for genomics-assisted breeding of kiwifruit (Liao et al., 2021).

High-density SNP genotyping chips and QTL mapping technology derived from reference genomes act as core bridges connecting genomic data and nutritional quality breeding. Target QTL intervals controlling vitamin C, flavonoid and soluble sugar accumulation have been precisely localized on chromosomes of major cultivated species, and tightly linked SNP markers within these regions can be developed as reliable molecular indicators (Wang et al., 2023b). Relying on these high-throughput genotyping platforms, breeders can carry out large-scale early population screening for nutritional traits at the seedling stage without waiting for fruit ripening, which greatly shortens the breeding cycle for high-nutrition kiwifruit varieties. Combined with the phytochemical trait data summarized in Chapter 3, this genome-to-breeding system realizes targeted genetic improvement oriented to bioactive compound enrichment, and effectively strengthens the practical horticultural value of genomic research as requested in this review.

In summary, high-quality genome assemblies, gene family characterization, and pan-genomic resources have established a solid foundation for understanding kiwifruit biology. These multi-level genomic tools support the excavation of stress-resistance and fruit quality genes, facilitate GWAS analysis and high-density marker development, and greatly accelerate marker-assisted breeding and biotechnological improvement for high-nutrition, stress-tolerant kiwifruit germplasm.

### 6.2 Gene expression regulatory networks

Gene expression during kiwifruit (*Actinidia chinensis*) ripening and secondary metabolism is regulated by transcriptional, post-transcriptional, and epigenetic mechanisms. Recent studies highlight critical roles of long non-coding RNAs (*lncRNAs*) and alternative splicing in fruit ripening. Differentially expressed lncRNAs during ABA-induced softening are associated with starch and sucrose metabolism, plant hormone signaling, and flavonoid biosynthesis. These lncRNAs regulate the expression of genes encoding cell wall-modifying enzymes and NAC transcription factors, thereby mediating fruit softening (Chen et al., 2021c). Alternative splicing of regulatory genes such as *NAP1* also contributes to phenotypic variation by affecting transcript structure and function (Miao et al., 2023). Together, lncRNAs and alternative splicing fine-tune metabolic pathways that determine fruit quality, including flavonoid and anthocyanin accumulation (Mao et al., 2024).

Phytohormone crosstalk between ethylene and auxin is central to kiwifruit ripening and stress responses. Ethylene signaling governs ripening processes including texture, color, and aroma formation. Ethylene response factors (*ERFs*) such as *AcERF91* regulate ascorbate metabolism by activating *AcGGP3*, thus affecting vitamin C biosynthesis (Chen et al., 2021d). Ethylene also induces terpene synthase genes that contribute to volatile aroma formation via ERF-mediated regulatory networks (Wang et al., 2021c).

Auxin (IAA) signaling is equally critical in fruit development and defense. Silencing of the auxin response factor gene *AcARF18* enhances resistance to *Botrytis cinerea*, accompanied by changes in IAA and salicylic acid (SA) levels, indicating crosstalk between auxin and stress responses (Li et al., 2025c). The co-repressor gene *AcTPR2* modulates defense by suppressing IAA signaling, highlighting tight integration of auxin regulation during pathogen stress (Li et al., 2020).

Furthermore, ethylene-activated NAC transcription factors induce cell wall−modifying *XTH* genes, linking hormonal signaling to fruit softening (Fu et al., 2024). The synergistic and antagonistic interactions between ethylene and auxin pathways coordinately control cell wall degradation, secondary metabolism, and stress tolerance, shaping the ripening process and adaptive responses. Uncovering these regulatory networks supports genetic improvement of fruit quality and stress resistance in kiwifruit.

The transcriptional modules regulating vitamin C, flavonoids and ursane-type triterpenoids link phytochemical traits to breeding objectives. Core biosynthetic genes and transcription factors for these metabolites have been anchored on kiwifruit chromosomes (Duan et al., 2026). Varietal differences in metabolite abundance act as visible screening markers for high-nutrient germplasm. Using these gene-metabolite associations, marker-assisted selection (MAS) and genomic selection (GS) can be implemented: trait-specific SNP/QTL markers derived from key loci allow early seedling screening before fruiting. Such an integrated strategy speeds up the targeted breeding of kiwifruit with superior antioxidant contents and stress tolerance, improving the industrial applicability of multi-omics research.

### 6.3 Antistress genes and their regulatory mechanisms

Beyond the hormone-dependent regulatory networks controlling fruit ripening described above, ethylene–auxin signal cascades also serve as core signaling hubs for kiwifruit coping with diverse biotic and abiotic adversities (Li et al., 2025c; Fu et al., 2024). Accordingly, multi-omics approaches have excavated abundant stress-responsive genes functioning at distinct regulatory layers to build systematic stress-resistant networks in *Actinidia chinensis*.

At the cellular ion homeostasis level, the Shaker-type K^+^ channel family contains 28 members in ‘Hongyang’. Among these, *AcShaker15*, *AcShaker17*, and *AcShaker22* are upregulated under low-K^+^ (drought-mimicking) stress, whereas *AcShaker11* and *AcShaker18* are suppressed under salt stress, revealing their essential roles in maintaining ionic balance against osmotic pressure (Zi et al., 2024). Collectively, these plasma membrane-located ion channels constitute the first defensive barrier to stabilize intracellular environment upon environmental fluctuation.

Upstream of ion transport, multiple transcription factor families orchestrate downstream stress-related transcription. The 42-member DOF transcription factor family participates broadly in abiotic stress adaptation. *AcDOF22*, predominantly expressed in drought-tolerant accessions, confers drought resistance via transcriptional activation of *AcDREB2A*, which alleviates water loss and chlorophyll breakdown; abundant ABA-, MeJA- and stress-related cis-elements in its promoter imply multifaceted hormonal modulation, and several DOF paralogs are also inducible under cold stress (Zhao et al., 2024a). Under waterlogging stress, the transcription factor *AcMYB68* positively controls pyruvate decarboxylase (*PDC*, first defined here) genes for anaerobic metabolism; its overexpression accelerates anaerobic metabolism but restrains root elongation, and restricted expression of *AcMYB68* in tolerant genotypes improves waterlogging survival (Chen et al., 2024b).

Post-transcriptional regulation mediated by microRNAs further enriches the stress regulatory cascade. *Ac-miR160d* elevates gray mold resistance by rearranging endogenous IAA and SA content, boosting antioxidant enzyme activity and promoting defensive secondary metabolite synthesis via targeted inhibition of auxin signaling (Li et al., 2023a). At the cell-surface signal perception layer, the leucine-rich repeat receptor-like kinase (LRR-RLK, first defined here) superfamily consists of 394 members, with 48 core genes (especially subgroup XII) specifically triggered by *Pseudomonas syringae* pv. *actinidiae* (Psa) invasion (Li et al., 2025b).

Organelle RNA editing is another indispensable regulatory mode for pathogen tolerance. Pentatricopeptide repeat (*PPR*) proteins govern organellar RNA editing, with divergent editing efficiency and transcript abundance between resistant and susceptible cultivars under pathogen attack, whose transcription is further modulated by upstream transcription factors (Zhang et al., 2023). Meanwhile, multiple organelle-targeted MORF family proteins (first defined here) modulate chloroplast RNA editing and disease resistance, showing cultivar-specific expression divergence upon pathogen infection (Xiong et al., 2022).

Beyond biotic and abiotic stress responses, recent research has uncovered a core thermotolerance mechanism in kiwifruit (Zhang et al., 2026). Mild-to-extreme heat pretreatment significantly enhances root vitality and photosynthetic recovery. Mechanistically, heat acclimation induces the transcription factor AcLBD1, which activates a mutual positive feedback loop between *AcHSFA2–1* and *AcHSFA2-2*. This cascade amplifies the expression of heat-protective *AcHSP20* genes; notably, overexpression of *AcHSFA2–2* dramatically increases root vitality by up to 4.13-fold, establishing the LBD–HSFA2–HSP20 signaling axis as a central determinant of heat resilience and a promising target for molecular breeding.

Overall, the sophisticated regulatory network consisting of transcription factors, miRNAs and functional genes precisely coordinates environmental signals with hormonal and metabolic pathways, providing abundant stress-tolerant genetic resources for marker-assisted selection and genomic selection. Stress-related SNPs and QTLs support early seedling screening and directional genetic improvement of kiwifruit resistance to bacterial canker, drought, salinity and heat, thereby shortening breeding cycles and facilitating sustainable orchard production.

Nevertheless, three prominent technical bottlenecks restrict the translational application of current genomic research outcomes, which correspond to the core limitations pointed out in recent systematic omics studies. First, functional verification of candidate resistance genes remains highly constrained by immature transformation systems in Actinidia. Stable genetic transformation efficiency varies drastically across species including A. valvata and A. chinensis, and imperfect CRISPR/Cas genome editing platforms largely hinder *in vivo* gene function characterization (Xia et al., 2026). Second, existing multi-omics datasets are mostly analyzed in isolation, lacking standardized integration pipelines; most current omics outputs only identify correlative molecular markers instead of clarifying causal adaptive regulatory pathways (Zhang et al., 2023). Third, massive genomic variant loci have been mined, yet their practical utilization in field-scale molecular breeding is severely limited by complex polyploid genetic backgrounds, which reduce the prediction accuracy of genomic selection models in hybrid populations (Mertten et al., 2025). Apart from the above three genome-oriented drawbacks, current studies still have evident limitations. Most stress regulatory pathways are characterized under single-stress laboratory conditions, while the molecular crosstalk underlying combined biotic and abiotic stress tolerance remains poorly understood. In addition, most identified stress-resistance loci lack systematic functional verification and multi-site field evaluation, restricting their practical application in molecular breeding.

## 7 Clinical and health applications of kiwifruit

**Figure 7:**
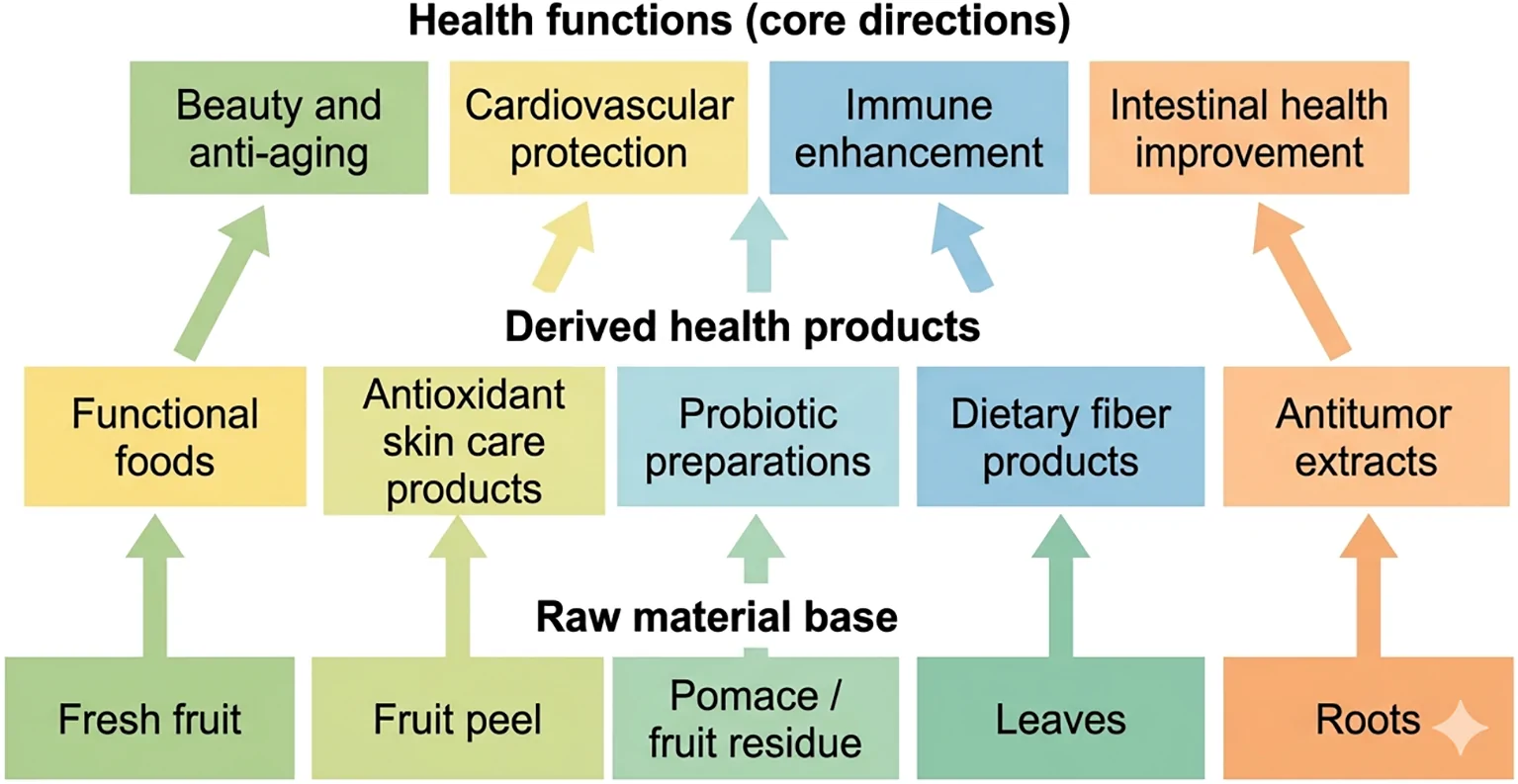
Industrial development framework of functional foods and health products from *Actinidia chinensis*. This diagram illustrates a three-tier value chain for comprehensive utilization of kiwifruit bioresources, covering various tissue raw materials, diversified deep-processed products and their distinct health-promoting functions, which collectively demonstrate the industrial exploitation potential of kiwifruit functional metabolites.

Genotype, cultivation environment and tissue-specific metabolism shape the unique bioactive profiles of kiwifruit, laying a material foundation for health-oriented product development and resource recycling. Current research reveals multi-functional health benefits of kiwifruit extracts, yet reliable clinical evidence supporting therapeutic usage remains limited. **Figure 7** summarizes diverse raw material resources derived from different kiwifruit tissues for downstream deep processing. This chapter integrates research on health functions, clinical research progress and industrial transformation routes, to offer theoretical guidance for germplasm innovation, full-resource utilization and industrial upgrading of kiwifruit.

### 7.1 Antitumor and anti-aging product development

The development of kiwifruit (*Actinidia chinensis*)-derived functional foods and natural skincare products has attracted increasing attention, owing to its rich bioactive compounds (polysaccharides, flavonoids, phenolic acids) with antioxidant, anti-inflammatory, and anti-aging properties. These components neutralize free radicals, reduce oxidative stress (a key factor in skin aging and cellular senescence), and hold potential for nutraceutical and cosmeceutical applications. Phenolic-rich extracts from kiwifruit roots and peels, obtained via optimized methods like infrared irradiation, exhibit enhanced antioxidant and antibacterial activities, benefiting skin health (Shkeir et al., 2024). Additionally, these bioactive compounds promote collagen synthesis and protect against UV-induced damage, making them promising for natural anti-aging formulations.

*Actinidia chinensis* polysaccharides, mainly extracted from roots, suppress tumor growth by regulating immune responses and inhibiting angiogenesis (critical for tumor progression). For example, Radix *Actinidiae chinensis* dose-dependently inhibited neovascularization in colorectal cancer models, reducing tumor mass and vascular marker CD31 (Meng et al., 2026). Flavonoids and phenolics exert cytotoxic effects by inducing apoptosis, arresting cell cycles, and suppressing oncogenic signaling. Ethanol extracts of kiwifruit roots inhibited colon cancer cell proliferation, migration, and invasion by downregulating Notch signaling (Notch1, Jagged1, c-Myc) and promoting apoptosis (Hu et al., 2021). In breast cancer cell lines, root extracts reduced cyclin D1 and Bcl-2 (proliferation/survival proteins) and enhanced pro-apoptotic Bax via inhibiting the AKT/GSK-3β pathway (Gan et al., 2021). Moreover, HSPA6 regulates lung cancer cell sensitivity to kiwifruit root extract, representing a potential target to improve therapeutic efficacy (Wang et al., 2020b). Collectively, these findings support *A. chinensis*-derived bioactive compounds as natural antitumor agents, laying a scientific foundation for their development in cancer chemoprevention and adjunct therapy.

### 7.2 Cardiovascular protection and immune regulation applications

Kiwifruit (*Actinidia chinensis*) shows potential in cardiovascular disease (CVD) prevention, thanks to bioactive compounds likevitamin C, polyphenols, dietary fiber, and antioxidants. These components protect cardiovascular health via multiple pathways: polyphenols and vitamin C neutralize reactive oxygen species (ROS), reducing oxidative damage to vascular endothelium; dietary fiber lowers low-density lipoprotein cholesterol (LDL-C) by binding bile acids; and they reduce pro-inflammatory cytokines such as TNF-α, stabilizing atherosclerotic plaques. A systematic review and meta-analysis of randomized controlled trials further confirmed that kiwifruit intake improves metabolic indicators (including blood lipids and glucose) in populations with CVD risk factors, reinforcing its role in cardiovascular health (Shkeir et al., 2024). Though clinical trials targeting cardiovascular outcomes are limited, cumulative nutritional and clinical evidence supports kiwifruit as a functional food for CVD prevention (Eady et al., 2020).

Kiwifruit also has notable immunomodulatory properties. Clinical studies show that consuming whole Zespri^®^ SunGold kiwifruit (including skin) reduces TNF-α in healthy individuals and irritable bowel syndrome with constipation (IBS-C) patients, maintaining immune homeostasis. These effects stem from vitamin C, polyphenols, and dietary fiber: vitamin C enhances phagocytosis and lymphocyte proliferation, while fiber improves gut health and microbiota composition to regulate systemic immunity (Suksomboon et al., 2019). Additionally, research on golden kiwifruit (*Actinidia chinensis* ‘Hort16A’) supports its immune-supporting effects: it up-regulates immune-related and DNA repair-related gene sets, down-regulates gene sets associated with immunoglobulin secretion, and enhances innate and adaptive immune function in preclinical studies (Skinner et al., 2011). For elderly populations with declining immunity, daily consumption of four ‘Hort16A’ kiwifruits significantly increases plasma vitamin C and antioxidant levels, shortens the duration of upper respiratory tract infection symptoms, and reduces symptom severity, highlighting its targeted immune-protective role in vulnerable groups (Hunter et al., 2012). It also alleviates IBS-C gastrointestinal symptoms, showing dual gut-immune benefits. Further research is needed to clarify detailed molecular mechanisms and expand clinical applications (Eady et al., 2020).

### 7.3 Functional health products and food additives

The development of functional health products and food additives from *Actinidia chinensis* (kiwifruit) has attracted growing interest due to its abundant bioactive compounds and health-promoting properties. Research has focused on optimizing fermentation and extraction processes to improve the yield and bioactivity of polysaccharides, flavonoids, and phenolics. Ultrasound-assisted enzymatic extraction has been refined to enhance the recovery of kiwifruit starch and polyphenols with higher yield, purity, and antioxidant activity, supporting its application as a functional food ingredient (Wang et al., 2022a). Microwave drying also outperforms conventional oven drying, increasing total phenolic content and antioxidant activity while preserving key bioactive compounds such as catechins in dried kiwifruit products (Özcan et al., 2020).

Valorization of kiwifruit by-products, including peels, seeds, and pomace, supports sustainable resource utilization. These by-products are rich in phenolics, flavonoids, carotenoids, and polar lipids with strong antioxidant, anti-inflammatory, and antithrombotic activities. Peel extracts effectively inhibit platelet aggregation and show favorable fatty acid profiles, making them promising nutraceutical components (Moysidou et al., 2025). Fermentation of kiwifruit by-products with *Lacticaseibacillus casei* 431^®^ and prebiotic flours produces stable probiotic ingredients with controlled polyphenol release, suitable for functional foods such as protein bars (Ilie et al., 2022). Probiotic-incorporated konjac glucomannan edible films also improve the quality and shelf life of fresh-cut kiwifruit by enhancing antioxidant activity and reducing decay (Hashemi and Jafarpour, 2021).

Kiwifruit-derived polysaccharides and phenolics are promising functional additives with well-characterized antioxidant and anti-inflammatory effects. Agricultural and industrial by-products rich in vitamin C and phenolics offer prebiotic and digestive health benefits, supporting circular economy and sustainable production (Liu et al., 2020). Bioactives from kiwifruit have also been incorporated into bioplastics and food packaging materials such as *polybutylene succinate* films, effectively reducing browning and microbial spoilage in fresh-cut kiwifruit (Barrino et al., 2023).

In summary, advanced extraction, fermentation and biomaterial processing strategies enable the high-value reuse of kiwifruit peels, seeds and pomace, facilitating the development of functional food additives, probiotic products and sustainable food packaging and greatly promoting the circular utilization of kiwifruit industrial resources. Nevertheless, current by-product utilization research still has evident limitations: most studies focus on laboratory-scale material preparation and *in vitro* functional evaluation, while standardized industrial processing systems and parameter optimization for large-scale production are lacking. In addition, the bioavailability, metabolic transformation and long-term edible safety of kiwifruit by-product bioactives remain insufficiently demonstrated, and consumer acceptance and market application potential are rarely quantitatively assessed. Future research should focus on industrial-scale process optimization, systematic safety and bioavailability verification, and market-oriented applicability evaluation, to accelerate the commercial transformation and large-scale industrial application of kiwifruit by-product-derived functional ingredients.

## 8 Discussion and prospects

**Figure 8:**
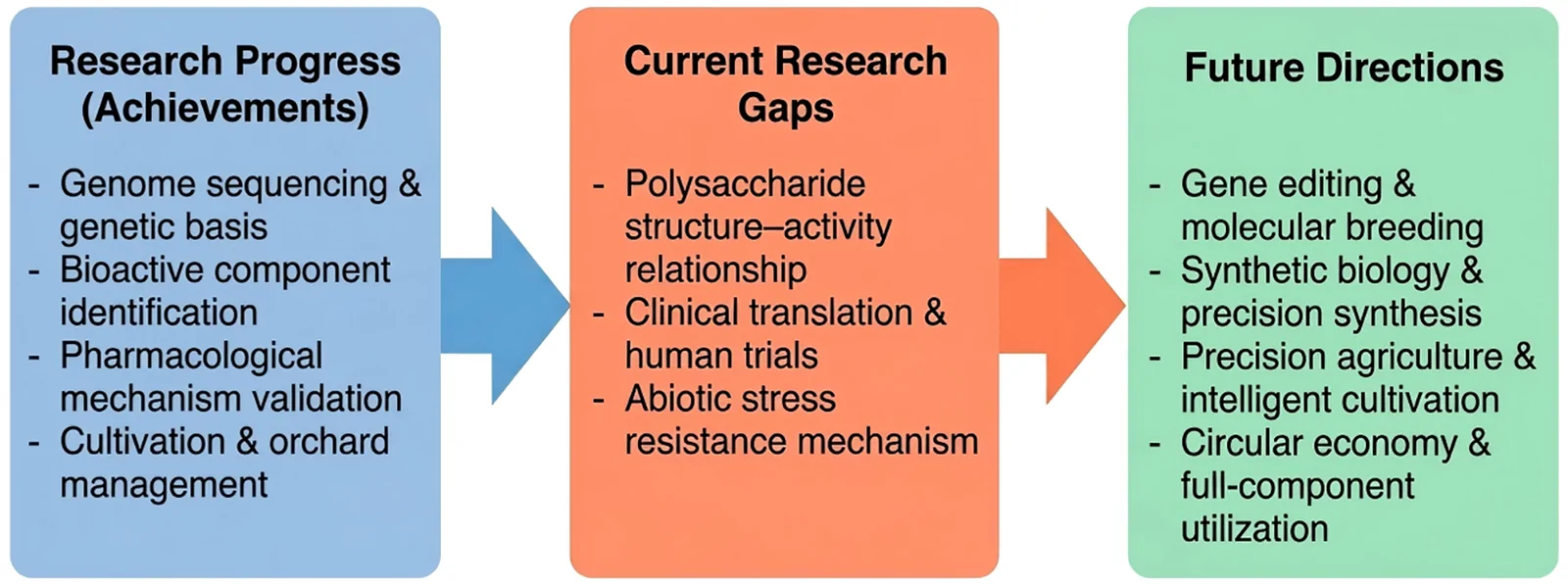
Overview of research progress, unresolved knowledge gaps and prospective research directions for Actinidia chinensis. This three-module roadmap systematically summarizes core advances in kiwifruit genomics, phytochemistry, pharmacology and cultivation management. It outlines prominent research bottlenecks including ambiguous polysaccharide structure–activity correlations, insufficient clinical translation data and unclear abiotic stress regulatory pathways, and highlights emerging research avenues such as gene editing-assisted breeding, synthetic biology, precision horticulture and circular full-resource exploitation.

Decades of research have expanded our understanding of A. chinensis from basic genomics to practical industrial cultivation. **Figure 8** integrates the full research framework spanning genome analysis, phytochemical identification, pharmacological exploration and horticultural management. Many unresolved challenges still hinder deep exploitation of kiwifruit resources. This chapter summarizes prevailing research limitations and proposes targeted future research directions to support sustainable, high-quality industrial development.

### 8.1 Key findings and research gaps

Existing research on *Actinidia* spp. has established a solid framework covering botanical characteristics, phytochemistry, pharmacology, agricultural practices, and clinical applications, but notable gaps remain. In phytochemistry, triterpenoids, flavonoids, and polysaccharides have been identified as core bioactive components (Chen et al., 2021b; Wang et al., 2021b), yet the structural diversity of polysaccharides and their structure-activity relationships are poorly characterized. Although ultrasound-assisted extraction has been optimized to obtain proanthocyanidin-rich fractions from kiwifruit leaves (Lv et al., 2021), the precise molecular weights and monosaccharide compositions—key factors affecting bioavailability and efficacy—need further elucidation.

In pharmacology, kiwifruit’s antitumor and anti-inflammatory effects have been validated *in vitro* and *in vivo*, but translation to clinical practice is limited by insufficient large-scale trials. For example, ethanol extracts from *A. chinensis* roots inhibit colorectal cancer via suppressing the *Notch* signaling pathway (Zhou et al., 2010), and astragalin from *A. chinensis* induces liver cancer cell apoptosis (Hong et al., 2021). However, the optimal human dosage, administration route, and potential side effects of these extracts remain unclear. Additionally, the synergistic effects between different bioactive components (e.g., vitamin C, phenylpropanoids, alkaloids) need systematic exploration to explain kiwifruit’s holistic pharmacological activities (Wang et al., 2021b).

In agricultural applications, Psa resistance breeding has advanced via QTL mapping and genome editing (Hu et al., 2021; Meng et al., 2026), but resistance durability in polyploid kiwifruit remains a challenge. The *Actinidia* super pan-genome has revealed extensive structural variations and *RGAs* (Yu et al., 2025), but functional validation of these RGAs under field conditions is lacking. Ecological cultivation measures (intercropping, soil microbial regulation) show sustainability potential (Chen et al., 2023b; Labra Cordero, 2022), but their adaptability to different climates and long-term impacts on fruit quality require further verification.

### 8.2 Future research directions

Multidisciplinary integration will be the core driver of future kiwifruit research and industrial progress. This section constructs a systematic, tiered research roadmap covering four indispensable dimensions: emerging innovative technologies, unresolved core scientific questions, interdisciplinary collaborative opportunities, and industrial commercialization bottlenecks, to clarify targeted research priorities for subsequent work.

#### 8.2.1 Emerging core innovative technologies

##### 8.2.1.1 Modern molecular breeding biotechnologies

Gap‑free telomere‑to‑telomere genomes, multi‑omics datasets, and the dissection of genetic‑epigenetic mechanisms underlying climate adaptation from wild germplasm have laid a solid foundation for trait improvement (Han et al., 2023; Yue et al., 2023; Zhang et al., 2023). Four state-of-the-art breeding tools deserve priority application:

1. CRISPR/Cas genome editing: Optimize polyploid-compatible transformation systems to edit key genes such as AcJAZ2L2, SOFF/SyGI and NBS-LRR for bacterial canker resistance, sex control and fruit nutritional quality; multi-gene pyramiding editing will create multi-stress resistant elite germplasm (Xia et al., 2026; Wang et al., 2025).
2. Genomic selection: High-density SNP chips and polyploid prediction models enable accurate evaluation of complex agronomic traits at seedling stage, drastically shortening breeding cycles (Mertten et al., 2025).
3. Speed breeding: Regulate light, temperature and photoperiod regimes to compress growth cycles, achieving multiple generations per year and accelerating purification of edited mutant and hybrid populations.
4. AI-assisted intelligent breeding: Machine learning combined with multi-omics and high-throughput phenotyping automatically screens superior alleles, predicts combining ability and designs hybrid combinations, forming full-process digital breeding workflows.

AcSWEET9b’s allele-specific sucrose regulatory mechanism exemplifies how these biotechnologies can be deployed to breed high-sugar, high-nutrient cultivars (Han et al., 2023).

##### 8.2.1.2 Advanced separation and agricultural technologies

Novel green extraction approaches including electromembrane and thin film microextraction can isolate high-purity phytochemicals for structural and functional verification (Asadi et al., 2021; Ahmadi et al., 2025). Meanwhile, precision agriculture tools (IoT monitoring, computer vision fruit detection, bio-inspired robots and ecological niche modeling) optimize orchard resource allocation and climate adaptability at low carbon cost (Rajan et al., 2024; Kondoyanni et al., 2022).

#### 8.2.2 Unresolved key scientific questions

Multiple fundamental gaps still restrict theoretical breakthroughs across research fields:

1. Phytochemistry & pharmacology: The synergistic effects and precise structure–activity relationships of mixed triterpenoids, flavonoids and polysaccharides remain unclear; most *in vitro*/vivo bioactivities rely on supraphysiological doses, with insufficient standardized human clinical trials for anti-tumor, lipid-lowering and immunomodulatory effects (only intestinal benefits have reliable clinical evidence).
2. Molecular stress biology: Most stress regulatory pathways are characterized under single laboratory stress; the crosstalk mechanisms of combined biotic and abiotic stresses are poorly elucidated, and most resistance loci lack multi-site field validation (Zhang et al., 2026).
3. Metabolic regulatory networks: The coordinated gene modules controlling fruit flavor, anthocyanin and vitamin C accumulation require deeper multi-omics integration; epigenetic regulation of quality traits under cultivation conditions remains understudied.
4. Postharvest biology: Uniform storage and ripening regulation standards for multi-cultivar kiwifruit are absent, and the molecular basis of chilling injury needs further clarification.

#### 8.2.3 Interdisciplinary collaborative opportunities

Cross-field integration can break single-discipline limitations and generate innovative research outputs:

1. Genomics × Food processing: Combine pan-genome data with green extraction technology to directional screen high polyphenol/polysaccharide germplasm, and develop targeted separation protocols for by-product active ingredients.
2. Plant physiology × Clinical nutrition: Link fruit nutrient accumulation rules with human intervention trials to develop special functional foods for the elderly, sub-health and metabolic populations; existing evidence shows golden kiwifruit relieves upper respiratory symptoms in seniors, which can be extended to immunocompromised groups (Hunter et al., 2012).
3. Horticulture × Microbial ecology: Integrate orchard soil microbiology and fruit fermentation research to develop organic fertilization, biocontrol and probiotic fermentation technologies (Chamorro et al., 2022; Ilie et al., 2022).
4. Digital agriculture × Synthetic biology: Deploy AI big data combined with synthetic biology to design engineered strains for efficient extraction of kiwifruit bioactives.

#### 8.2.4 Industrial commercial challenges and countermeasures

A series of practical bottlenecks hinder large-scale industrial transformation of laboratory research results:

1. Germplasm industrialization: Severe cultivar homogenization, lack of proprietary elite varieties, and immature polyploid breeding pipelines slow commercial promotion of new strains.
2. Deep-processing constraints: Most extraction experiments stay at lab scale; pilot and industrial standardized purification systems are missing, while peel/pomace by-products lack stable, high-value product lines (Zhou et al., 2024).
3. Clinical transformation barriers: High cost of human trials, insufficient long-term edible safety data of kiwifruit extracts limit the development of oral health products.
4. Market standardization gaps: Unified quality evaluation markers for raw materials and processed products are not established, and international brand competitiveness is weak for domestic kiwifruit products.

To address the above challenges, six long-term priority research directions are proposed:

1. Develop multi-trait resistant germplasm via CRISPR, genomic selection and AI-assisted speed breeding
2. Clarify core metabolic and stress-resistance gene networks through integrated multi-omics;
3. Establish standardized green industrial extraction systems for fruit and agricultural waste;
4. Conduct standardized population clinical trials to validate systemic health benefits of kiwifruit bioactives;
5. Build full-chain intelligent ecological orchard systems supported by digital agriculture;
6. Realize circular economy high-value utilization of kiwifruit peel, pomace and thinned unripe fruits.

### 8.3 Industrial and resource utilization prospects

The industrialization of kiwifruit by-product and processed products holds great promise, yet bottlenecks remain in standardized extraction techniques, bioactivity retention and mature product formulation. Microencapsulation technology effectively protects thermolabile vitamin C and phenolic compounds during processing and storage, retaining original biological activity of raw materials (Özcan et al., 2020). Based on abundant peel-derived bioactive substances, kiwifruit resources can be further developed into probiotic fortified functional food raw materials and natural plant-derived cosmetic additives (Shkeir et al., 2024).

Uniform quality evaluation criteria are indispensable for advancing kiwifruit resource industrialization. Specific chemical markers including corosolic acid and astragalin can be set as core quality indexes to formulate unified content specifications and rapid on-site detection protocols for raw fruit and processing by-products (Kou et al., 2021; Hong et al., 2021). Close cooperation between breeding research institutes and fruit-processing enterprises facilitates the transformation of superior germplasm achievements into commercial cultivars and high-value by-product products, boosting sustainable development of the whole kiwifruit industrial chain.

In conclusion, *Actinidia* represents a multi-purpose horticultural crop with prominent developmental potential across breeding, cultivation and deep-processing fields. Future research ought to fill current research gaps, promote lab-to-field translational research and realize full-component sustainable utilization of kiwifruit resources via interdisciplinary and advanced technical integration, contributing to eco-friendly and high-efficiency development of global kiwifruit industry.
