# Supplementary material for: From genomics to horticultural innovation: botanical traits, bioactive compounds, and sustainable utilization of kiwifruit (Actinidia spp.)
Source: Front Plant Sci. 2026 Aug 17;17:1903424. doi: 10.3389/fpls.2026.1903424 (PMC13528668; doi:10.3389/fpls.2026.1903424)
Supplement: Supplementary file 1 [file Table1.docx]

| S.Table S1. Phytochemical constituents from different parts of *Actinidia chinensis* and their biological activities. To sum up, after removing duplicates, a total of 173 unique compounds were isolated and identified from different tissues of *A. chinensis*. | | | | | | |
| --- | --- | --- | --- | --- | --- | --- |
| NO | Name | Part | Type of Products | New | Ref | Cancer Cells,*IC_50_* (*μ*M ) or Other Effects |
| 1 | (2α,3*β*)-2,3,23-trihydroxyurs-13(18)-en-28-oic acid (1) | roots | ursane-type triterpenoid | * | (Xu et al. 2016) | — |
| 2 | 2α,3*β*,19α,23-tetrahydroxyurs-12-en-28-oic acid 28-O-*β*-D-glucopyranoside (2) | roots | ursane-type triterpenoid | + | Ditto | — |
| 3 | 2α,3*β*,23-trihydroxyursa-12,20(30)-dien-28-oic acid (3); | roots | ursane-type triterpenoid | + | Ditto | — |
| 4 | 2α,3α,23,24-tetrahydroxyurs-12-en-28-oic acid (4); | roots | ursane-type triterpenoid | + | Ditto | — |
| 5 | sphingolipid (5)+; | roots | lipid | + | Ditto | — |
| 6 | 3*β*-hydroxystigmast-5-en-7-one (6)+; | roots | phytosterol | + | Ditto | — |
| 7 | dotriacontanic acid (7)+ | roots | fatty acid | + | Ditto | — |
| 8 | 4,4’-dihydroxyl-dihydrochalcone-2’-O-*β*-D-glucopyranoside (8) | roots | flavonoid | - | Ditto | 10ug/ml:CYP2C9,*67.9* |
| 9 | 12α-chloro-2α,3*β*,13*β*,23-tetrahydroxyolean-28-oic acid-13-lactone (9); | roots | oleanane-type triterpenoid | - | Ditto | 10ug/ml:CYP2C9 ,*67.1*; CYP3A4,*9.8*; |
| 10 | 2*α*,3*β*-dihydroxyurs-12-en-28-oic acid (10) | roots | ursane-type triterpenoid | - | Ditto | 5ug/ml:CYP3A4,*56.6*;  50ug/ml:CYP2C19 ,*69.3*;CYP3A4,*39.3*; |
| 11 | 2*α*,3*α*,24-trihydroxyurs-12-en-28-oic acid (11) | roots | ursane-type triterpenoid | - | Ditto | 5ug/ml:CYP3A4,*52.0*;  50ug/ml:CYP2C19 ,*75.0*;CYP3A4,*35.0*; |
| 12 | 2*α*,3*α*,24-trihydroxyolean-12-en-28-oic acid (12) | roots | oleanane-type triterpenoid | - | Ditto | 10ug/ml:CYP2C9,*28.3*;CYP2C19,*59.9*;CYP2D6,*31.8*; CYP3A4,*37.1*; |
| 13 | 2*α*,3*β*,23-trihydroxyurs-12-en-28-oic acid (13) | roots | ursane-type triterpenoid | - | Ditto | 10ug/ml:CYP2C9,*59.8*;CYP3A4,*55.2*; |
| 14 | 2*α*,3*α*,23,24-tetrahydroxyursa-12,20(30)-dien-28-oic acid (1)* | roots | ursane-type triterpenoid | * | (Wei et al. 2018) | HepG2,*19.62*;A-549,*18.86*;HeLa,*28.74* |
| 15 | 2*α*,3*α*,24-trihydroxyursa-12,20(30)-dien-28-oic acid (2) | roots | ursane-type triterpenoid | - | Ditto | A-549,*16.63*;SK-OV-3,*22.91*;HeLa,*15.27* |
| 16 | 2*α*,3*α*,24-trihydroxyurs-12-en-28-oic acid (3); **(11)** | roots | ursane-type triterpenoid | - | Ditto | HepG2*,12.22* |
| 17 | 2*α*,3*α*,19*α*,24-tetrahydroxyurs-12-en-28-oic acid (4) | roots | ursane-type triterpenoid | - | Ditto | A-549,*22.60*;HeLa,*29.35* |
| 18 | 2*α*,3*β*,23,24-tetrahydroxyurs-12-en-28-oic acid (5) | roots | ursane-type triterpenoid | - | Ditto | HepG2,*11.76*;MCF-7,*12.00*;SK-OV-3,*10.30* |
| 19 | 2*α*,3*β*,23-trihydroxyursa-12,20(30)-dien-28-oic acid (6); **(3)** | roots | ursane-type triterpenoid | - | Ditto | SK-OV-3,*16.33* |
| 20 | 2*α*,3*β*,19*α*,23-tetrahydroxyurs-12-en-28-oic acid (7) | roots | ursane-type triterpenoid | - | Ditto | HepG2,*19.08*;HeLa,*15.05* |
| 21 | 2*α*,3*β*-dyhydroxyolean-12-en-28-oic acid (8) | roots | oleanane-type triterpenoid | - | Ditto | — |
| 22 | 2*α*,3*α*,24-trihydroxyolean-12-en-28-oic acid (9); **(12)** | roots | oleanane-type triterpenoid | - | Ditto | — |
| 23 | 2*α*,3*α*,24*β*-trihydroxyurs-12-en-28-oic acid (10) | roots | ursane-type triterpenoid | - | Ditto | MCF-7,*11.01* |
| 24 | 2*α*,3*β*,23-trihydroxyurs-12-en-28-oic acid (11); **(13)** | roots | ursane-type triterpenoid | - | Ditto | HepG2,*14.22*;MCF-7,*16.99*;SK-OV-3,*28.87* |
| 25 | 2*α*,3*α*,23,24-tetrahydroxyurs-12-en-28-oic acid (12); **(4)** | roots | ursane-type triterpenoid | - | Ditto | — |
| 26 | 3*β*-hydroxyurs-12-en-28-oic acid (13) | roots | ursane-type triterpenoid | - | Ditto | A-549,*12.69*;MCF-7,*11.15* |
| 27 | 3*β*-hydroxyolean-12-en-28-oic acid (14) | roots | oleanane-type triterpenoid | - | Ditto | — |
| 28 | 2*β*,3*α*,23-trihydroxyurs-12-en-28-oic acid (15) | roots | ursane-type triterpenoid | - | Ditto | — |
| 29 | 4’-*O*-acetyl-cimigenol-3-*O*-*β*-ᴅ-xylopyranoside (16) | roots | ursane-type triterpenoid | - | Ditto | — |
| 30 | catechins-7-phytol (1) | root bark | flavonoid-diterpene conjugate | - | (Zhang et al. 2018) | — |
| 31 | 5-methoxy-coumarin-7-O-*β*-D-glycosidase (2) | root bark | coumarin glycoside | - | Ditto | the highest Anti-TMV and anti-CMV activities |
| 32 | (+)-catechins (3) | root bark | flavonoid | - | Ditto | — |
| 33 | fupenzic acid (4) | root bark | phenolic acid | - | Ditto | — |
| 34 | spathodic acid-28-O-*β*-D-glucopyranoside (5) | root bark | oleanane-type triterpenoid | - | Ditto | the second highest Anti-TMV and anti-CMV activities |
| 35 | 12-Diene-30-oic acid (6) | root bark | oleanane-type triterpenoid | - | Ditto | — |
| 36 | 3-*β*-(2-arboxy benzoyloxy)-Oleanolic acid (7) | root bark | oleanane-type triterpenoid | - | Ditto | — |
| 37 | actichinone (3-oxo-2α,24-dihydroxyurs-12-en-28-oic acid, 1) | roots | ursane-type triterpenoid | * | (Li et al. 2025) | showed most potent lipid-lowering activities against the OA-induced TG accumulation in primary hepatocytes |
| 38 | 2α,3α,24-trihydroxyurs-12-en-28-oic acid (2); **(11)** | roots | ursane-type triterpenoid | - | Ditto | showed lipid-lowering activities |
| 39 | 2α,3α,23-trihydroxyurs-12-en-28-oic acid (3) | roots | ursane-type triterpenoid | - | Ditto | showed lipid-lowering activities |
| 40 | 3*β*-(*trans-p*-coumaroyloxy)-2α,23-dihydroxyurs-12-en-28-oic  acid (jacoumaric acid, 4) | roots | ursane-type triterpenoid | - | Ditto | — |
| 41 | 2α,3*β*,24-trihydroxyurs-12-en-28-oic acid (5) | roots | ursane-type triterpenoid | - | Ditto | showed lipid-lowering activities |
| 42 | 2α,3*β*-dihydroxyurs-12-en-28-oic acid (corosolic acid, 6); | roots | ursane-type triterpenoid | - | Ditto | showed lipid-lowering activities |
| 43 | 2α,3*β*,23-trihydroxyurs-12-en-28-oic acid (asiatic acid, 7); (**13**) | roots | ursane-type triterpenoid | - | Ditto | showed lipid-lowering activities |
| 44 | 2α,3α,19α,23,24-pentahydroxyurs-12-en-28-oic acid (8) | roots | ursane-type triterpenoid | - | Ditto | — |
| 45 | 2α,3*β*,19α,23-tetrahydroxyurs-12-en-28-oic acid (9); (**20**) | roots | ursane-type triterpenoid | - | Ditto | — |
| 46 | 2α,3α,19α,24-tetrahydroxyurs-12-en-28-oic acid (10); (**17**) | roots | ursane-type triterpenoid | - | Ditto | — |
| 47 | 2α,3α,19α,23-tetrahydroxyurs-12-en-28-oic acid (11) | roots | ursane-type triterpenoid | - | Ditto | — |
| 48 | 2α,3*β*,19α-trihydroxyurs-12-en-28-oic acid (tormentic acid, 12) | roots | ursane-type triterpenoid | - | Ditto | showed lipid-lowering activities |
| 49 | 2α,3α,23-trihydroxyurs-12,20 (30)-dien-28-oic acid (13) | roots | ursane-type triterpenoid | - | Ditto | — |
| 50 | 3*β*-(trans-p-coumaroyloxy)-2α,23-dihydroxyurs-12,20(30)-dien-28-oic acid (14) | roots | ursane-type triterpenoid | - | Ditto | — |
| 51 | 2α,3*β*,23-trihydroxyurs-12,20(30)-dien-28-oic acid (actinidic acid, 15) | roots | ursane-type triterpenoid | - | Ditto | — |
| 52 | 2α,3*β*,24-trihydroxyolean-12-en-28-oic acid (16) | roots | oleanane-type triterpenoid | - | Ditto | — |
| 53 | 2α,3*β*-dihydroxyolean-12-en-28-oic acid (maslinic acid, 17) | roots | oleanane-type triterpenoid | - | Ditto | — |
| 54 | 2α,3*β*,23-trihydroxyolean-12-en-28-oic acid (arjunolic acid, 18) | roots | oleanane-type triterpenoid | - | Ditto | — |
| 55 | 2α,3α,24-trihydroxyolean-12-en-28-oic acid (19); **(12)** | roots | oleanane-type triterpenoid | - | Ditto | — |
| 56 | Protocatechoic acid (A1) | Radix | phenolic acid | - | (Nie et al. 2019) | — |
| 57 | Vanillic acid (A2) | Radix | phenolic acid | + | Ditto | — |
| 58 | *p*-Hydroxybenzaldehyde (A3) | Radix | phenol | + | Ditto | — |
| 59 | Fraxin (A4) | Radix | coumarin glycoside | - | Ditto | — |
| 60 | Trachelosperoside E1 (A5) | Radix | lignan glycoside | + | Ditto | — |
| 61 | Nigaichigoside F1 (A6) | Radix | ursane-type triterpenoid | + | Ditto | — |
| 62 | 2α,3α,19α,23,24-pentahydroxyurs-12-en-28-oic acid (A7); (**44**) | Radix | ursane-type triterpenoid | - | Ditto | — |
| 63 | 2α,3α,24-Trihydroxyurs-12-ene-23,28-dioic acid (A8) | Radix | ursane-type triterpenoid | + | Ditto | — |
| 64 | 23-Hydroxytormentic acid (A9) | Radix | ursane-type triterpenoid | - | Ditto | — |
| 65 | 2α,3*β*,19α-Trihydroxyurs-12-ene-23,28-dioic acid-23-methyl- ester (A10) | Radix | ursane-type triterpenoid | + | Ditto | — |
| 66 | 2α,3α,23,24-tetrahydroxyursa-12,20(30)-dien-28-oic acid (A11) | Radix | nor-triterpenoid | + | Ditto | — |
| 67 | Esculentic acid (A12) | Radix | ursane-type triterpenoid | - | Ditto | — |
| 68 | 3*β*,23-Dihydroxy-1-oxo-30-norolean-12,20(29)-dien-28-oicacid (A13) | Radix | nor-triterpenoid | + | Ditto | — |
| 69 | Actinidic acid (A14) (**44**) | Radix | nor-triterpenoid | - | Ditto | — |
| 70 | 3*β*,23-dihydroxy-30-norolean-12,20(29)-dien-28-oic acid (A15) | Radix | nor-triterpenoid | + | Ditto | — |
| 71 | 3-*O*-*trans*-*p*-coumaroyl tormentic acid (A16) | Radix | ursane-type triterpenoid | - | Ditto | — |
| 72 | 3-*O*-*cis*-p-coumaroyl tormentic acid (A17) | Radix | ursane-type triterpenoid | - | Ditto | — |
| 73 | Maslinic acid (A18) (**46**) | Radix | ursane-type triterpenoid | - | Ditto | — |
| 74 | (9*Z*,11*E*)-13-Hydroxy-9,11-octadecadienoic acid (A19) | Radix | Hydroxy fatty acids | + | Ditto | — |
| 75 | Ricinoleic acid (A20) | Radix | Hydroxy fatty acids | + | Ditto | — |
| 76 | Oleanic acid (A21) | Radix | oleanane-type triterpenoid | - | Ditto | — |
| 77 | 4-(*β*-D-Glucopyranosyloxy)-3-hydroxybenzoic acid (A22) | Radix | phenolic acid glycoside | + | Ditto | — |
| 78 | 5-*p*-*trans*-Coumaroylmalic acid (A23) | Radix | phenolic acid ester | - | Ditto | — |
| 79 | 5-*p-cis*-Coumaroylquinic acid (A24) | Radix | phenolic acid ester | - | Ditto | — |
| 80 | 5-Hydroxy-6-methoxy-7-*O*-*β*-D-glycopyranosylcoumarin (A25) | Radix | Coumarin glycoside | + | Ditto | — |
| 81 | Procyanidin B2 (A26) | Radix | flavonoid | - | Ditto | — |
| 82 | 4-*O-p*-Coumaroylquinic acid (A27) | Radix | phenolic acid ester | + | Ditto | — |
| 83 | Procyanidin C1 (A28) | Radix | flavonoid | + | Ditto | — |
| 84 | 6’-Acetoxy-8-*β*-D-glucopyranosyloxy-7-hydroxy-6-methoxy- coumarin (A29) | Radix | coumarin glycoside | * | Ditto | — |
| 85 | Dihydrodehydrodiconiferylalcohol 4′-*O*-*β*-Dglucopyranoside (A30) | Radix | lignan glycoside | + | Ditto | — |
| 86 | Dihydrodehydrodiconiferylalcohol 4′-*O*-*β*-Dxylopyranoside (A31) | Radix | lignan glycoside | + | Ditto | — |
| 87 | Monodictyphenone (A32) | Radix | benzophenone | + | Ditto | — |
| 88 | 12*α*-chloro-2*α*,3*β*,13*β*,23-tetrahydroxyolean-28-oic acid-13-lactone (1); (**9**) | roots | oleanane-type triterpenoid | * | (Xu 2010) | A549,*30.4*;LOVO,*31.1*;HepG2,*25.5*; |
| 89 | 2*α*,3*α*,19*α*,23,24-pentahydroxyurs-12-en-28-oic acid (2); (**44**) | roots | ursane-type triterpenoid | * | Ditto | — |
| 90 | 4,4’-dihydroxyl-dihydrochalcone-2′-O-*β*-D-glucopyranoside (3) | roots | dihydrochalcones | + | Ditto | — |
| 91 | ursolic acid (4) | roots | ursane-type triterpenoid | - | Ditto | — |
| 92 | Pseudotaraxasterol (5) | roots | ursane-type triterpenoid | + | Ditto | LOVO,*31.1*;HepG2,*33.9*; |
| 93 | 2*α*,3*β*-dihydroxyolean-12-en-28-oic acid (6); (**53**) | roots | oleanane-type triterpenoid | + | Ditto | A549,*32.7*;LOVO,*6.0*;HepG2,*34.9*; |
| 94 | 2α,3*β*-dihydroxyurs-12-en-28-oic acid (corosolic acid, 7); (**42**) | roots | ursane-type triterpenoid | + | Ditto | A549,*34.6*;LOVO,*2.9*;HepG2,*9.2*; |
| 95 | 2*α*,3*α*,24-trihydroxyurs-12-en-28-oic acid (8); **(11)** | roots | ursane-type triterpenoid | - | Ditto | A549,*34.6*;LOVO,*13.9*;HepG2,*34.5*; |
| 96 | 2*α*,3*α*,24-trihydroxyolean-12-en-28-oic acid (9); **(12)** | roots | oleanane-type triterpenoid | + | Ditto | — |
| 97 | 2*α*,3*β*,23-trihydroxyurs-12-en-28-oic acid (10); (**13**) | roots | ursane-type triterpenoid | - | Ditto | — |
| 98 | 2*α*,3*α*,23-trihydroxyurs-12-en-28-oic acid (11);(**39**) | roots | ursane-type triterpenoid | + | Ditto | A549,*32.9*;LOVO,*31.6*;HepG2,*35.7*; |
| 99 | 2*α*,3*β*,24-trihydroxyurs-12-en-28-oic acid (12);(**41**) | roots | ursane-type triterpenoid | + | Ditto | — |
| 100 | 2*α*,3*α*,19*α*,24-tetrahydroxyurs-12-en-28-oic acid (13);(**17**) | roots | ursane-type triterpenoid | - | Ditto | — |
| 101 | 2*α*,3*β*,19*α*,23-tetrahydroxyurs-12-en-28-oic acid (14);(**20**) | roots | ursane-type triterpenoid | + | Ditto | — |
| 102 | 2*α*, 3*α*, 24-trihydroxyurs-12, 20(30)-dien-28-oic acid (15) | roots | ursane-type triterpenoid | + | Ditto | — |
| 103 | 2*α*, 3*α*, 19*α*, 24-tetrahydroxyurs-12-en-28-oic acid  28-O-*β*-D-glucopyranoside (16) | roots | ursane-type triterpenoid | + | Ditto | — |
| 104 | 2*α*,3*β*,19*α*,23-tetrahydroxyurs-12-en-28-oic acid 28-O--*β*-D-glucopyranoside (T-17) | roots | ursane-type triterpenoid | + | Ditto | — |
| 105 | 2*α*,3*β*,23-trihydroxyurs-12, 20(30)-dien-28-oic acid (T-18) | roots | ursane-type triterpenoid | + | Ditto | — |
| 106 | 2*α*,3*α*,23,24-tetrahydroxyurs-12-en-28-oic acid (T-19); **(4)** | roots | ursane-type triterpenoid | + | Ditto | — |
| 107 | 2,3,4,6 tetraacetyl-(19*α*-hydroxy-2*α*, 3*α*, 24-triacetoxyurs-12-en-28-oyl)--*β*-Dglucopyranoside (T-20) | roots | ursane-type triterpenoid | - | Ditto | — |
| 108 | 2*α*,3*β*,23-trihydroxyurs-13(18)-en-28-oic acid (T-21) | roots | ursane-type triterpenoid | * | Ditto | — |
| 109 | 1*α*,2*α*,3*β*,19*α*,23-pentahydroxyurs-12-en-28-oic acid (T-22) | roots | ursane-type triterpenoid | + | Ditto | — |
| 110 | Actinidin A (T-23) | roots | cerebroside | + | Ditto | — |
| 111 | Actinidin B (T-24) | roots | cerebroside | + | Ditto | — |
| 112 | 3*β*-hydroxystigmast-5-en-7-one (T-25) | roots | steroid | + | Ditto | — |
| 113 | *β*-sitosterol (T-26) | roots | steroid | - | Ditto | — |
| 114 | daucosterol (T-27) | roots | steroid | - | Ditto | — |
| 115 | n-dotriacontanic acid (T-28); (**7**) | roots | fatty acid | + | Ditto | — |
| 116 | ursolic acid (Ⅰ); (**91**) | root | ursane-type triterpenoid | - | (He et al. 2016) | — |
| 117 | Daucosterol (Ⅱ); (**114**) | root | phytosterol glycoside | - | Ditto | — |
| 118 | 2α,3α,24-trihydroxyurs-12-en-28-oic acid (Ⅲ); **(11)** | root | triterpenoid | - | Ditto | — |
| 119 | Formononetin（Ⅳ） | root | isoflavone | - | Ditto | — |
| 120 | 7,4’-dihydroxyflavone（Ⅴ） | root | flavone | + | Ditto | — |
| 121 | (Z)-9,10,11-trihydroxy-12-octadecenoic acid（Ⅵ） | root | hydroxy fatty acid | + | Ditto | — |
| 122 | 2α,3α,19α,24-tetrahydroxyurs-12-en-28-oic acid (Ⅶ); (**17**) | root | triterpenoid | - | Ditto | — |
| 123 | Syringaldehyde (Ⅷ) | root | phenylpropanoid | + | Ditto | — |
| 124 | *β*-sitosterol (Ⅰ); (**113**) | root | phytosterol glycoside | - | (He et al. 2015a) | — |
| 125 | ursolic acid (Ⅱ); (**91**) | root | ursane-type triterpenoid | - | Ditto | — |
| 126 | oleanolic acid (Ⅲ) | root | oleanane-type triterpenoid | - | Ditto | — |
| 127 | Daucosterol (Ⅳ); (**114**) | root | phytosterol glycoside | - | Ditto | — |
| 128 | coumalic acid（Ⅴ） | root | coumarin | + | Ditto | — |
| 129 | *p*-hydroxybenzoic acid（Ⅵ） | root | phenolic acids | + | Ditto | — |
| 130 | indole-3-carboxylic acid（Ⅶ） | root | Indole alkaloid | + | Ditto | — |
| 131 | 2α,3α,24-trihydroxyurs-12-en-28-oic acid (Ⅷ); (11) | root | ursane-type triterpenoid | - | Ditto | — |
| 132 | 2α,3α,19α,24-tetrahydroxyurs-12-en-28-oic acid (Ⅸ); (**17**) | root | ursane-type triterpenoid | - | Ditto | — |
| 133 | 2α,3α,24-trihydroxyolean-12-en-28-oic acid (Ⅹ); **(12)** | root | oleanane-type triterpenoid | - | Ditto | — |
| 134 | chlorogenic acid (1) | root | phenolic acid | # | (He et al. 2015b) | — |
| 135 | crptochlorogenic acid (2) | root | phenolic acid | # | Ditto | — |
| 136 | neochlorogenic acid (3) | root | phenolic acid | # | Ditto | — |
| 137 | 3-*O*-coumaroylquinic acid ( 4) | root | phenolic acid | # | Ditto | — |
| 138 | caffeic acid ( 5) | root | phenolic acid | + | Ditto | — |
| 139 | *trans-p*-hydroxycinnamic acid ( 6) | root | phenolic acid | # | Ditto | — |
| 140 | esculin (7) | root | coumarin glycoside | - | Ditto | — |
| 141 | scopoletin (8) | root | coumarin | # | Ditto | — |
| 142 | scopolin (9) | root | coumarin glycoside | # | Ditto | — |
| 143 | isofraxoside (10) | root | iridoid glycoside | # | Ditto | — |
| 144 | (－) -epi-catechin (1); (**155**) | roots | flavanol | - | (He et al. 2014) | — |
| 145 | ( + ) -catechin (2); (**32**) | roots | flavanol | - | Ditto | — |
| 146 | fraxetin (3） | roots | coumarin | # | Ditto | — |
| 147 | escaletin (4) | roots | coumarin | # | Ditto | — |
| 148 | protocatechuic aldehyde (5) | roots | phenolic aldehyde | # | Ditto | — |
| 149 | (－) -epi-catechin-5-O-*β*-D-glucopyranoside (6) | roots | flavanol glycoside | # | Ditto | — |
| 150 | *erythro*-1,2-bis-(4-hydroxy-3-methoxyphenyl)-1,3-propanediol (7) | roots | lignan | # | Ditto | — |
| 151 | *threo-*1,2-bis-(4-hydroxy-3-methoxyphenyl)-1,3-propanediol (8) | roots | lignan | # | Ditto | — |
| 152 | γ-quinide (1) | root | iridoid | * | (Chen et al. 2011) | — |
| 153 | stearyl-*β*-D-glucopyranoside (2) | root | glycolipid | # | Ditto | — |
| 154 | daucosterol (3); (**114**) | root | phytosterol glycoside | - | Ditto | — |
| 155 | (−)-*epi*-catechin (4); | root | flavanol | - | Ditto | — |
| 156 | 2*α*,3*α*,24-trihydr-oxyurs-12-en-28-oic acid (5) | root | ursane-type triterpenoid | - | Ditto | — |
| 157 | 3-*epi*-corosolic acid (6) | root | ursane-type triterpenoid | - | Ditto | — |
| 158 | ursolic acid (7); (**91**) | root | ursane-type triterpenoid | - | Ditto | — |
| 159 | n-butyl-*β*-D-fructopyranoside (8) | root | alkyl glycoside | # | Ditto | — |
| 160 | sucrose (9) | root | disaccharide | - | Ditto | — |
| 161 | lignocericacid (10) | root | saturated fatty acid | # | Ditto | — |
| 162 | *β*-sitosterol (11) ; (**113**) | root | phytosterol | - | Ditto | — |
| 163 | (+)-Catechin (1) ; (**32**) | roots | flavanol | + | (Zhou et al. 2010) | — |
| 164 | (–)*-epi*-Catechin (2); (**155**) | roots | flavanol | + | Ditto | — |
| 165 | Tachioside (3) | roots | phenolic glycoside | + | Ditto | — |
| 166 | Isotachioside (4) | roots | phenolic glycoside | + | Ditto | — |
| 167 | 5-Hydroxy-6-methoxy-7-O-*β*-D-glucosyl coumarin (5) | roots | coumarin glycoside | + | Ditto | — |
| 168 | Fraxin (6);(**59**) | roots | coumarin glycoside | + | Ditto | — |
| 169 | Vanillic acid (7); (**57**) | roots | Phenolic Acid | + | Ditto | — |
| 170 | 1-O-(*β*-D-Glucosyl)-2-[2-methoxy-4-(ω-hydroxypropyl)-phen oxy]- propan-3-ol (8) | roots | phenolic glycoside | + | Ditto | — |
| 171 | n-Butyl-*β*-D-fructopyranoside (9); (**159**) | roots | alkyl glycoside | - | Ditto | — |
| 172 | uracil (10) | roots | pyrimidine alkaloid | - | Ditto | — |
| 173 | adenine (11) | roots | purine alkaloid | - | Ditto | — |
| 174 | myo-inositol (12) | roots | cyclohexanhexol | - | Ditto | — |
| 175 | *β*-sitosterol (13) ; (**113**) | roots | phytosterol | - | Ditto | — |
| 176 | daucosterol (14); (**114**) | roots | steroidal glycoside | - | Ditto | — |
| 177 | 2*α*,3*α*,24-trihydroxyolean-12-en-23-al-28-oic acid (1) | Twigs, leaves | oleanane-type triterpenoid | * | (Zhang et al. 2022) | — |
| 178 | 2*α*,3*α*,24-trihydroxyolean-12-en-28-oic acid (2); **(12)** | Ditto | oleanane-type triterpenoid | - | Ditto | — |
| 179 | 3*β*-hydroxyolean-12-en-28-oic acid (3); (**27**) | Ditto | oleanane-type triterpenoid | - | Ditto | — |
| 180 | goreishic acid I (4) | Ditto | ursane-type triterpenoid | # | Ditto | — |
| 181 | 2*α*,3*α*,24-trihydroxy-12-ursene-23-al-28-oic acid (5) | Ditto | ursane-type triterpenoid | # | Ditto | — |
| 182 | tormentic acid (6); (**41**) | Ditto | ursane-type triterpenoid | - | Ditto | — |
| 183 | 2α,3α,23-trihydroxyurs-12-en-28-oic acid (7);(**39**) | Ditto | ursane-type triterpenoid | - | Ditto | — |
| 184 | ursa-12,20(30)-dien-28-oic acid (8) | Ditto | ursane-type triterpenoid | # | Ditto | — |
| 185 | 2α,3α,23-trihydroxyursa-l2,20(30)-dien-28-oic acid (9) | Ditto | ursane-type triterpenoid | - | Ditto | — |
| 186 | 3-O-*trans*-*p*-coumaroyl actinidic acid (10) | Ditto | ursane-type triterpenoid | - | Ditto | — |
| 187 | 30-nor-2α,3*β*-dihydoxyurs-12-ene (11) | Ditto | nor-triterpenoid | # | Ditto | — |
| 188 | orthosiphol F (12) | Ditto | diterpenoid | # | Ditto | — |
| 189 | naringenin (13) | Ditto | flavone | # | Ditto | — |
| 190 | luteolin (14) | Ditto | flavonoid | # | Ditto | — |
| 191 | daidzein (15) | Ditto | isoflavone | # | Ditto | — |
| 192 | balanophonin B (16) | Ditto | lignan | # | Ditto | — |
| 193 | coumarino-lignan (17) | Ditto | coumarin-lignan hybrid | # | Ditto | — |
| 194 | anofinic acid (18) | Ditto | phenolic acid | # | Ditto | — |
| 195 | 6-formyl-2,2-dimethyl-2H-chromene (19) | Ditto | chromene | # | Ditto | — |
| 196 | leptoic acid A (20) | Ditto | prenylated benzoic acid | # | Ditto | — |
| 197 | 3*β*-acetyloxyursolic acid (1) | fruits | ursane-type triterpenoid | + | (Ma et al. 2016) | — |
| 198 | 2*α*,3*β*-dihydroxyurs-12-en-28-oic acid (2) (**43**) | fruits | ursane-type triterpenoid | + | Ditto | — |
| 199 | 2*α*,3*α*-dihydroxyurs-12-en-28-oic acid (3) | fruits | ursane-type triterpenoid | + | Ditto | — |
| 200 | 2*α*,3*α*-dihydroxyolean-12-en-28-oic acid (4) ， | fruits | oleanane-type triterpenoid | + | Ditto | — |
| 201 | oleanolic acid (5); (**126**) | fruits | oleanane-type triterpenoid | - | Ditto | — |
| 202 | ursolic acid (6); (**91**) | fruits | ursane-type triterpenoid | - | Ditto | — |
| 203 | daucosterol (7); (**114**) | fruits | phytosterol glycoside | - | Ditto | — |
| 204 | *β*-sitosterol (8); (**113**) | fruits | phytosterol | - | Ditto | — |
| 205 | protocatechuic acid (1) | seeds | phenolic acid | - | (Zhang et al. 2019) | — |
| 206 | vanillic acid (2) (**70**) | seeds | phenolic acid | - | Ditto | — |
| 207 | syringic acid (3) | seeds | phenolic acid | - | Ditto | — |
| 208 | protocatechuic aldehyde (4) (**187**) | seeds | phenolic aldehyde | - | Ditto | — |
| 209 | 3,4-dihydroxyace to phenone. ethyl ketone (5) | seeds | phenolic ketone | + | Ditto | — |
| 210 | dihydroconiferyl alcohol (6) | seeds | phenylpropanoid | + | Ditto | — |
| 211 | 4, 4-dihydroxydiphenylmethane (7), | seeds | diarylmethane | + | Ditto | — |
| 212 | shikimic acid (8) | seeds | alicyclic organic acid | - | Ditto | — |
| 213 | ferulic acid (9) | seeds | phenolic acid | - | Ditto | — |
| 214 | 3-acetoxymethyl-4-methoxy methyl benzoate (10) | seeds | phenolic acid ester | + | Ditto | — |
| 215 | bis(4-methoxyphenyl) methane (11) | seeds | diarylmethane | + | Ditto | — |
| 216 | methyl caffeate (12) | seeds | phenolic acid ester | + | Ditto | — |
| 217 | sinensin (1) | leaves | dihydroflavonol glycoside | - | (Bai et al. 2022) | — |
| 218 | naringin (2) | leaves | dihydroflavonol glycoside | - | Ditto | — |
| 219 | rutin (3) | leaves | flavonol glycoside | - | Ditto | strong scavenging effects on DPPH and ABTS free radicals; scavenging effect on PTIO free radicals. |
| 220 | quercitrin (4) | leaves | flavonol glycoside | - | Ditto | strong scavenging effects on DPPH and ABTS free radicals; |
| 221 | kaempferol 7-O-*β-*D-glucoside (5) | leaves | flavonol glycoside | - | Ditto | strong scavenging effects on DPPH and ABTS free radicals; scavenging effect on PTIO free radicals. |
| 222 | 2*α*,3*α*,24-trihydroxy-12-en-28-oleanolic acid (6) (**9**) | leaves | oleanane-type triterpenoid | - | Ditto | — |
| 223 | quercetin 7-*β*-D-glucopyranoside (7) | leaves | flavonol glycoside | - | Ditto | strong scavenging effects on DPPH and ABTS free radicals; scavenging effect on PTIO free radicals. |
| 224 | myricitrin (8) | leaves | flavonol glycoside | - | Ditto | strong scavenging effects on DPPH and ABTS free radicals; scavenging effect on PTIO free radicals. |
| 225 | isoquercitrin (9) | leaves | flavonol glycoside | - | Ditto | strong scavenging effects on DPPH and ABTS free radicals; |
| 226 | acantrifoside E (10) | leaves | triterpenoid glycoside | - | Ditto | — |
| 227 | taxifolin 3′-O-glucoside (11) | leaves | dihydroflavonol glycoside | - | Ditto | — |
| 228 | 3-prenyl-4-O-β-D-glucopyranosyloxy-4-hydroxybenzoic acid (12) | leaves | prenylated phenolic acid glycoside | - | Ditto | — |

Note: New compound (*); first reported from the genus *Actinidia* (#); first isolated from *Actinidia chinensis* (+); other known compounds (-); — indicates no biological activity reported. Duplicate compounds among different literature datasets are marked and highlighted in blue text.
