# Supplementary material for: From genomics to horticultural innovation: botanical traits, bioactive compounds, and sustainable utilization of kiwifruit (Actinidia spp.)
Source: Front Plant Sci. 2026 Aug 17;17:1903424. doi: 10.3389/fpls.2026.1903424 (PMC13528668; doi:10.3389/fpls.2026.1903424)
Supplement: Supplementary file 2 [file Table2.docx]

| S.Table S2. Comparative overview of core phytochemical classes, representative commercial kiwi cultivars from major global producing regions, and their structure–activity relationships & functional value | | | | | |
| --- | --- | --- | --- | --- | --- |
| **Phytochemical Class** | **Core Subclasses & Biosynthetic Traits** | **Global Cultivar & Origin** | **Key Structure–Activity Relationships (SAR)** | **Primary Biological & Industrial Value** | **Ref** |
| L-Ascorbic acid & actinidin | L-ascorbate (VC); cysteine-specific protease actinidin; accumulation differs greatly between gold and green kiwi flesh | New Zealand ‘SunGold’; traditional European green kiwi | 1. Ascorbic acid level positively correlates with antioxidant capacity and iron absorption;  2. Actinidin exerts broad proteolytic activity across gastrointestinal pH | Boost immunity, relieve fatigue; meat tenderizer for food processing; improve intestinal discomfort | (Richardson D.P., et al. 2018) |
| Polysaccharides (dietary fibre) | Pectin (soluble fibre), cellulose/ hemicellulose; gold kiwi contains less total fibre than green varieties | Late-maturing Hayward from Chile & Europe | Soluble pectin retains water and promotes short-chain fatty acid production in colon | Regulate gut microbiota, relieve constipation, low-GI food raw material | (Richardson D.P., et al. 2018) |
| Triterpenoids | Ursane & oleanane triterpenes (summarized in Table S1); coexist with fruit phenols | Chilean Hayward, Italian Gold3 | Synergistic antioxidant effect with polyphenols; hydroxylation enhances bioactivity | Lipid-regulating and anti-tumor by-product extracts |  |
| Flavonoids & phenolic acids | Chlorogenic acid and total polyphenols; phenolic accumulation varies by shoot type and growing condition | Southern Italian Gold3; Chilean A. deliciosa ‘Hayward’ | Higher total polyphenol content leads to stronger total antioxidant capacity | Natural antioxidant raw material; inhibit fruit browning, anti-inflammatory dietary supplements | (Dattola A, et al. 2024) |

Note:

1.All monomer classification statistics of kiwi phytochemicals are derived from Supplementary Table 1 (Ref 25–40), which mainly collates chemical data of Chinese native Actinidia germplasm. This table only summarizes overseas commercial cultivars to achieve balanced cross-regional comparison.

2. Nutritional composition, VC, actinidin and dietary fibre traits of New Zealand & European varieties are supported by Richardson et al. (2018); total polyphenol antioxidant indicators of Italian ‘Gold3’ and Chilean ‘Hayward’ are extracted from Dattola et al. (2024).

3.The structural classification, hydroxylation activity rule and lipid-lowering function of ursane-type triterpenes are fully summarized from Li et al. (2025) and other Chinese literature listed in Table S1; Chilean ‘Hayward’ and Italian ‘Gold3’ are only representative overseas cultivar examples, no dedicated foreign literature reports fruit triterpenoid quantification data for these two varieties.
